# Activation of secondary metabolite gene clusters in *Chaetomium olivaceum* via the deletion of a histone deacetylase

**DOI:** 10.1007/s00253-024-13173-8

**Published:** 2024-05-11

**Authors:** Peipei Zhao, Shengling Cao, Jiahui Wang, Jiaying Lin, Yunzeng Zhang, Chengwei Liu, Hairong Liu, Qingqing Zhang, Mengmeng Wang, Yiwei Meng, Xin Yin, Jun Qi, Lixin Zhang, Xuekui Xia

**Affiliations:** 1 Biology Institute, Qilu University of Technology (Shandong Academy of Sciences), Jinan, 250103 Shandong China; 2 Department of Thoracic Surgery, Shandong Public Health Clinical Center, Jinan, 250013 Shandong China; 3 Key Laboratory for Enzyme and Enzyme-Like Material Engineering of Heilongjiang, College of Life Science, Northeast Forestry University, Harbin, 150040 Heilongjiang China; 4 State Key Laboratory of Bioreactor Engineering, School of Biotechnology, East China University of Science and Technology (ECUST), Shanghai, 200237 China

**Keywords:** *Chaetomium olivaceum*, Epigenetic regulation, HDAC, Biosynthetic gene clusters, Polyketide synthase

## Abstract

**Abstract:**

Histone acetylation modifications in filamentous fungi play a crucial role in epigenetic gene regulation and are closely linked to the transcription of secondary metabolite (SM) biosynthetic gene clusters (BGCs). Histone deacetylases (HDACs) play a pivotal role in determining the extent of histone acetylation modifications and act as triggers for the expression activity of target BGCs. The genus *Chaetomium* is widely recognized as a rich source of novel and bioactive SMs. Deletion of a class I HDAC gene of *Chaetomium olivaceum* SD-80A, *g7489*, induces a substantial pleiotropic effect on the expression of SM BGCs. The *C. olivaceum* SD-80A ∆*g7489* strain exhibited significant changes in morphology, sporulation ability, and secondary metabolic profile, resulting in the emergence of new compound peaks. Notably, three polyketides (**A1**–**A3**) and one asterriquinone (**A4**) were isolated from this mutant strain. Furthermore, our study explored the BGCs of **A1**–**A4**, confirming the function of two polyketide synthases (PKSs). Collectively, our findings highlight the promising potential of molecular epigenetic approaches for the elucidation of novel active compounds and their biosynthetic elements in *Chaetomium* species. This finding holds great significance for the exploration and utilization of *Chaetomium* resources.

**Key points:**

*• Deletion of a class I histone deacetylase activated secondary metabolite gene clusters.*

*• Three polyketides and one asterriquinone were isolated from HDAC deleted strain.*

*• Two different PKSs were reported in C. olivaceum SD-80A.*

**Supplementary Information:**

The online version contains supplementary material available at 10.1007/s00253-024-13173-8.

## Introduction

Filamentous fungi harbor numerous biosynthetic gene clusters (BGCs) responsible for the biosynthesis of secondary metabolites (SMs). The diversity within these BGCs offers significant potential for the synthesis of natural products. Several clinically employed antibacterials, antifungal agents, immunosuppressants, and antihypercholesterolemic drugs, including penicillin, anidulafungin, caspofungin, and lovastatin, originate from fungal natural products (Hoffmeister and Keller 2007; Newman and Cragg 2020). *Chaetomium*, a large genus within the fungal family Chaetomiaceae, is widely widespread in soil, air, and plants, and possesses both ecological and economic importance (Jiang et al. 2016). This fungus has been reported to synthesize more than 200 compounds, including depsidones, chaetoglobosins, terpenoids, and azaphilones (Zhang et al. 2012), many of which possess antimicrobial, antifungal, antitumor, and cytotoxic properties, among others (Zhang et al. 2012).

However, a significant proportion of these BGCs either remains unexpressed or exhibit very low expression under unnatural conditions. Therefore, activating the expression of these BGCs and acquiring the active substances are of great practical value (Rutledge and Challis 2015). Epigenetic modification of BGCs plays a crucial role in the biosynthesis of SMs. This process involves crucial mechanisms like DNA methylation and histone posttranslational modifications. Histones, which are the scaffolding proteins of eukaryotic chromosomes, directly influence the transcriptional activity of chromosomal DNA (Poças-Fonseca et al. 2020). Histones can be modified through multiple molecular mechanisms, including acetylation, methylation, phosphorylation, ubiquitination, adenylation, and ADP ribosylation, which collectively impact the transcriptional state of BGCs responsible for SM synthesis (Lai et al. 2022). Among these, histone acetylation and deacetylation are closely linked to the activation and silencing of gene transcription.

In *Aspergillus nidulans*, the knockout of the histone deacetylase (HDAC) gene *hdaA* successfully activated two secondary metabolic BGCs (Shwab et al. 2007). Additionally, inhibiting HDAC with hydroxamic acid has been reported to elevate the acetylation level of fungal SM genes, leading to the production of novel compounds (Asai et al. 2013). The knockout of the HDAC gene *hdaA* can activate up to 75% of SM synthetic genes in *Calcarisporium arbuscula*, resulting in the production of new structural compounds (Mao et al. 2015). Moreover, the elimination of *hdaA* from *Penicillium chrysogenum* enhances the activity of sorbicillinoids and meleagin/roquefortine BGCs, while downregulating the expression of naphtha-γ-pyrone and chrysogine synthetic genes (Ding et al. 2020; Guzman-Chavez et al. 2018). Deletion of a putative HDAC gene, *hid1*, in *Pestalotiopsis microspore* resulted in a twofold increase in the production of pestalotiollide B. (Niu et al. 2015). For the histone acetylation modification research on *Chaetomium*, chemical epigenetic modification using the NAD^+^-dependent HDAC inhibitor nicotinamide has been shown to stimulate the production of polyketides in *Chaetomium cancroideum* and *Chaetomium mollipilium* (Asai et al. 2012, 2016). Overexpression of histone acetyltransferases (HATs) activates dimeric bis-spiro-azaphilone BGC in *Chaetomium globosum* CBS148.51, and the deletion of HAT CgSptJ in this species results in the production of mollipilin A and B (Nakazawa et al. 2013; Wang et al. 2017). However, due to the inherently high non-homologous random recombination activity of *Chaetomium*, many *Chaetomium* strains are not suitable for molecular genetic manipulations, particularly gene knockout experiments (Wang et al. 2017). Moreover, the activation of silent BGCs in *Chaetomium* through HDAC knockout has not been reported, which severely limits the exploitation of compound and gene resources in *Chaetomium*.

*Chaetomium olivaceum* SD-80A was selected for further investigation due to its potent anti-methicillin-resistant *Staphylococcus aureus* (MRSA) activity, exhibiting a minimum inhibitory concentration (MIC) of 0.78 μg/mL. In a previous study, we isolated four compounds from the fermentation of *C. olivaceum* SD-80A, two of which demonstrated moderate bioactivity against *S. aureus* (SA) and MRSA (Wang et al. 2020). However, the number of compounds discovered from the strain is much lower than the number of BGCs predicted in silico. Based on these previous findings, our study sought to explore the use of genetic manipulation centered on HDAC genes as an effective strategy to activate fungal BGCs. Here, we performed knockout of the class I HDAC gene, *g7489*, in *C. olivaceum* SD-80A and explored the influence of its inactivation on secondary metabolism. Our findings revealed that the ∆*g7489* strain exhibited significant alterations in morphology, sporulation ability, and the secondary metabolic profile, accompanied by the appearance of novel compound peaks. The differentially expressed compounds were identified as three polyketides (**A1**–**A3**) and one asterriquinone (**A4**). Heterologous expression in *Aspergillus oryzae* was then employed, which led to the identification of two polyketide synthases (PKSs) and an orsellinic acid (OA) derivative. Furthermore, we investigated the effect of HDAC on the SM biosynthetic core genes of *C. olivaceum* SD-80A. Therefore, this study not only sheds light on the regulatory role of HDAC in fungal secondary metabolism but also provides an effective strategy for the continued exploration and exploitation of *Chaetomium*-derived resources.

## Materials and methods

### Fungal material and growth media

The fungal strain *C. olivaceum* SD-80A was isolated from nilgai (*Boselaphus tragocamelus*) feces collected in New Delhi, India, and deposited at the Biology Institute, Shandong Academy of Sciences, Jinan, China and the China General Microbiological Culture Collection Center (CGMCC) (accession no. 40420). Daily culture was conducted using PDA and PDB medium.

### Bioinformatics analysis

The genomic sequencing of *C. olivaceum* SD-80A was carried out on a PacBio Sequel instrument and subsequently assembled *de novo* with the Hierarchical Genome Assembly Process 3 (HGAP3) (Chin et al. 2013). The data was deposited in GenBank with accession no. PRJNA1065967. AntiSMASH and 2ndFind were used to predict the BGCs (Blin et al. 2023). Gene function prediction was conducted using the NCBI database. AUGUSTUS was used for gene prediction. Pfam was used for domain prediction (Bachmann and Ravel 2009). The Enzyme Function Initiative-Enzyme Similarity Tool (EFI-EST) was employed for constructing sequence similarity networks (SSNs) of proteins (Gerlt et al. 2015). Sequence alignment and phylogenetic analyses were performed with the MEGA 7.0 software by maximum likelihood method. The resulting phylogenetic tree was visualized using iTOL (Letunic and Bork 2021).

### Generation of fungal strains

∆*g7489* of *C. olivaceum* SD-80A was constructed as Szewczyk et al. described based on homologous recombination (Szewczyk et al. 2006). The flanking regions of the target gene were amplified from *C. olivaceum* SD-80A genome (Aidlab Biotechnologies Co. Ltd) and fused with the selection marker *hygB*.

*C. olivaceum* SD-80A was inoculated on a sporulation medium and cultured at 28 °C for 14 days. The spores were suspended in 100 mL PDB medium and shaken overnight at 28 °C. Transformations were carried out as described by Nakazawa et al. with minor modification (Nakazawa et al. 2013). Briefly, the mycelium was collected by filtration, washed three times with 15 mL of osmotic medium (OM), suspended in 15 mL OM containing 30 mg lysing enzymes (Sigma) and 20 mg Yatalase (Takara), and incubated at 28 °C for 4 h. The resulting protoplasts were filtered, added with 20 mL of protoplast trapping buffer, collected by centrifuging, resuspended in 300 μL STC buffer, and divided into tubes with 100 μL each. Five micrograms of DNA fragments was added to the protoplasts, incubated on ice for 40 min, added with 600 µL of PEG solution and incubated at room temperature for another 20 min. The mixture was plated on MYG–sorbitol agar medium with suitable antibiotics (200 μg/mL hygromycin B or G418). The genotype of the deletion mutant was confirmed via diagnostic PCR. For the complementation of *g7489* (∆*g7489*-C), the ∆*g7489* strain was co-transformed with a fragment containing the *g7489* encoding sequence including the *PtrpC* promoter and a fragment comprising a geneticin selection marker G418, respectively. The primers are summarized in Table S1.

### SM analysis of *C. olivaceum* SD-80A

The wild-type (WT) strain and ∆*g7489* mutant were grown on PDA medium for 7 days. The mycelium from a Petri dish was transferred into three flasks, each containing 100 mL of PDB medium, for a 5-day seed culture period. Subsequently, the seed culture was inoculated into 30 flasks (60 mL of water and 40 g of rice per flask) for 30 days at 28 °C. The fermentation was extracted using a 1:1 volume ratio of ethyl acetate (EtOAc), and a residue was obtained after rotary evaporation at 38 °C. The solids obtained were dissolved in chromatographic grade methanol and analyzed by high-performance liquid chromatography (HPLC). The extracts were purified using silica gel column chromatography with eluents of petroleum ether: EtOAc/MeOH (methanol) (100:0:0, 80:20:0, 60:40:0, 40:60:0, 0:100:0, 0:90:10, 0:50:50, and 0:0:100, v/v) to yield eight fractions (Fr.1–8). Fr. 3 was subjected to fractionation on a Sephadex LH-20 column using an isocratic elution of dichloromethane (DCM)/MeOH (1:1) and produced five subfractions (3.1–3.5). Subfraction 3.3 underwent further fractionation by HPLC (Agilent C_18_ 5 μm 10 × 250 mm, 2 mL/min, isocratic elution 53% MeOH/H_2_O) to collect compound **1** (**A1**). Fr. 4 was eluted under the same conditions as Fr. 3 and yielded six subfractions (4.1–4.6). Subfraction 4.5 was further fractionated into subfractions by HPLC to collect compounds **2** (**A2**), **3** (**A3**), and **4** (**A4**). The nuclear magnetic resonance (NMR) spectra of the compounds were recorded on a Bruker Biospin Avance 400 spectrometer. High-resolution-electrospray ionization-mass spectrometry (HR-ESI–MS) or ESI–MS data were recorded on an Agilent 6230 mass spectrometer (Agilent, USA).

### Real-time PCR analysis

The fungi grown under the above-described fermentation conditions were harvested. Total RNA was extracted using Trizol. cDNA was synthesized with the HiScript III RT SuperMix for qPCR (+ gDNA wiper) (Vazyme, China). Real-time PCR was conducted on a Pangaea 6 Real-Time System (Apexbio, China). Three replicates of each cDNA sample were performed, and the average threshold cycle was calculated. Relative expression levels were determined via the 2^−∆∆Ct^ method. Actin was used as the reference gene. Statistical analyses were performed using Microsoft Excel.

### Heterologous expression in *A. oryzae* and metabolite detection

Genome DNA of *C. olivaceum* SD-80A was used to amplify PKS genes *g657*, *g4635* and cytochrome P450 gene *g656*. The PCR products were inserted into linearized pUARA2 and pAdeA2 (treated by *Kpn*I) using the ClonExpress Ultra One Step Cloning Kit (Vazyme, China) to generate the expression plasmids pUARA-N2.2, pUARA-N15.1, and pAdeA-P450. *A. oryzae* NSAR1 (*niaD*^−^, *sC*^−^, *∆argB*, *adeA*^−^) was used as expression host. The transformations of *A. oryzae* were carried out as Tagami et al. described (Tagami et al. 2013).

Transformants were cultured in 10 mL MPY medium for 3 days at 30 °C, extracted three times with EtOAc, and the residue was obtained after rotary evaporation at 38 °C and 50 hPa. The residue was dissolved in chromatographic methanol and analyzed by HPLC (YMC-Pack ODS-A column, 10 × 250 mm, 5 μm). The HPLC analyses were conducted under the following conditions: gradient elution with 0.1% trifluoroacetic acid in H_2_O/MeCN (methyl cyanide) 95:5 to 100% MeCN in 30 min, MeCN 100% for 12 min, and a flow rate of 1 mL/min (Lackner et al. 2013).

## Results

### Gene sequencing and biological information mining

The genome of *C. olivaceum* SD-80A comprises 117 contigs, with a total length of 36,041,079 base pairs (bp) and a G + C content of 55.60%. Notably, the *C. olivaceum* genome harbors 46 BGCs responsible for the biosynthesis of SMs (Table S2). Among these clusters, we identified 14 type I PKS (T1PKS) clusters, 8 non-ribosomal peptide synthetase (NRPS) clusters, and 5 terpene clusters, along with other type clusters.

The HDAC protein (g7489) was identified through a BLAST search conducted in *C. olivaceum* SD-80A, using the sequence of the HDAC protein (XP_006695713.1) reported in *Thermochaetoides thermophila* DSM 1495. The g7489 protein, encoded by a 1491 bp genomic sequence containing no intron, comprises 496 amino acids and features a conserved domain of the arginase_HDAC superfamily. The nucleotide sequence was deposited in GenBank with accession number PP498810. Significantly, this protein is homologous to the class I HDAC Hos2, sharing a high degree of similarity with orthologs from *T. thermophila* (82.73% identity) and *Neurospora crassa* (75.91% identity), respectively. A phylogenetic analysis further revealed that g7489 and its orthologs across diverse fungal species exhibit evolutionary conservation (Fig. S1).

### Generation of the HDAC gene knockout mutant strain and its complementation in *C. olivaceum* SD-80A

Deletion mutants were generated by replacing the HDAC gene *g7489* with the hygromycin-resistance gene, serving as a selectable marker, in the WT strain of *C. olivaceum* SD-80A (Fig. S2a). Deletion mutants were identified through PCR analysis using five primer pairs 5-F-out/3-R-out, 5-F-out/Y-HygR-R, Y-HygR-DF/3-R-out, PJ007/PJ008, and F-in/R-in (Fig. S2b, Table S1). The ∆*g7489* mutants were further verified through PCR product sequencing. To complement the strain, transformants (∆*g7489*-C1-C6) were created by introducing the target gene with the *PtrpC* promoter into the genome of the ∆*g7489* mutant strain. Verification was conducted by screening for geneticin resistance and PCR targeting the *g7489* gene using the PJ007/g7489-R-out primer pair (Fig. S2c, Table S1).

### Effect of *g7489 *deletion on the phenotype, growth, and SMs of *C. olivaceum* SD-80A

**Fig. 1:**
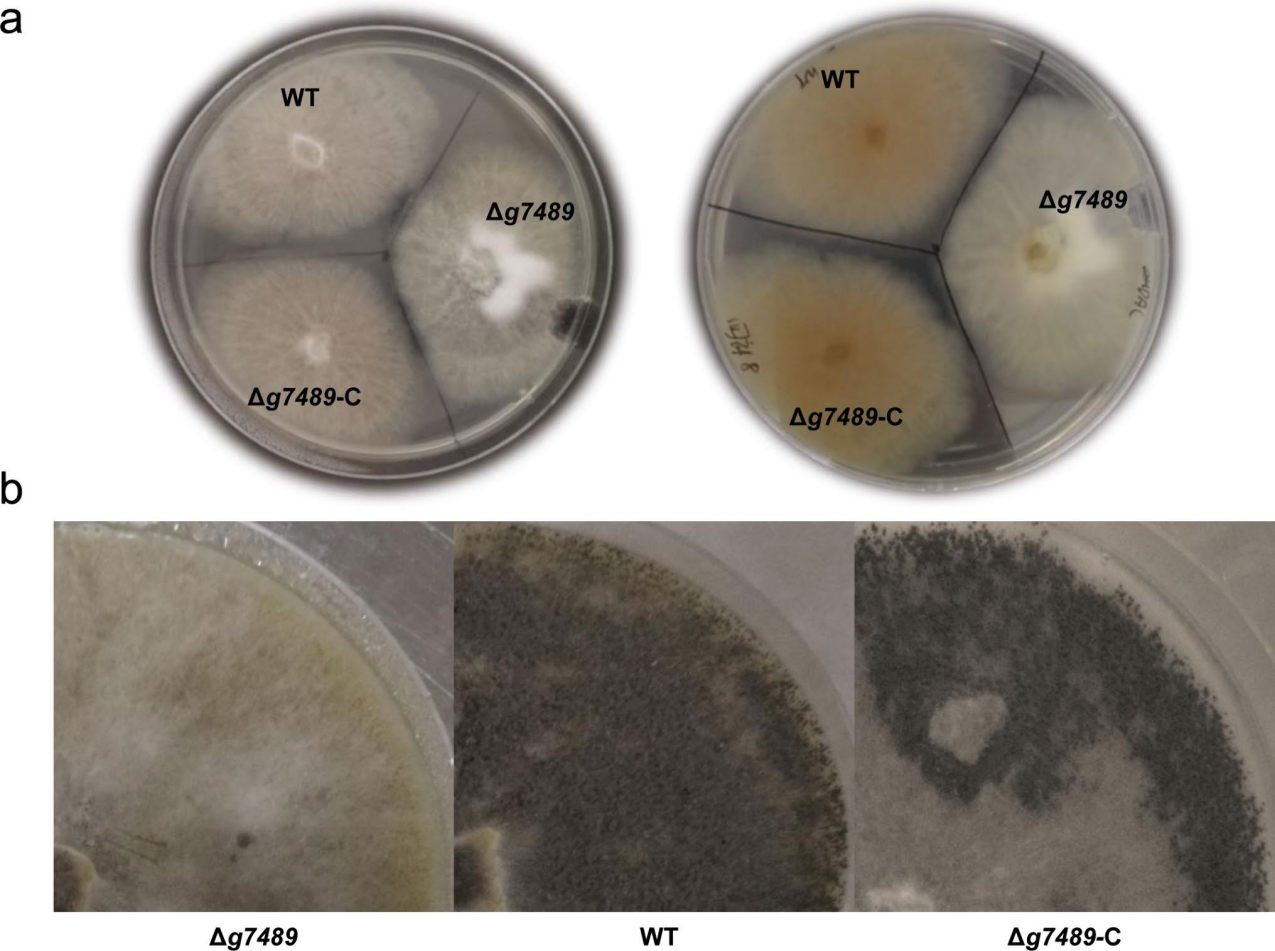
The phenotype of *C. olivaceum* SD-80A strains grown on PDA (**a**) and sporulation medium (**b**)

**Fig. 2:**
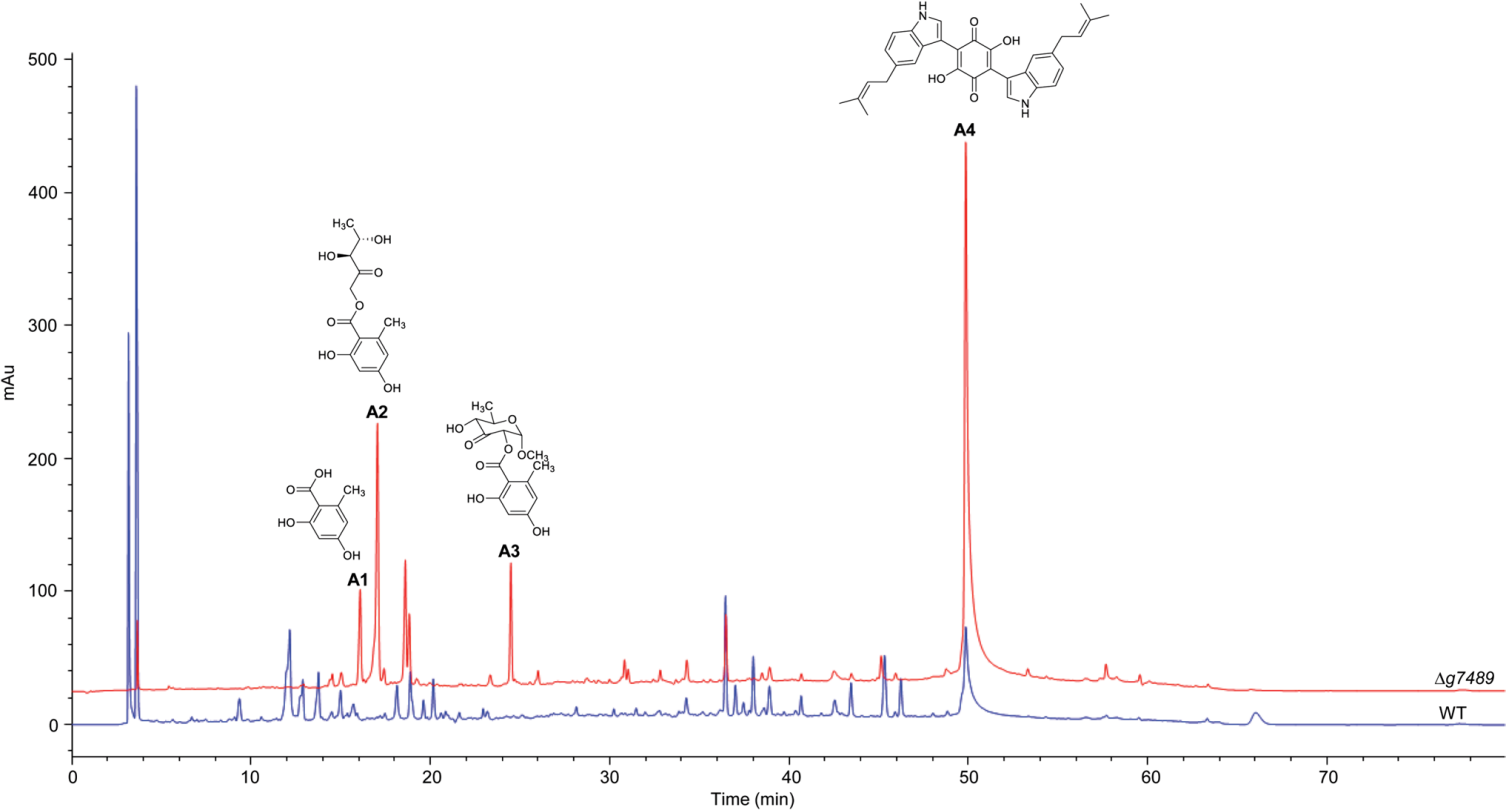
HPLC profiling for the WT and ∆*g7489* extracts, detected under UV absorption at 254 nm (blue line: WT; red line: ∆*g7489*)

To assess the impact of *g7489* deletion on the phenotype and growth of *C. olivaceum* SD-80A, we cultivated ∆*g7489*, WT, and ∆*g7489*-C strains on both PDA and sporulation medium. As illustrated in Fig. 1a, the mycelium of ∆*g7489* exhibited increased thickness and fluffiness, with clearly visible white mycelium masses. In contrast, little difference in mycelial growth and morphology was observed between WT and ∆*g7489*-C. WT and ∆*g7489*-C started to produce ascomata and spores in approximately 2 weeks, while ∆*g7489* was incapable of spore production (Fig. 1b and Fig. S3). The number of spores per Petri dish (90 × 15 mm) was approximately 1 × 10^10^ for WT and ∆*g7489*-C. These findings demonstrated that *g7489* plays a crucial role in spore formation, but it does not seem to be essential for vegetative growth. HPLC profiling of SMs from ∆*g7489* revealed significant alterations compared to the WT strain (Fig. 2). Notably, several novel peaks were observed in the HPLC profiling of mutant extracts, indicating that *g7489* significantly influenced the SM profile of *C. olivaceum* SD-80A.

Scale-up fermentation was conducted to characterize the differentially produced compounds between ∆*g7489* and WT. Compounds **1**–**4** (**A1**–**A4**) were determined as OA (**A1**) (Kloss and Clayton 1965), globosumone C (**A2**) (Bashyal et al. 2005), orsellide A (**A3**) (Schlörke and Zeeck 2006), and cochliodinol (**A4**) (Kingsland and Barrow 2009), by ESI–MS and NMR spectra (Fig. 2, Fig. S4-S15). **A1** is an aromatic polyketide. **A2** and **A3** are ester derivatives of **A1**. **A4** is an asterriquinone. Other peaks in the HPLC profiles were not identified due to their low concentration.

### Putative BGC of A4

**Fig. 3:**
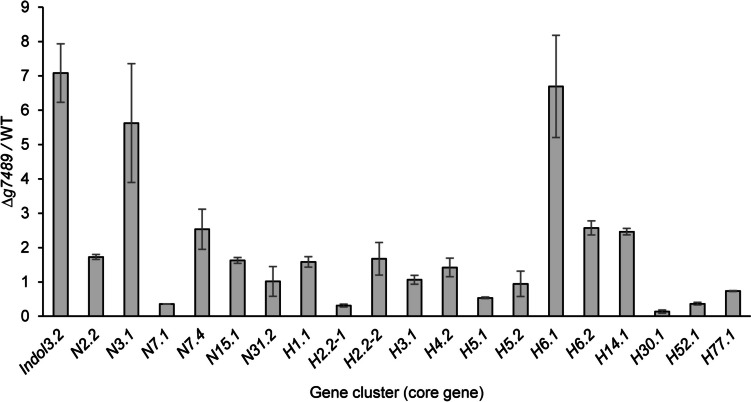
mRNA expression level of core genes from the selected BGCs. The core genes are denoted with the prefixes *Indol* (indole type cluster), *N* (NRPKS type cluster), and *H* (HRPKS type cluster), followed by cluster numbers

**A4** is biosynthesized by an indole-type cluster. Given that there was only one indole-type cluster (Table S1, cluster 3.2) in *C. olivaceum* SD-80A, we compared the cluster with the known cochliodinol BGC in *C. globosum* and found that cluster 3.2 exhibited high similarity to that in *C. globosum* (Fig. S16, Table S3) (Nakazawa et al. 2013). Most of the genes in the cochliodinol BGC have its homologous genes in cluster 3.2. The proposed core gene for cochliodinol biosynthesis, which encodes the indole prenyltransferase (encoded by *g1124*), exhibited 89.61% identity with CHGG_03684 from *C. globosum*. According to antiSMASH, 60% of the genes in cluster 3.2 exhibited similarity to the terrequinone A BGC. As expected, the mRNA expression level of *g1124* was upregulated 7.08-fold in ∆*g7489* compared to that in WT by real-time PCR (Fig. 3, *Indol3.2*).

### Putative BGCs of A1–A3

**A1** is formed through the condensation of acetyl-CoA and malonyl-CoA by PKS (Tao and Abe 2021). In fungi and bacteria, the biosynthesis of **A1** is mediated by non-reducing PKS (NRPKS) (Jørgensen et al. 2014; Sanchez et al. 2010). **A2** and **A3** are ester derivatives of **A1**. The biosynthesis of OA derivatives depends on key enzymes such as PKS for generating the polyketide moiety and esterase or cytochrome P450 for forming the ester bond. Previous studies have indeed reported that some PKSs also possess esterification functionality (Kealey et al. 2021). *C. olivaceum* SD-80A harbors 19 T1PKSs, encompassing 13 highly reducing PKSs (HRPKSs) and 6 NRPKSs. Among these PKSs, HRPKSs of clusters 1.1, 2.2, 4.2, 6.1, 6.2, and 14.1 (H1.1, H2.2–2, H4.2, H6.1, H6.2, and H14.1) and NRPKSs of clusters 2.2, 3.1, 7.4, and 15.1 (N2.2, N3.1, N7.4, and N15.1) were upregulated (Fig. 3). The domain organization of the PKSs is shown in Fig. S17 and Table S4. N2.2 (encoded by *g657*) and N15.1 (encoded by *g4635*) contain a starter-unit acetyltransferase (SAT), a ketosynthase (KS), an acyltransferase (AT), a product template (PT), one or two acyl carrier protein (ACP), and a thioesterase (TE) domain (Fig. S17), and their domain organization was the same with orsellinic acid synthases (OASs), which can produce OA, such as ArmB and OrsA (Lackner et al. 2013; Sanchez et al. 2010). Therefore, the two NRPKSs were regarded as potential candidates responsible for the biosynthesis of **A1**–**A3**. The mRNA expression levels of *g657* and *g4635* were upregulated by 1.73- and 1.63-fold, respectively, in ∆*g7489* compared to that in WT, which was consistent with the upregulated compound products. The gene organization of cluster 2.2 and 15.1 is shown in Fig. S18 and Table S5-S6. Cluster 2.2 contains a cytochrome P450, while there is no related post-modification enzyme in cluster 15.1.

Sequence analysis revealed that the N2.2 and N15.1 coding genes (*g657* and *g4635*) had open reading frames of 6601 bp and 6809 bp, encoding polypeptides of 2044 and 2147 amino acids with estimated molecular weights of 222.05 kDa and 235.24 kDa, respectively (deposited as GenBank accession numbers PP068241 and PP068240, Results S1–S4). For better visualization and classification, SSNs were constructed for approximately 1000 NRPKS sequences using N2.2 and N15.1 as queries (Fig. S19 and S20). NRPKSs from *Fusarium* and *Aspergillus* were the most common, with sequences predominantly grouped by genus. Despite N2.2 and N15.1 being from *Chaetomium*, only a limited number of sequences were identified from this genus, suggesting a need for further exploration of PKSs in *Chaetomium*. N2.2 can be clustered with the reported OASs ArmB and OpS1 (Feng et al. 2015; Lackner et al. 2013). Additionally, NRPKS TerA can be found in N2.2 and N15.1 SSNs (Zaehle et al. 2014). Phylogenetic analysis, based on complete amino acid sequences and KS domain sequences, revealed that N2.2 is closely related to enzymes involved in OA biosynthesis, particularly PKS14 and OpS1 (Fig. S21-S23, Table S7) (Feng et al. 2015; Jørgensen et al. 2014), while N15.1 is closely related to TerA (Fig. S21 and S22) (Zaehle et al. 2014). A comparison of the amino acid sequences showed that the overall sequence identity of N2.2 and PKS14 was 51.18% (N2.2 and Ops1, 35.15%; N15.1 and TerA, 41.18%), and the KS domain sequence identity of N2.2 and PKS14 was 74.65% (N2.2 and Ops1, 57.10%; N15.1 and TerA, 64.84%), whereas the TE domain sequence identity of N2.2 and PKS14 was 51.85% (N2.2 and Ops1, 50.00%; N15.1 and TerA, 38.24%).

**Fig. 4:**
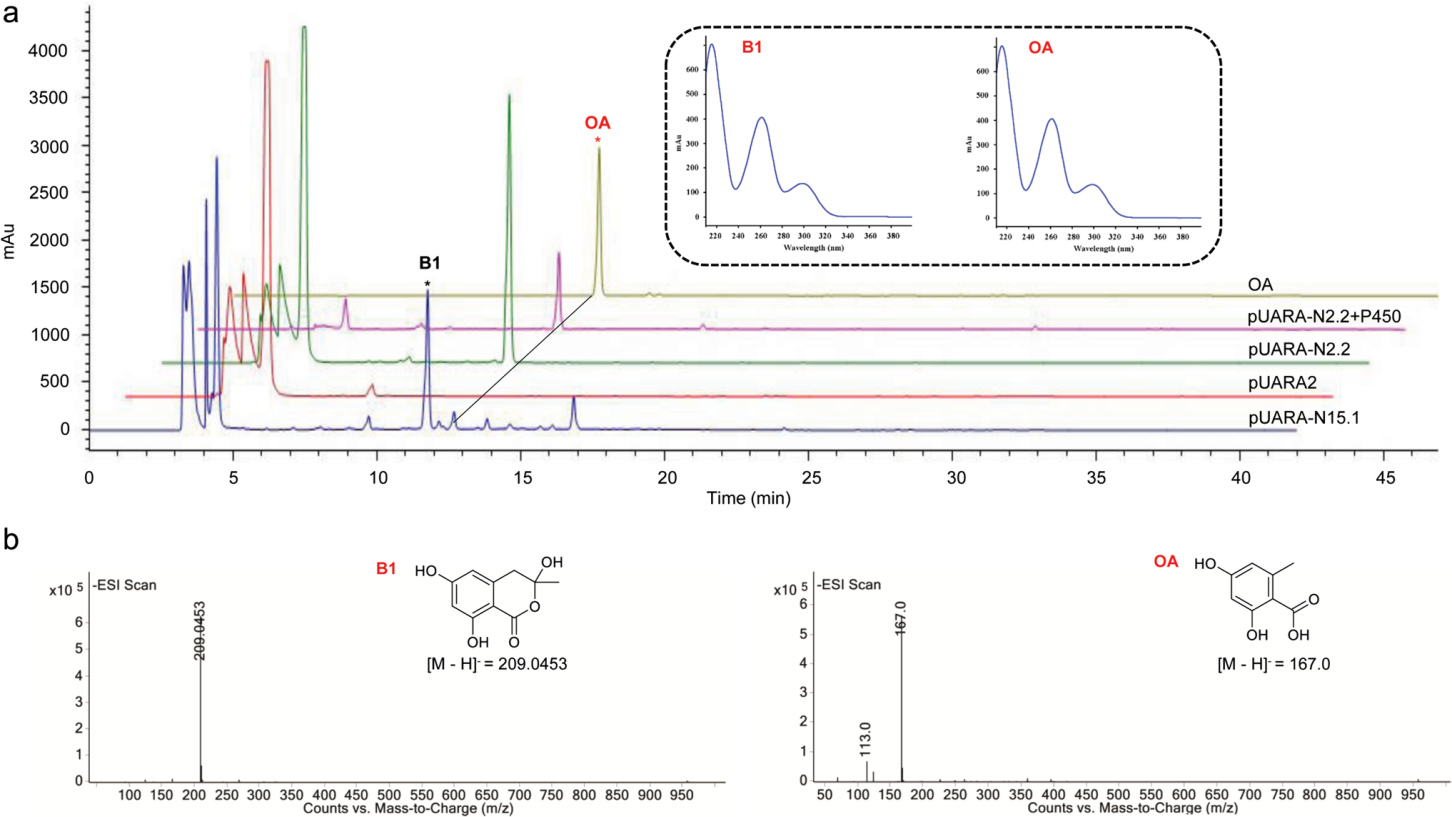
**a** HPLC profiling for the *A. oryzae* pUARA2, pUARA-N2.2, pUARA-N2.2 + P450, pUARA-N15.1 fermentation extracts and OA (detected under UV absorption at 254 nm). **b** Mass spectrum of new peaks of *A. oryzae* transformants

Heterologous expression of the complete gene sequences of N2.2 and N15.1 in *A. oryzae* (pUARA-N2.2 and pUARA-N15.1) was conducted, and HPLC analysis of fermentation extracts revealed the emergence of novel peaks (Fig. 4). The new peak in pUARA-N2.2 was determined to be OA (*m*/*z* 167.0 [M – H]^−^) by ESI–MS. One of the new peaks in pUARA-N15.1 was also OA. The other new peak (compound **B1**) was determined to be 3,6,8-trihydroxy-3-methyl-3,4-dihydroisocoumarin by HR-ESI–MS and NMR (Ariantari et al. 2020; Kameda et al. 1973) (Fig. S24-S26). Consequently, both N2.2 and N15.1 possess the capacity to catalyze the synthesis of OA, with N15.1 being a multifunctional enzyme. Notably, the evolutionary distance between the two proteins is relatively large. Cytochrome P450 in cluster 2.2 was heterologously expressed in pUARA-N2.2 transformant, and no OA derivatives were detected (Fig. 4), which may be due to the lack of deoxyhexose in *A. oryzae*.

## Discussion

The genus *Chaetomium* encompasses numerous species and is widely distributed, with its natural products exhibiting rich and diverse structures. Many SMs with biological activity have been discovered in *Chaetomium*, such as chaetoglobosins, depsidones, epipolythiodioxopiperazines, and azaphilones (Qi et al. 2020; Zhang et al. 2012; Zhao et al. 2022, 2021). However, despite the large number of BGCs predicted by genomic information in *Chaetomium*, only a small proportion of the compounds derived from these fungi and their specific BGCs have been identified. Therefore, thoroughly exploring the genetic and compound resources of *Chaetomium* is of great significance. As key molecules in eukaryotic gene expression, HDACs play a crucial role in regulating many fungal proteins and can influence SM BGCs (Pidroni et al. 2018). Using HDAC as a target to activate silent BGCs represents a crucial molecular epigenetic approach for mining active compounds in *Chaetomium.*

In our previous study, a *C. olivaceum* SD-80A strain with anti-MRSA activity (MIC: 0.78 μg/mL) was obtained, and four polyketides were identified in its WT strain, including three new compounds (Wang et al. 2020). However, bioinformatics analysis of *C. olivaceum* SD-80A revealed 46 BGCs, far exceeding the number of discovered compounds. By knocking out the class I HDAC gene *g7489*, four compounds (**A1**–**A4**) were obtained. **A4**, an asterriquinone, reached a yield of 385 mg/kg in the ∆*g7489* strain, 12.03 times that of the WT. **A4** was biosynthesized by an indole-type cluster, and by comparing with reported BGCs (Nakazawa et al. 2013), it was hypothesized that **A4** is synthesized by the upregulated cluster 3.2 in ∆*g7489*. **A4** exhibited antimicrobial activities against *Candida albicans* ATCC 10213, *S. aureus* ATCC 29213, and *Enterococcus faecalis*, as well as cytotoxic activity against the human cell lines KB (uterine cervical carcinoma), MDA-MB-435 (melanoma), and MRC5 (normal human lung fibroblasts) (Casella et al. 2013; Kingsland and Barrow 2009), suggesting that this compound could have clinical applications.

Moreover, a series of polyketides were obtained by knocking out the HDAC gene of *C. olivaceum* SD-80A. **A2** exhibited moderate acetylcholinesterase inhibitory activity with a half maximal inhibitory concentration (IC_50_) value of 7.34 μΜ (Xu et al. 2018). **A1** is an essential intermediate in pharmaceutical production. Over 200 OA derivatives with biological activities have been found in plants, lichens, fungi, and bacteria (Chen et al. 2020). For example, mycophenolic acid, derived from the *Penicillium brevicompactum*, is widely used as a first-line immunosuppressive drug (Zhang et al. 2019). Moreover, ascofuranone, which is derived from *Acremonium egyptiacum*, is a promising drug candidate against African trypanosomiasis (Araki et al. 2019). To investigate the biosynthesis of **A1**–**A3**, all PKSs in *C. olivaceum* SD-80A were characterized through bioinformatics analysis and RT-PCR experiments. OA is catalyzed by repetitive T1PKS in fungi and bacteria (Ahlert et al. 2002; Han et al. 2023; Sanchez et al. 2010; Weitnauer et al. 2001), whereas in plants, it is produced by T3PKS (Taura et al. 2016). Two PKSs, N2.2 and N15.1, capable of synthesizing OA were identified in *C. olivaceum* SD-80A, exhibiting a significant evolutionary distance and clustering with distinct PKSs in the phylogenetic tree (Fig. S21-S23). N15.1 clustered with TerA in the phylogenetic tree, sharing the same domain organization and a similarity sequence identity of 41.18%. TerA, found in *Aspergillus terreus*, can produce 4-hydroxy-6-methylpyranone (4-HMP), OA, and 2,3-dehydro-6-hydroxymellein (2,3-dehydro-6-HM), condensing acetyl-CoA with two, three, or four malonyl-CoA units (Zaehle et al. 2014). In contrast, N15.1 produced OA and 3,6,8-trihydroxy-3-methyl-3,4-dihydroisocoumarin (**B1**), another OA derivative. However, **B1** was not found in WT or ∆*g7489* of *C. olivaceum* SD-80A, which is probably due to its low yield or unknown post modification. N2.2 clustered with PKS14 from *Fusarium graminearum* and OpS1 from *Beauveria bassiana* in the phylogenetic tree and with OpS1 and ArmB from *Armillaria mellea* in SSN. The gene organization of cluster 2.2, cluster 15.1, and those of TerA, PKS14, and OpS1 showed significant differences, which may indicate that these BGCs branched out in the evolution of different species, resulting in different end products.

No OA esters were detected in *A. oryzae* despite the presence of N2.2 or N15.1. This absence could be attributed to the inability of both N2.2 and N15.1 to catalyze the formation of ester bonds in **A2** and **A3**. Additionally, the lack of deoxyhexose in *A. oryzae* and the strong substrate specificity of these enzymes might also contribute to this observation. Despite the co-expression of cytochrome P450 in cluster 2.2 with N2.2 in *A. oryzae*, no novel esterification products were generated. Schlörke and Zeeck reported that the sugar part of **A3** was derived from glucose (Schlörke and Zeeck 2006). Since no OA esters were obtained in *A. oryzae*, it was hypothesized that *C. olivaceum* SD-80A possesses specific enzymes capable of synthesizing deoxyhexose, which were apparently absent in *A. oryzae*. Deoxyhexoses serve as typical building blocks of bacterial SMs and play a crucial role in regulating their biological activities, particularly in the case of macrolides (Fernández et al. 1998; Schlörke and Zeeck 2006). These sugar moieties often contribute to the unique pharmacological properties and bioactivities of these compounds. While fungi generally do not produce SMs containing deoxyhexose, only a few fungal metabolites have been reported, such as sordarin and its derivatives (Weber et al. 2005). Bacteria typically employ enzymes like glucose-1-phosphate thymidylyltransferase (e.g., RmlA) and dTDP-d-glucose-4,6-dehydratase (e.g., RmlB) to catalyze the conversion of glucose-1-phosphate into dTDP-4-oxo-6-deoxy-d-glucose. Subsequently, enzymes such as dTDP-4-oxo-6-deoxy-d-glucose-3,4-oxoisomerase (e.g., QdtA) are utilized to further transform this intermediate into dTDP-3-oxo-6-deoxy-d-glucose, the possible sugar moiety of **A3** (Pföstl et al. 2008). Surprisingly, the orthologous protein of QdtA was not found in *C. olivaceum* SD-80A or *A. oryzae*, which means that it can produce deoxyhexose in *C. olivaceum* SD-80A by a catalytic mechanism that has not been described in fungi. The homologous BGCs of clusters 2.2 and 15.1 activated via the deletion of class I HDAC g7489 have not yet been found in other fungi. Cluster 2.2 contains up to 33 genes, including one NRPKS and two HRPKSs, and most genes have no predicted functions. In addition, the low substrate specificity of N15.1 is also relatively unique and warrants further investigation. Due to the high non-homologous recombination rate of *Chaetomium*, we have not yet obtained knockout strains of N2.2 and N15.1. It has been reported that in order to enhance homologous recombination efficiency, it is essential to knock out genes involved in non-homologous random recombination activity, such as LigD or Ku70 (Nakazawa et al. 2013); thus, it is necessary to develop a gene-editing system suitable for *Chaetomium*. Meanwhile, the heterologous expression in *A. oryzae* to verify gene function is a relatively efficient method for *Chaetomium*. Therefore, a more efficient way to exploit *Chaetomium* resources is to use global regulators such as HDAC to activate silent BGCs and then use heterologous expression to investigate targeted gene functions, mine new compounds, and deduce their biosynthetic pathway.

## Supplementary Information

Below is the link to the electronic supplementary material.

Supplementary file1 (PDF 3057 KB)

## Data Availability

All data generated or analyzed during this study are included in this published article (and its supplementary information file).
