# Supplementary material for: Activation of secondary metabolite gene clusters in Chaetomium olivaceum via the deletion of a histone deacetylase
Source: Appl Microbiol Biotechnol. 2024 May 11;108:332. doi: 10.1007/s00253-024-13173-8 (PMC11088548; doi:10.1007/s00253-024-13173-8)
Supplement: Supplementary file 1 [file 253_2024_13173_MOESM1_ESM.pdf]

Applied Microbiology and Biotechnology

Activation of secondary metabolite gene clusters in *Chaetomium olivaceum* via the deletion of a class I histone deacetylase

Peipei Zhao[1], Shengling Cao[1], Jiahui Wang[1], Jiaying Lin[1], Yunzeng Zhang[2], Chengwei Liu[3], Hairong Liu[1], Qingqing Zhang[1], Mengmeng Wang[1], Yiwei Meng[1], Xin Yin[1], Jun Qi[1], Lixin Zhang[1,4], Xuekui Xia[1]

[1]Biology Institute, Qilu University of Technology (Shandong Academy of Sciences), Jinan 250103, Shandong, China

[2]Department of Thoracic Surgery, Shandong Public Health Clinical Center, Jinan 250013, Shandong, China

[3]Key Laboratory for Enzyme and Enzyme-Like Material Engineering of Heilongjiang, College of Life Science, Northeast Forestry University, Harbin 150040, Heilongjiang, China.

[4]State Key Laboratory of Bioreactor Engineering, School of Biotechnology, East China University of Science and Technology (ECUST), Shanghai 200237, China

Peipei Zhao and Shengling Cao contributed equally to this work.

Xuekui Xia: xiaxk@sdas.org; Tel: 86-531-82605355; Fax: 86-531-82605355.

Lixin Zhang: lxzhang@ecust.edu.cn; Tel & Fax: +86-021-64252575.

Jun Qi: qijun@sdas.org; Tel: +86-531-68606197; Fax: 86-531-82605355.

Supplementary Information

Contents

Result S1 Gene sequence of *g657* for heterologous expression in *A. oryzae*……………………………4

Result S2 Gene sequence of *g4635* for heterologous expression in *A. oryzae*……………………………8

Result S3 Predicted amino acid sequence of *g657*…………………………………………………12

Result S4 Predicted amino acid sequence of *g4635*…………………………………………………14

Table S1 Primers used in this study……………………………………………………………16

Table S2 Gene clusters predicted by antiSMASH…………………………………………………17

Table S3 Predicted gene functions of cluster 3.2……………………………………………………20

Table S4 Domain organization and protein homologues of HRPKSs in *C. olivaceum* SD-80A…20

Table S5 Predicted gene functions of cluster 2.2……………………………………………………21

Table S6 Predicted gene functions of cluster 15.1……………………………………………………21

Table S7 Fungal PKSs used to create the phylogenetic tree………………………………………22

Fig. S1 Domain prediction of Hos2 orthologs and phylogenetic analysis ……………………………23

Fig. S2 Schematic representation of gene disruption strategy and electropherogram of PCR products..24

Fig. S3 Micrographs of Δg*7489* and WT……………………………………………………………25

Fig. S4 Mass spectrum of compound **A1**……………………………………………………………25

Fig. S5 ^{1}H NMR spectrum of compound **A1**……………………………………………………25

Fig. S6 ^{13}C NMR spectrum of compound **A1**……………………………………………………26

Fig. S7 Mass spectrum of compound **A2**……………………………………………………………26

Fig. S8 ^{1}H NMR spectrum of compound **A2**……………………………………………………27

Fig. S9 ^{13}C NMR spectrum of compound **A2** ……………………………………………………27

Fig. S10 Mass spectrum of compound **A3**…………………………………………………………28

Fig. S11 ^{1}H NMR spectrum of compound **A3**……………………………………………………28

Fig. S12 ^{13}C NMR spectrum of compound **A3**……………………………………………………29

Fig. S13 Mass spectrum of compound **A4.** …………………………………………………………29

Fig. S14 ^{1}H NMR spectrum of compound **A4**……………………………………………………30

Fig. S15 ^{13}C NMR spectrum of compound **A4** ……………………………………………………30

Fig. S16 Comparison of cluster 3.2 and cochliodinol BGC from *C. globosum*……..……………...…..**31**

Fig. S17 Domain organization of NRPKSs of *C. olivaceum* SD-80A………….. ………..…..……..…..**31**

Fig. S18 Gene organization of cluster 2.2 and 15.1. ……………………………. ……………………**32**

Fig. S19 SSNs network analysis based on N2.2 and its homologous sequences……………………..……**32**

Fig. S20 SSNs network analysis based on N15.1 and its homologous sequences……………………...**33**

Fig. S21 Phylogenetic analysis of fungal NRPKS sequences ……………………...……. ……. ...……**34**

Fig. S22 Phylogenetic analysis of fungal KS domain sequences. ……………….……....……...……..…**34**

Fig. S23 Phylogenetic analysis of fungal TE domain sequences.………………………. …..……………**35**

Fig. S24 Mass spectrum of compound **B1**……………………………. …. …………………………**35**

Fig. S25 ^{1}H NMR spectrum of compound **B1**…………………….…………………….……………..**36**

Fig. S26 ^{13}C NMR spectrum of compound **B1**…………………...……………………...………..**36**

Result S1 Gene sequence of *g657* for heterologous expression in *A. oryzae* (red: exon; blue: intron)

ATGCCTGCCTCGGGAGAACAACATCGATTCGTATTGTTCGGTGGTCAGGGGTCGCCCACAG
TTTTCTCACATACCACGGCGGCGACGGCAGAGGAAGATGTTAACTCATCAAGCGCCTGTAG
TATCCTCCTATCGAAATGTCATTCGGCATTCTTGGAAGACATCGCTAGCTTAGACGACCGCTC
GCAAAATCTGCTCGCCATTGACAGCTCCCGGTTTTCCTGTCCGGGTCATCTTCTAAAACCGC
CAGCACTATATCATACACATGCCGTTATCCAAGCGACGACTCTCTACCTTTGCCAGGTTCTGC
ACTATCTTGCCGAGACTCTACGGCAGTACCCGAACCAAAGCTTTCAAGAGGTGTTTGATGG
GCTTCAGGAGACTGCCGGTTTCTCCTCGGGCCTCCTTCCGGCTACGGTGGTCGCCCGTTCC
CGTAGCGTGGAGGTTTTCTTGGCGAACGGAGTTCGAGGCTTCCGCTTTGCGTTTTGGATTG
CGTATCACAGCAGAGCATGGAGCCGTGAAGCTGAAGCGAGAGCAGATGACGTCGCTTCTG
GTCATGATCACAGTCCTGAGACTACATGCTCCCTGGTGATCCGTGGTCTTACCCCTGGCCAG
GTGGAAGAAAGGTTATGCCGGTACGCGGCTACCAAGAACGAGCGAAATGGATCAATTTCTA
CTCAATCCCGACAGTTGCAGATATCGGCCATATCCAGCCAGACTGTAGTGTCAGTGTCGGGC
CCTAGAGGGGAGCTGTCCAATTTCAGGGCACATGCAGTCACAGATGTGATCACAGCATTTG
CGCATATACATGGATGGTACCATGGCGGAGACCGACTCGAGGCCGCCGTCCCCCTAGTTCTC
GAGGATTTACAGGATCGGGGTGCGACTTTTCCCCCTTGTCCCCATCATTGCAAGTCGATGCG
CTCGACACAGGACGGAGCGCTTTACGTTGATTCCGACACCGAGTCTCTAGGGCTTTCCGAA
TGGTTGGTACGCCATCTGTTGGTGCACTGCGTGGACTGGAACAGAACCGCCAGTAAGATCA
CGGAAAGTGTCAAGACTCTTTTGGGACTGGACGCGGTTACAAGCGTTAAGGTGCTCTCTTT
TGGTCCAAGTTCCGGTACGCTGCTGGCAGGATTACAGCCCGGCAATTCCAGAATCAAAGTA
CTGGACGTCTCACCTTTTCGGGCAGGCAACAGGACTTCGTTACCTAACAGCCATGACGACG
ACATAGCTATTGTAGGAATGAGCGTCAATCTCCCCCGAGGAAGGGGTACCGAGGAGCTTTG
GAAAACGCTTTCAGATGGTCTAAGTGCTGTCCAAACGATCCCGGAGTCCCGTTTCAAGGTT
TCCGACTATTACTCAACAGAGAAAGATGCGAAGTCGCGGTCCATGCCAGTAAAGCACGGCG
CGTTTCTGGCAGATCCTTTCTGGTAAGTGGCATTGTCTCACGGCTTTCGAGTTACGTACATG
CTAATGCGGAACTTTGTGTATCTTAGTTTTGACAACGCCTTTTTCAATATTTCCCCGCGGGAG
GCGAAGTCAATGGACCCGCAGCAGCGGCTACTGCTACACGCAGCTCAGGAAGCACTGGAA
GATGCTGGATACGTAGCAGACTCAACGCCATCCTTCCAAAGAGCGTGTACCGGATGCTACAT
CGGCGTTGCTACGGGCGACTACGCCGACAATCTGCGCGCCAACATCGACGTCTTCTATGCC

CCCAGCACGCTACGCGCATTTAATGGTGGGAGAATATCATACTTCTACAAGTTCAGCGGCCC
TACCATCGTATCGGATACGGCATGTTCATCTTCCTTGGTCTCCGTGTACCAGGCTTGTCGGGC
TTTACAGCAGGGCGACTGCACGGCAGCCATTGCTGGTGGCGTGAATGTCATCTCGAGCCCA
GATGTCAGTTTTATATCCATCGCATAATGGAGTCTAAAGCCGGTAGTGGATATATTGCTGACA
GAATCAATTTCTCCCTTACTAGATTTACCTTGGACTAGCTAAAGGCCACTTTCTCAGCCCCA
CGGGGGGATGCAAATCCTTTGACGCCGCCGCCGATGGATATTGTCGGGCCGAAGGGTGTGT
CCTTTTTGTGCTCAAACGACTGTCGGCTGCCGTTGCCGAGAACGACCGTATCCACGGGGTC
ATTCGCAACGTGGTGGTTAACCAGAGCGGTAATGCTCGTTCCATCACGCATCCCCACAGCC
AGACACAAATCGATCTGTTCCATCGACTGATGAAGCAGGTCAATATTGAGCCTGGATCCATT
GGCGTTGTGGAAGCCCACGGGACAGGCACACAGGTTGCTATCCCTTAACTATCTCATGAAG
ATGAATTCGATTGCTGACTGCGCGACCTATTCCTCACAGGTCGGAGACGCAAGCGAAATGG
AAAGCCTGAAAGCCGTTTTTGGTCCGCACCACTCCCTCGCCAATCCTCTCGTTATCAGCTCC
ATCAAGAGCAACATCGGCCACTCTGAAGCCGCGTCTGGGGCGGCGGGTCTGGCAAAGCTC
CTCCTTATGCTTCGCCACCACAAGATACCCGTGCAAGCCGGTCTTCGAAACATCAACCCGC
GCTTTGCCGACATGGCAAGCGCTGGCTTGGTTACACCGAGCGAGACAGCAGAATGGAGCC
ATGCACATAAGACTCCGAGGAGGGCCCTGCTGAACAACTTTGGGGCTTCCGGCTCCAACGC
GTCTCTTCTCCTTGAAGAGTGGGCGGCCGAGCCCACGAAGGGCCAGAATATCAACAAACT
GCCCGAGCGTTCGGCCTATGTGTTTGCGCTTTCGACGAAATCTCCAAAGGCATTAGAGAGA
GCGATAGAACAACACATCCAGTACTTAAGAGACTCCCCATCTTCGACGACGTCTGTCGAAG
ACATCTGCTACACTGCCGTCGCGCGACGCCAGATCCACGAATACCGCATTTCCGTGACGTG
CACCTCGGTGAACGATCTGCGCGCGAAGCTAGAATCTCTTGGAAGCCTCAGAACTATGGGA
ACCATGGTCCCTGCTCGTCGCATTTCAAGATCTGTTTTCCTCTTTTCGGGGCAAGGCAGCCT
ATACGAAGGCATGGGACGGGAGCTAATGAGCACACTGCCACAGTTCAGGGACGTCATTGC
GAAATGCGACACAGTCCTTCAGGGACTAGGATACCCAAGCATCCTAAGCTTTTTTTCGGGG
CCTTCTGGACATGCAGGAGAAATATTGCCTGAGAAAGACCATGTCATTTCTTCGCAATGTGC
CTGTGTCGCTTTGGAATATGCACTTGCCGTGGTTTTCGCCTCGTGGGGAATTGTACCCCAGT
ACGTCATGGGTCATAGGTATGTAACATGCACGACTCAGAGGAAACGATCAACTTTACTCACT
CACCACATCATCTTCGCAGTCTCGGCGAATATGTGGCCTTGTGTTTTTCGGGTGTGTTGACA
TTGGAGGATACGTTGCACGTGGTAGCCGCGCGAGCCACCATGATGACACAACAGTGTGCTC

CTAATTCTACCGGAATGATGGCATGCAGCCTGTCCTCCGACGGAACCAATGAATTGATATCG

ACAACCATTGGAGATGTCCCCGAACTAACCGTGGCCTGCATCAACGGTATCAACGACTGCG

TGGTTGGTGGCCCGTTGGGCCAACTGGAAAGATTCCAAACACATTGCCAGGCAATGAAACT

CAAAACCAAGGTACTCGAAACTCCCTTCGCATTCCACACCTCAGCGATGGATCCCATTTTG

GAGCCCCTGAAAGCCTTGGGAAGTTCTATCAAGCTCTCACGACCCACCACCCCTATCATCTC

TACTGTCTTTGGACGGCTGTTGGGAGACGACGACCTCTCCAGCGATTATTTTGCAGCGCATG

CCCGACAACCCGTCCTATTCACTCAAAGTCTTTCGTCCATGAAAGCTTCAAAAGCGGCCGA

TGATGCTCTCTTTCTTGAGCTTGGCCCTCACCCCACACTGTTACCCATGGTTGAAACCTTCT

TCGGTACGGATATTTCGCATGCCTGCTTGGGGACGCTACGAAAGGGGCAAAGCGCTTGGGT

CTCTCTCAGCCGTATGCTGTCTGAGATTTACGTACTAGATAGCCCTGTAATCTGGCGCCAGAT

CTTCTCGCCAACATCGGCAAAGCTGGTAAGCCTACCGGGACACCCGTTAGAGGGCTCGACA

TACTTCACTTCACGCCGCGAAGATCGTGGGCCTCAAGCATCCGTTCCCCTGGACGAAGATA

CATCATCCACGCGCCGCCATACTAAGACTGGGCTCACCCTCCTACCCTGGATGAAGAGTATA

GATCAATCCAAAGAAACCTGCTTGCTGGAAACCTCACTGGAAGTTATCGGGCCTTTGATCC

TGGGCCACGACGTCGGCGGGACCCCCATCTGCCCAGCGTCAGTATTTCACGAGCTCGCTCT

CGAGGGAGCATCCATGCTGCTCGAGCCACCCGATGGATATTTGATGGTCGTCAGCGGCTTG

AACATGACCAGCCCCTTGGTGTATGAGCAATCCCGTGAAGTTGATGTGGTGACAGTGCACA

TCACAAAGCACAGTCTCGGCTCGGGGGCAGATTTCAGGATATCCTCGTGCTTTGACGCAGA

TCTTACAGAAAAGGTCCACTGCACCGGTACTATTATTCTCACCGATTTCCGCACGGTCGAAT

TTCGTTGGTCAAGGGACGCTGCAATGGTGACGAGGCAGAGCCGCTATCTCAATGGCCCGGG

AAAAGATGACAGCAGCATCTTCCGTACCAAAGTGCTCTACCAGGCGGTCTTTCCGCGCGTC

GTCCGCTATTCTCCAGAGTACCAGAGCTTGTTGCATTTGGCGGTAGCCGATGGCAACCTCGA

AGGCATCGGGTTGCTCAGAATGCCACTCGGCACCCAGACCCGCGGTTACCTTGCGCCCCCT

GTTTTCACGGACACGCTTCTACACGCGGCGGGGTTCATCGCCAATCTTGGGCTCCGGTCCG

ACGAGGTGGGGATCTGCGCGCGCGTTGAGTCCATGGAGATATCACACAGGGAGATCGATTA

CCTGGACACCTTTACGGTGTACTGCAGCTTACTCGAGGTGAAAGGAGCTATTCTGGCCGAC

ACGGTTGCCCTGGACAGTGCGGGAAAGGTGGTCGCCGTCGTCCGTGGTATGGAGTTCAAG

AGACTTCGACTTCATACTTTCCAGCATCTTTTATCCCAAAGACTCTCTGCATCGGATCCTTGG

GAATTTCCCAGGAAGGTCTCGGCGGAGACCAGTGGTAGCGAAACCATCAACACTCCGCCG

ACCGAGGCCTCCGATATTAGCAGCAGTATTAGCCACGACGCTGTCAGAGCATCGCTCAAGA

ATCTTGTTAAGGAACTTGGTGGTTTCAATGAACGAGAGCTGGATTACGAAAAGCCGCTGGA

ACAGCTGGGAATTGATTCGCTCATGCTGATCGAGATCATCGCGAAGCTGGCGCATTCTTTCC

CGTGTCTCGCGGGGTCATCACGTATGGCCCTGTCTGAGTGCGCGACCCTCGCATCACTCGA

ACACATGTTACTCGAATCCATCATGGGAGTTTCGGAGGCTCGCCCCCTTCATCAGCCTACCC

TCACGCACACAACCGCGCCATCCAAGCCCCAGAAAGATCCAGGTTGGGGAAATGCACTAC

GAATTCCATTGATGCGAGGCCAGAGATCTTCAGCAAATGGTGGAATGCAGGCGCACAACAA

CTTGGTAGCCCTCCACTTGTCACCTGCCGATATGTCTTCGCCACTTTGTTTGATCCATGACGG

GAGTGGTCAGGTCAGCATGTATGCACGACTGCGTGGCCACGACCGCAGCACCTATGCAATC

TTTGACCCGCACTTTGGAAGTGACAACATCTCGTTCCAGAGCCTATCGCACATGGCGGAGG

AGTACACCTCGCATCTTACGCAATCACGCCCTAATACAGCCATCATATTAGGCGGTAAGCTTT

TCTTTTTCTTTCTTTTTGCATGAGTATCATTGCTTACCCGGAGCTGAGCTGACCTTGGATCTA

GGATGGTCTTTTGGCGGAATCGCGGCCTTTGAGATGGTCCGACAGCTCATGGCCAAGGGGT

TCGATGTGAAAGGTCTCGTGTTGGTCGACTCTCCTAGTCCAATTGACCATCAGCCTCTCCCA

GACGACGTCATTTCTTACGCCATTGGCCTCAGTGATCAGAAGGTTGCGTCGGTTGTCAAAA

GCCATGATGCGATAAAAAACGAATTCCAGTCCAACGCCTCGCTGCTGGGTAGTTACGAGGC

GGTACCTCTACCTCGGCAAACCGGCCGACGAAGTCTGCCTACCGTCATGTTAAGAAGCCAG

CGTGTTCTTGATACCGAAAGCCTCTGCGGTGTCAGGTACGACTGGCTCAGTAAGCAGAATG

CTCGTGATGCTAGCATCGTAGACTGGGAAGGGTTAATCGGCGGCCACATTGAAGTTTTACCG

ATCCCTGGGAACCATTTCGAGGTTTTCTCGCCGGAAAATGTAAGGAAAACACTCTGTGCCT

AACGAAACGAACTCATATACTGACTGATTTCTAGATTGGCAAAACGAGTGCTCAGGTTTGG

AAGGCGTGTCGTTACATTGAGAAGGCAATCAATTAG

Result S2 Gene sequence of *g4635* for heterologous expression in *A. oryzae* (red: exon; blue: intron)

ATGGAGGCCAAACTCGACGAAAGCTGGGCTGAGCGACCGGCATTCCTCCTCTTTGGGGATC
AGTCACTCGATAGCCATTCCTTTCTCGCTCAATTCTACCGCCAATCGAAACACGGCGAGCTA
GCAAGGGTCTTCTTGCAGCAGGCGAACCACGCTCTGGTGGGTGTTGTCGAAAGGCTACCT
GCTCTGGAGCGAGCAACACTCCCCGATTTCAGGACGCTGCGGCAGCTCAACGAACGATATC
ACAATGCGCCACTGAAGCACTCGGGCATTGATGCGGCGTTGTTGACAATATCGCAAATTGC
GCATTACCTCGAGTGAGTCTGTCTGCCTTGGCTAAGCACACCGCTCTTCCTCGGCTGTTGTT
GTTTTGTCTCGTCTTCTCTGACCACGGCACTCTAGTCACGCTGAAAAGCACTGTGGTGATAT
CACACAGCCTCATAATACCCTCCTCATCGGGCTTTGCTCTGGGCTTTGGGCTGCATCCGCTAT
CTCGGTAGCGCCCTCGCTCCCAGACCTCGTCCATGTCGGCGTTCAAACAGTTCTCTTGGCTT
TCAAGACGGGTTGCTACGTTCACGCCATCGGACAACGGCTGAACCCTGCGTTTGAGCGTTC
TGAGAGCTGGAGCTACATCTTCTCAGGGACAAGCTTTGAGGATGTCACCGGAAAGTTGGAT
GCTTTTCACGATGCCTCGGGTGAGCAGCCCGCAGCCTGTCGTGCCTCGCTGATCCACATAGCT
AACACGCTGTTTAATAGAACCTTTCTCCAGCCAGCCGCGCATATATTAGTGCGGTATCCGATA
ATAGTACCGCGATATCTGGCCCACCCAGTACACTAGATGCCATAGTGAACAACAGGATTTTA
TCCGCTGACCCGATCGCCATCCCCGTTCATGGTCCCTACCACGCGCCACATCTGCATTCCAC
CGCAGACATCGAGAGGATTCTAGAACTTGACAAGCCAGAGACAAAAGATGGCTTGTATAA
GACGCGACCGCGATCGCCCATCATGGACTGCTCAACCGGGACTTGGTACTCGCCCCCGGAC
ACAAAATCGCTCCTGATCTCGGTCGCCTCTACCATCTTGAACAGGCCGTTGATGTTTAACAA
AGTCCTCAACGGTTGCGTTGAGGCTGCTCGCCTATTTCAAGACAATCGGTGTCTCGTACTCC
CTCTTGGTCCAACCCAAAATTCACTAACGCTTCAGAGACGCCTCCAGCAAGAAGCTGGATT
GGACATCACTCTTCGCATGCCGCCCTCTACCCCATCGGATGCAACATCATCCAAGATAGGGA
ACCACGGCTCGAGCGGGAGACCCAAGCTTGCCATCGTCGGCATGGCCGGCCGGTTCCCTG
ACGCCGCCAGTCACGAAGCGCTGTGGAAACTGCTCGAAAGTGGCCTCGCTGTCCACCGCG
AGGTGCCACCGGATCGTTTCAACGTCAAGACGCACGTTGATCCTTCGGGCAAAGGGAAGA
ATATGAGCCACACTCCATACGGCTGCTGGATCAAGGACCCGGGTCTGTTTGACCACCGTGT
CTTCAACATGTCGCCGCGCGAGGCCCGCAACACAGACCCGATGCAGCGGATGGCCTTGAC
CACTGCGTACGAGGCTCTGGAGATGTCAGGTTACGTTCCCAACAGGACGCCGTCCACAAG
GCTTGATCGGATTGGCACCTTCTATGGCCAAACCTCGGACGACTGGCGCGAGATAAATGCC

GCCCAGGACGTGGACACGTACTTCATCACGGGAGGCGTCCGCGCCTTTGGGCCTGGCCGC

ATCAACTACCACTTTGGCTTCAGCGGGCCGAGTCTCAACATTGATACCGCTTGCTCCTCCAG

CGCGGCTGCCATGCAGGTGGCATGCTCGGCGCTCTGGGCCCGCGACTGCGCGACACGGCC

ATTGTCGGTGGTCTCTCGTGCATGACCAACCCGGACATCTTTGCCGGGCTCAGTAAAGGCC

AGTTTCTGTCAAAGAAGGGCCCATGCGCCACCTTTGACAACGATGCCGATGGGTACTGCCG

CGGTGATGGCTGTGCATCCGTCGTCGTCAAGCGTCTCGATGACGCCTTGGCCGACCAGGAC

AGGGTTCTCGCTGTCATCCTCGGTACCGCAACCAACCACTCGGCGGATGCTATCTCCATCAC

GCATCCTCACGGGCCGACGCAGTCTACCCTGTCTACCGCCATCCTTGACGACGCTGGAGTT

GATCCCCATGATGTTGACTATGTGGAGATGCACGGCACCGGCACCCAAGCCGGAGATGGCA

CCGAGATGAAGTCGGTCACCGACGTCTTTGCACCCGCAGACCGGCCAAGGCCCGAGGACA

GGCCGCTTTTTCTAGGAGCAGTCAAAGCAAACGTCGGGCACGGCGAGGCCGCTTCCGGAG

TCACAGCGCTCATCAAGGTACTCTTGATGCTTGAGAAGAACACTATCCCGCCCCACGTAGG

GATCCAGAAGAACGGCGGGGTGATAAACAAGACGTTTCCTAAGGATTTTGCAGCGCGGAAT

GTCAACATCGCATTCCAACCAGTTCCCTTCAGAAGGAGGGATGGCAAGCCCAGGCGCGTCT

TCGTCAACAACTTCAGCGCCGCGGGTGGTAACACTGGCCTCCTCGTCGAGGACCCCCCGA

CAGTTCCACCAGCGAAGCCGGATCCTCGCACCCACCACGTCGTCACTTTGTCAGGGCGAGT

CTGGGAGTCTGTGAAGGGAAATGCTGAACGTCTTCTTGAGTGGACGGAGCGGAACCGCGA

TACACCGCTCTCGCACATTTCCTATAGCACAACAGCAAGAAAGCTACACCACGTCTGCCGT

ATGAGCGTGACGGCCAAGGAAATTGGAGATTTACAGGCAGCCCTCAGAGAACGCCTCCGG

GATCTGGACCTGAATCAAGCTATCCCGGCTCCCCATCACCCTAAGGTGGTCATGATGTTCAC

AGGGCAAGGGTCGCAATACGCCGCAATGGGGAGGGAGTTCTACGAACATTACGCAGTGTT

CCGCGAGAGCATCGACGGCTTCATCGACCTCGCCCGCCTGCAAGGCTTCCCGTCCTTTCTC

CCTCTCATTGATGGCACCGACCCAAACTTGGCCGAGATGTCACCCATTGTGTTGCAGCTCG

GCTTGGCATGCTTCGAGATGGCCCTCGCCCGCCTATGGGGTTCCTGGGGAATCAAACCCGC

TGCCGTCGTGGGCCACAGCCTGGGAGAGTATGCCGCTCTCGAAGTAGCCGGCGTGCTCTCG

GCTAGCGATGTCATCTACCTAGTCGGTTCCCGTGCCAAGTTACTCGTCGAAAAATGCAAACC

TGGCAGCCACGGTATGGTCGCCGTCCAGGCCCCGGTCCAAACGGTCCTAGAGCTGATGGGC

ACCGAAGCTGGTGGCCTCAACATTGCATGCATCAACAGCCCCCGCGAGACCGTCATCAGTG

GCGACTGTGAAAAGTCAAAGGAGATGGCCACGCATATGTCCGACCAGGGTTACAAGTCCA

ACCACCTGCATGTGCCCTTCGCTTTCCACTCCCCCCAAGTGGAAGTCATTTTGGATGATTTT
GAGAAACTCGCAGAGGGCGTTAGCTACAATGCCCCCAAGATCCCCATCATCTCCACGGTCC
ATGGAAATGTCATCGAGGGCAAGTCGATCGATGCTGTGTATCTGCGCAAACACGCACGCGA
TACAGTCCACTTCCTCGACGGGCTTATCGAAGCCCAGAAGTTGGGCACTATCGATGACAAG
ACTGTTTGGCTCGAGATAGGTCCCCACCCGGTCCTTTCGAACATGGTCAAGGCTACGTTTG
GAGCCAGCACGTTAGCGGTTCCTACACTGCGCCGTACCGAGACGTGCTACAAGACGTTGAC
GGGCACGCTCGCCACTCTGCACAACGCACATCTCAAGATCGACTTCAACGAGTATCACCGC
GACTTTGCCGACTCAGTGCGTCTCTTGAATTTGCCCGCGTATTCCTTCAACGATAAGAACTA
CTGGATTCAGTACACGGGCGATTGGTGTCTCACGAAGCACAACCTGTCGGTCGCTGCAGCG
GAACAAAAGCCTGCAACTCCTTGGGTCCCCACGACGACAGTACACAAGCTCAACAGAGAA
ATTGTCGAGGGCGGCGTGGCGATTGTTGAGACCGAGTCCGAGCTCCACCAAGAGCAGCTT
CGAAACGTGGTTTGTGGCCACCAGGTCAACGGCGCCCCCTGTGCCCGTCATCACTGTACGG
CGACATGGCCATGACCGTGTGCGACTATGCCTACAAGCTTCTGCGGCCTCAGTCAAAGGGT
ATCGGCTGTAATGTTGCGGATATGCAGGTCTTCAAGCCGCTCATCTTCGACGACAAAGCCAA
GAGTCACATCCTGCGGTTGACAGTCACCGCTAATGCCGAGGCTGGCGAAGCTGACCTGGTC
TTCCACACGGCTCATGGTGGCAAGAAGGTCGAGCATGCCCATTGCAAGGTCTACTACGGCA
ATCATGACGAGTGGCAGGACGAGTTTGACCGGGCCTCGTACCTTATCAAGTCCCGTGTCGA
CTTCCTTATGGAGGCAGAGAAACGTGGTGCTGCGTCCAAGATTGGCCGCGGCTTGGCGTAC
AAACTCTTTTCAGCGTTGGTTGACTACAACGCACGCTACCGTGGCATGGAGGAGGTCATCC
TTGATAGCACTACCTGCGAAGCGACGGCGAAGATCCGCTTCCAGACCACAGATCAGGACG
GCAACTTTTACTTCAGCCCCTACCATATCGACAGCTCCTGCCATATCTCTGGCTTCATCATCA
ACGGCACCGACGCTGTGGATTCACGCGAACGAGTCTTCATCTCCCACGGCTGGGGTTCCAT
GCGATTTACCGAGATCCCGAATGCAAACAAGGAGTACCGCAGTTACATCCGCATGCAGCCG
GTGAAGGGTACCGAGATGATGGCTGGCGATGCCTACATCTTCGATGGCGACAAGATTATCG
GCATGACGGGGCGCATTAAGTTTCAAGCCATCAAGCGCCACACGCTCAACATGATGCTACC
TCCGCGGGGAGGTCAGGTGGTCTCGCGCCCAGCCCCTTCGACGACCAAAGCGGCCCCTTC
GAAGAAAAACAAGGAGACGGTGAACCCCTCCAACATTAACAAGGTGAACCAGAAGCTTA
ATAGCGTGACGGCCGCAGTCATGGACATACTTGTCAGAGAAATAGGCTGCAGCCATGGGGA
GCTCGTGGACGACGCCTCGTTTGACAATCTTGGCGCTGATTCCCTAATGGCTCTACAAGTAT

CGTCAAAGATACGCGAAGAGTTGGAGCTCGACATTGAAGCGCAAGCCTGGCTCGACTACC

CTACCATTGGCGCCTTCAAAGCCTACCTGGCGACCTTTGAGAAACCGGGTCGCAAGGAAA

GGGTGCCATCTAGAGGGTCTGCAAGGACAACCGACGACGAGTCCCGCGACGAAGAATCTG

ACTCCGACGTCACGACACCGCCCGAAGAGAGTGACACACATTCTATTAAGGGAGGTATGCA

GGATGACGCCGAGCCAGGCGACTCTGCCCAGAACCAGAAACTTCGAACCATCATCCGCGA

GTCCATCGCCACGGAAGCGGGTGTCGACGTGCAGGAAGTCATTAGCGCGACGGATTGGCC

GAGTCTTGGTGTTGATTCCCTCTTGGGCCTGGGAATCAGCAGCCGAATTCGTGAGCTAGCT

GGTATAGAGGTGCCCACTGACCTCTTCCTTGAGCACCCAACGCTCAAAGATGTGGAGCGCG

TCTTGGGCGTCACCAGCCCCCCCAAGAAACCTGCCGCTCGCGAACGGCAGAACCCCAAGG

AAAAGAGCAAAGCGCACACCGCTGCCGGTCCCGCTGCCGGTCCCGCTGCAGGCCCTGTTG

CAGCTCCCGCTGGGAAGCATCCGCGGATCGCCTTGGAGGAACCCGCCCCTCCGAAACCAC

CGAGGCCCATCAACATTGTCGATAACTACCCCCATCGCACATCCAATTCGGTGCTGTTGTCT

GGGACTTCCCGCGACCAAACCAAACAGCTATTTATGATCCCGGATGGCAGCGGGTCTGCCA

CGTCATACACCGAGATCGCCAAAGTTGGCGGTGGGTGGTGTGTCTGGGGCCTCTTCTCACC

CTTCATGAGAACACCGGAGGAGTATCATTGTGGTGTCTACGGCATGGCCACCAAGTTCATTG

ACCAGATGAAGTTCCGCCAGCCCCATGGCCCGTACTCACTTGCAGGGTGGAGTGCCGGCG

GCGTCATTGCATTTGAGATAGTCTACCAGTTGGTCACGGCCGGGGAAGAGGTTGCGAACCT

GATAATCATTGATGCCCCTTGCCCCGTCACCATCGAACCGCTTCCCCAAGGGCTCCACGCAT

GGTTCGCGTCAATCGGCCTGCTAGGCGAAGGCAACGACAAGAAGATTCCAGAGTGGCTTC

TTCCCCACTTTGCCGCCTCTATCACGGCTCTCAGCGAGTACGATGCCAGACCGATTCCCAAA

GACAAATGCCCCAAGGTGATGGCAATCTGGTGCGAGGACGGCGTGTGCCACCTACCCACC

GATCCTAAACCAGAGCCATATCCGAAGGGCCACGCCCTCTTTCTGCTGGAAAACCGCACCG

ACTTTGGGCCAAACAGGTGGGAGGAGTATTTGGACATCGACAAGATGCAATTCAGGCACAT

GCCTGGCAACCACTTTTCCATGATTCATGGCGAACAGGTATGTTGTGTGGCCTTCTAGTCAT

ATCTCATCCATAGCACCCGGTTTCGAGAGGCGAAAAAAAAGCTAACGCAGCGACACAGGC

CAAAATACTCGAAGGCTTTTTGAAGGAGGCCCTTCTAGATTGA

Result S3 Predicted amino acid sequence of *g657*

MPASGEQHRFVLFGGQGSPTVFSHTTAATAEEDVNSSSACSILLSKCHSAFLEDIASLDDRSQNL

LAIDSSRFSCPGHLLKPPALYHTHAVIQATTLYLCQVLHYLAETLRQYPNQSFQEVFDGLQETAG

FSSGLLPATVVARSRSVEVFLANGVRGFRFAFWIAYHSRAWSREAEARADDVASGHDHSPETTC

SLVIRGLTPGQVEERLCRYAATKNERNGSISTQSRQLQISAISSQTVVSVSGPRGELSNFRAHAVT

DVITAFAHIHGWYHGGDRLEAAVPLVLEDLQDRGATFPPCPHHCKSMRSTQDGALYVDSDTES

LGLSEWLVRHLLVHCVDWNRTASKITESVKTLLGLDAVTSVKVLSFGPSSGTLLAGLQPGNSRI

KVLDVSPFRAGNRTSLPNSHDDDIAIVGMSVNLPRGRGTEELWKTLSDGLSAVQTIPESRFKVS

DYYSTEKDAKSRSMPVKHGAFLADPFCFDNAFFNISPREAKSMDPQQRLLLHAAQEALEDAG

YVADSTPSFQRACTGCYIGVATGDYADNLRANIDVFYAPSTLRAFNGGRISYFYKFSGPTIVSDT

ACSSSLVSVYQACRALQQGDCTAAIAGGVNVISSPDIYLGLAKGHFLSPTGGCKSFDAAADGY

CRAEGCVLFVLKRLSAAVAENDRIHGVIRNVVVNQSGNARSITHPHSQTQIDLFHRLMKQVGD

ASEMESLKAVFGPHHSLANPLVISSIKSNIGHSEAASGAAGLAKLLLMLRHHKIPVQAGLRNINP

RFADMASAGLVTPSETAEWSHAHKTPRRALLNNFGASGSNASLLLEEWAAEPTKGQNINKLPE

RSAYVFALSTKSPKALERAIEQHIQYLRDSPSSTTSVEDICYTAVARRQIHEYRISVTCTSVNDLR

AKLESLGSLRTMGTMVPARRISRSVFLFSGQGSLYEGMGRELMSTLPQFRDVIAKCDTVLQGL

GYPSILSFFSGPSGHAGEILPEKDHVISSQCACVALEYALAVVFASWGIVPQYVMGHSLGEYVAL

CFSGVLTLEDTLHVVAARATMMTQQCAPNSTGMMACSLSSDGTNELISTTIGDVPELTVACING

INDCVVGGPLGQLERFQTHCQAMKLKTKVLETPFAFHTSAMDPILEPLKALGSSIKLSRPTTPII

STVFGRLLGDDDLSSDYFAAHARQPVLFTQSLSSMKASKAADDALFLELGPHPTLLPMVETFF

GTDISHACLGTLRKGQSAWVSLSRMLSEIYVLDSPVIWRQIFSPTSAKLVSLPGHPLEGSTYFTS

RREDRGPQASVPLDEDTSSTRRHTKTGLTLLPWMKSIDQSKETCLLETSLEVIGPLILGHDVGG

TPICPASVFHELALEGASMLLEPPDGYLMVVSGLNMTSPLVYEQSREVDVVTVHITKHSLGSG

ADFRISSCFDADLTEKVHCTGTIILTDFRTVEFRWSRDAAMVTRQSRYLNGPGKDDSSIFRTKVL

YQAVFPRVVRYSPEYQSLLHLAVADGNLEGIGLLRMPLGTQTRGYLAPPVFTDTLLHAAGFIAN

LGLRSDEVGICARVESMEISHREIDYLDTFTVYCSLLEVKGAILADTVALDSAGKVVAVVRGME

FKRLRLHTFQHLLSQRLSASDPWEFPRKVSAETSGSETINTPPTEASDISSSISHDAVRASLKNLV

KELGGFNERELDYEKPLEQLGIDSLMLIEIIAKLAHSFPCLAGSSRMALSECATLASLEHMLLESI

MGVSEARPLHQPTLTHTTAPSKPQKDPGWGNALRIPLMRGQRSSANGGMQAHNNLVALHLSP

ADMSSPLCLIHDGSGQVSMYARLRGHDRSTYAIFDPHFGSDNISFQSLSHMAEEYTSHLTQSRP

NTAIILGGWSFGGIAAFEMVRQLMAKGFDVKGLVLVDSPSPIDHQPLPDDVISYAIGLSDQKVA

SVVKSHDAIKNEFQSNASLLGSYEAVPLPRQTGRRSLPTVMLRSQRVLDTESLCGVRYDWLSK

QNARDASIVDWEGLIGGHIEVLPIPGNHFEVFSPENIGKTSAQVWKACRYIEKAIN

Result S4 Predicted amino acid sequence of *g4635*

MEAKLDESWAERPAFLLFGDQSLDSHSFLAQFYRQSKHGELARVFLQQANHALVGVVERLPA

LERATLPDFRTLRQLNERYHNAPLKHSGIDAALLTISQIAHYLDHAEKHCGDITQPHNTLLIGLC

SGLWAASAISVAPSLPDLVHVGVQTVLLAFKTGCYVHAIGQRLNPAFERSESWSYIFSGTSFED

VTGKLDAFHDASNLSPASRAYISAVSDNSTAISGPPSTLDAIVNNRILSADPIAIPVHGPYHAPHL

HSTADIERILELDKPETKDGLYKTRPRSPIMDCSTGTWYSPPDTKSLLISVASTILNRPLMFNKVL

NGCVEAARLFQDNRCLVLPLGPTQNSLTLQRRLQQEAGLDITLRMPPSTPSDATSSKIGNHGSS

GRPKLAIVGMAGRFPDAASHEALWKLLESGLAVHREVPPDRFNVKTHVDPSGKGKNMSHTPY

GCWIKDPGLFDHRVFNMSPREARNTDPMQRMALTTAYEALEMSGYVPNRTPSTRLDRIGTFYG

QTSDDWREINAAQDVDTYFITGGVRAFGPGRINYHFGFSGPSGMLGALGPRLRDTAIVGGLSC

MTNPDIFAGLSKGQFLSKKGPCATFDNDADGYCRGDGCASVVVKRLDDALADQDRVLAVILG

TATNHSADAISITHPHGPTQSTLSTAILDDAGVDPHDVDYVEMHGTGTQAGDGTEMKSVTDVF

APADRPRPEDRPLFLGAVKANVGHGEAASGVTALIKVLLMLEKNTIPPHVGIQKNGGVINKTFP

KDFAARNVNIAFQPVPFRRRDGKPRRVFVNNFSAAGGNTGLLVEDPPTVPPAKPDPRTHHVVT

LSGRVWESVKGNAERLLEWTERNRDTPLSHISYSTTARKLHHVCRMSVTAKEIGDLQAALRER

LRDLDLNQAIPAPHHPKVVMMFTGQGSQYAAMGREFYEHYAVFRESIDGFIDLARLQGFPSFLP

LIDGTDPNLAEMSPIVLQLGLACFEMALARLWGSWGIKPAAVVGHSLGEYAALEVAGVLSASD

VIYLVGSRAKLLVEKCKPGSHGMVAVQAPVQTVLELMGTEAGGLNIACINSPRETVISGDCEKS

KEMATHMSDQGYKSNHLHVPFAFHSPQVEVILDDFEKLAEGVSYNAPKIPIISTVHGNVIEGKSI

DAVYLRKHARDTVHFLDGLIEAQKLGTIDDKTVWLEIGPHPVLSNMVKATFGASTLAVPTLRR

TETCYKTLTGTLATLHNAHLKIDFNEYHRDFADSVRLLNLPAYSFNDKNYWIQYTGDWCLTKH

NLSVAAAEQKPATPWVPTTTVHKLNREIVEGGVAIVETESELHQEQLRNVLLRPQSKGIGCNVA

DMQVFKPLIFDDKAKSHILRLTVTANAEAGEADLVFHTAHGGKKVEHAHCKVYYGNHDEWQ

DEFDRASYLIKSRVDFLMEAEKRGAASKIGRGLAYKLFSALVDYNARYRGMEEVILDSTTCEA

TAKIRFQTTDQDGNFYFSPYHIDSSCHISGFIINGTDAVDSRERVFISHGWGSMRFTEIPNANKEY

RSYIRMQPVKGTEMMAGDAYIFDGDKIIGMTGRIKFQAIKRHTLNMMLPPRGGQVVSRPAPST

TKAAPSKKNKETVNPSNINKVNQKLNSVTAAVMDILVREIGCSHGELVDDASFDNLGADSLMA

LQVSSKIREELELDIEAQAWLDYPTIGAFKAYLATFEKPGRKERVPSRGSARTTDDESRDEESDS

DVTTPPEESDTHSIKGGMQDDAEPGDSAQNQKLRTIIRESIATEAGVDVQEVISATDWPSLGVD

SLLGLGISSRIRELAGIEVPTDLFLEHPTLKDVERVLGVTSPPKKPAARERQNPKEKSKAHTAAG
PAAGPAAGPVAAPAGKHPRIALEEPAPPKPPRPINIVDNYPHRTSNSVLLSGTSRDQTKQLFMIPD
GSGSATSYTEIAKVGGGWCVWGLFSPFMRTPEEYHCGVYGMATKFIDQMKFRQPHGPYSLAG
WSAGGVIAFEIVYQLVTAGEEVANLIIIDAPCPVTIEPLPQGLHAWFASIGLLGEGNDKKIPEWLL
PHFAASITALSEYDARPIPKDKCPKVMAIWCEDGVCHLPTDPKPEPYPKGHALFLLENRTDFGP
NRWEEYLDIDKMQFRHMPGNHFSMIHGEQAKILEGFLKEALLD

Table S1 Primers used in this study

Primer	Sequence (5'-3')
5-F	CTCCTCCTTTGGGTTCAG
5-R-f	TCCTTCAATATCATCTTCTGTCGAAGCCGCACCCGAGATAGT
3-F-r	ACTTGTTTAGAGGTAATCCTTCTTTGGGTTGAGGCGGATTCAG
3-R	TCAAACCGCAGTCGCTCC
PJ007	TCGACAGAAGATGATATTGAAGGAGCA
PJ008	AAGAAGGATTACCTCTAAACAAGTGTACCT
5-F-out	CACCTGCGTGCTATTTGCG
3-R-out	GACGATGCGGTAGAGTAGG
Y-HygR-R	GGCGAACTTAAGAAGGTATG
Y-HygR-DF	AACTCTCAAGCCTACAGGACAC
F-in	CCGAAGGGCTACACGGTCTC
R-in	CGTGTCGTGGATGAAGTGC
g7489-F	GACTTACCTATTCTACCCAAGCATATGGATGCCGCGGCCTAC
g7489-R-out	CGGTGTAACGACATGGTAGC
Trpc-R	ATGCTTGGGTAGAATAGGTAAG
gpda-F	GCATGCGGAGAGACGGACGGAC
G418-R	AGATCTATAGGAACTTCGGAATAGGAAC
PUARA-F1	CGCCCAATACGCAAACCG
PUARA-R1	CACTGTCCAATGCCAGAT
2.2-F-CE	CCGGAATTCGAGCTCGGTACCATGCCTGCCTCGGGAGAA
2.2-R-CE	TACTACAGATCCCCGGGTACCCTAATTGATTGCCTTCTCAATGTAACG
2.2-F	CGCTGGCTTGGTTACAC
2.2-R	TCGGTGTAACCAAGCCAGCG
2.2-F1	CCAGGCTTTACACTTTATGCTT
2.2-FN-1	AACCGGCAGTCTCCTGAAGC
2.2-in1	GTGTATGAGCAATCCCGTG
2.2-R2	ACCGTCCCCAATATAACCC
15.1-F1-CE	CCGGAATTCGAGCTCGGTACCATGGAGGCCAAACTCGACG
15.1-R1-CE	TAGATGACATCGCTAGCCGAGAGCACGCCGGCTACTTC
15.1-F-CE	TCGGCTAGCGATGTCATCTACC
15.1-R-CE	TACTACAGATCCCCGGGTACCTCAATCTAGAAGGGCCTCCTTCA
15.1-PR3	AAACAACAACAGCCGAGG
15.1-PF3	CATCCAATTCGGTGCTGT
P450-F-CE	CCGGAATTCGAGCTCGGTACCATGGAGACTTTCAACGTAAACCACT
P450-R-CE	TACTACAGATCCCCGGGTACCCTAATCCATATACTTCTCAAAGAAAGGC

Table S2 Gene clusters predicted by antiSMASH

No.	Type	Contig Name	Contig No.	Location	No. in contig	Cluster name	Gene cluster structure and most similar known cluster
1	T1PKS	000000F	1	1-38938	1	Cluster 1.1	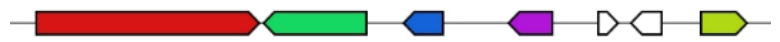
2	Fungal-RiPP-like	000001F	2	236692-328357	1	Cluster 2.1	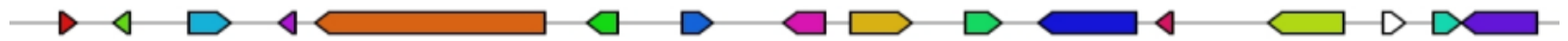
3	T1PKS	000001F	2	585593-693055	2	Cluster 2.2	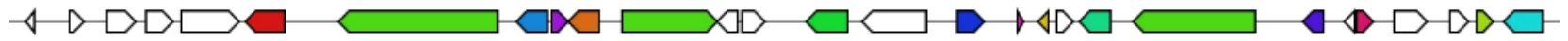
4	T1PKS	000002F	3	115797-206798	1	Cluster 3.1	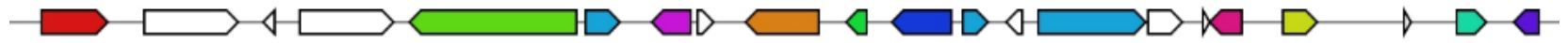
5	Indole	000002F	3	821829-853059	2	Cluster 3.2	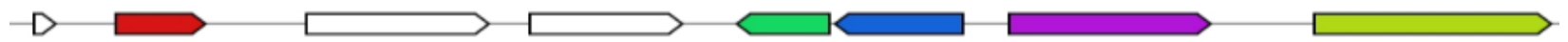 Terrequinone A (60% of genes show similarity)
6	Fungal-RiPP-like	000002F	3	1084005-1176116	3	Cluster 3.3	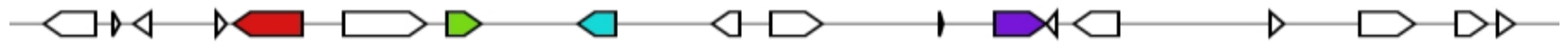
7	Terpene	000002F	3	1317125-1333620	4	Cluster 3.4	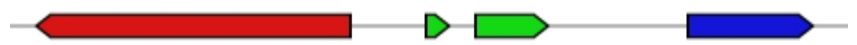
8	Fungal-RiPP-like	000003F	4	380405-471454	1	Cluster 4.1	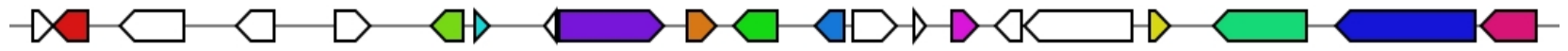
9	T1PKS	000003F	4	697775-767375	2	Cluster 4.2	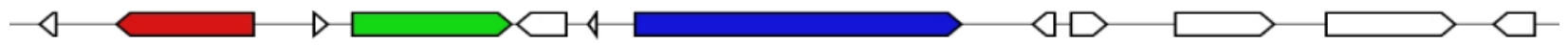
10	T1PKS	000004F	5	588291-651992	1	Cluster 5.1	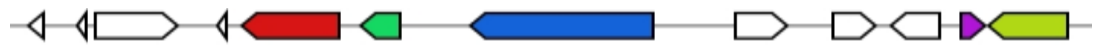
11	T1PKS	000004F	5	983587-1051751	2	Cluster 5.2	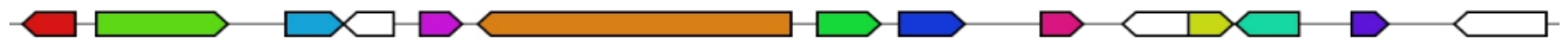 Solanapyrone D (33% of genes show similarity)
12	T1PKS	000005F	6	512037-576720	1	Cluster 6.1	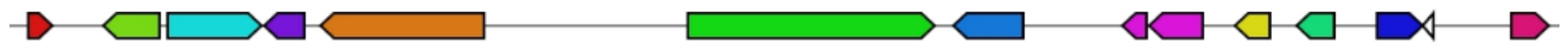
13	T1PKS	000005F	6	688534-757521	2	Cluster 6.2	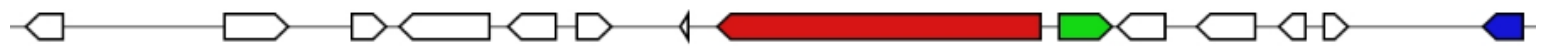 Shanorellin (57% of genes show similarity)
14	T1PKS	000006F	7	33342-91860	1	Cluster 7.1	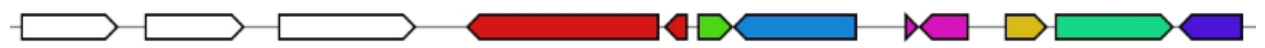 Agnestin A/Agnestin B (35% of genes show similarity)
15	T1PKS, NRPS	000006F	7	378802-445644	2	Cluster 7.2	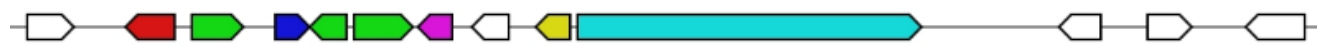

16	Terpene	000006F	7	725349-757350	3	Cluster 7.3	Chaetoglobosin P/ K/ A (16% of genes show similarity) 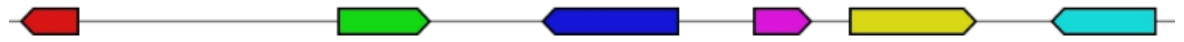
17	NRPS, T1PKS, prodigiosin	000006F	7	1058441-1158047	4	Cluster 7.4	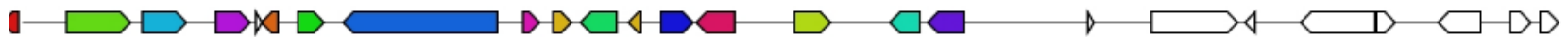
18	T1PKS, NRPS	000007F	8	33444-106074	1	Cluster 8.1	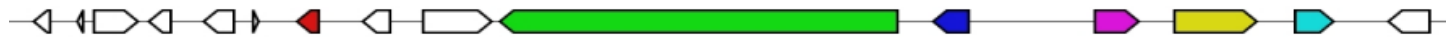
19	NRPS-like	000007F	8	267800-330379	2	Cluster 8.2	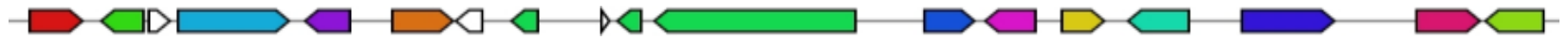
20	NRPS, fungal-RiPP-like	000007F	8	350585-487245	3	Cluster 8.3	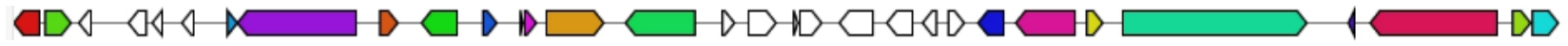
21	NRPS	000011F	12	631125-695153	1	Cluster 12.1	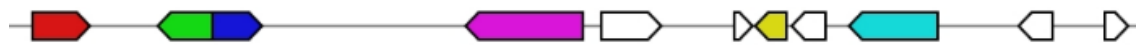
22	T1PKS	000013F	14	215706-286973	1	Cluster 14.1	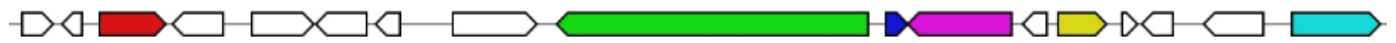
23	Terpene	000013F	14	289013-321368	2	Cluster 14.2	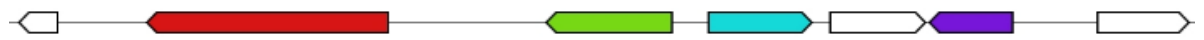
24	T1PKS	000014F	15	557284-630629	1	Cluster 15.1	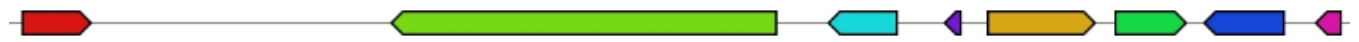 Scytalone/T3HN (40% of genes show similarity)
25	Terpene	000014F	15	677607-700690	2	Cluster 15.2	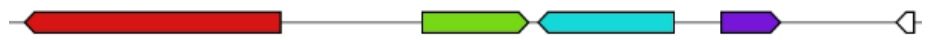
26	NRPS-like	000015F	16	435240-498512	1	Cluster 16.1	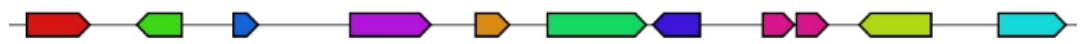
27	NRPS	000017F	18	247204-332524	1	Cluster 18.1	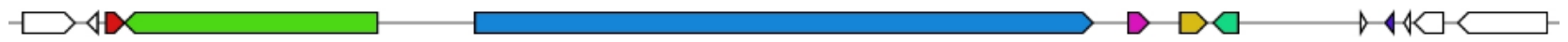
28	fungal-RiPP-like	000022F	23	180312-278321	1	Cluster 23.1	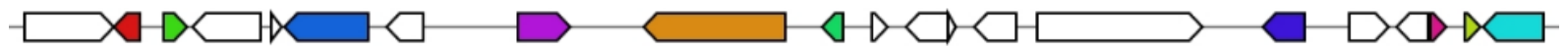
29	NRPS-like, T1PKS	000028F	29	179120-247154	1	Cluster 29.1	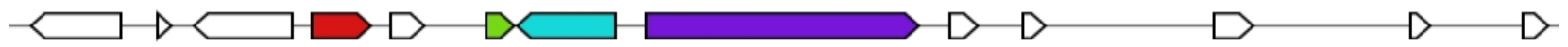
30	NRPS, T1PKS, NRPS-like	000029F	30	1-141694	1	Cluster 30.1	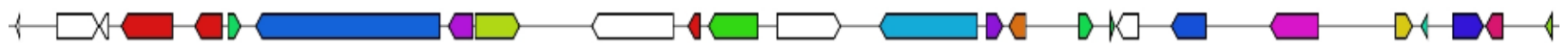 Chaetolivacine A/B/C (100% of genes show similarity)
31	fungal-RiPP-like	000030F	31	47979-143246	1	Cluster 31.1	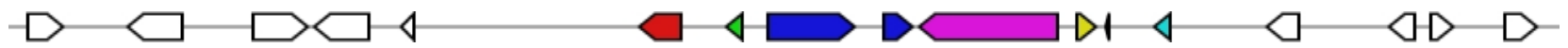
32	T1PKS	000030F	31	265095-330796	2	Cluster 31.2	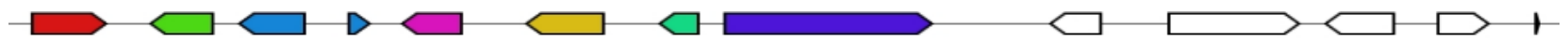 Neosartorin (47% of genes show similarity)

33	NRPS	000031F	32	183861-269793	1	Cluster 32.1	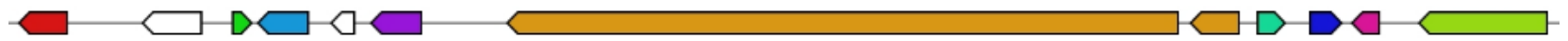
34	NRPS-like, terpene	000032F	33	235473-312042	1	Cluster 33.1	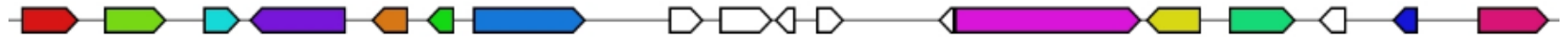 Patulin (20% of genes show similarity)
35	NRPS	000033F	34	92585-156545	1	Cluster 34.1	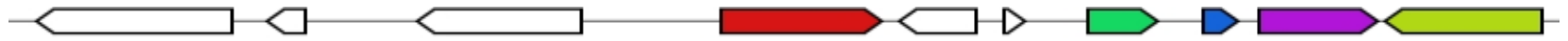
36	fungal-RiPP-like	000040F	41	1841-96586	1	Cluster 41.1	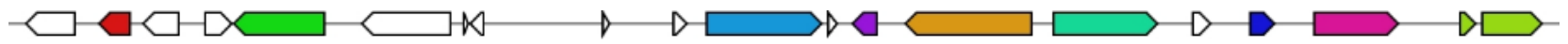
37	NRPS	000042F	43	77188-146118	1	Cluster 43.1	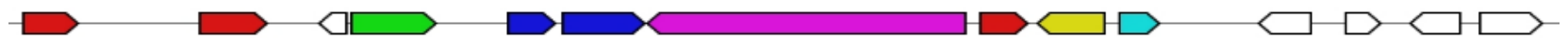 Chaetocin (60% of genes show similarity)
38	NI-siderophore, T3PKS	000044F	45	240276-305515	1	Cluster 45.1	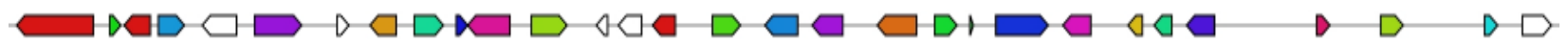
39	Terpene	000050F	51	182442-213004	1	Cluster 51.1	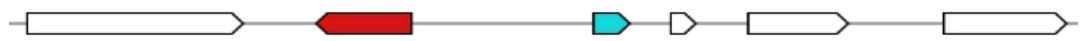
40	T1PKS	000051F	52	12720-80968	1	Cluster 52.1	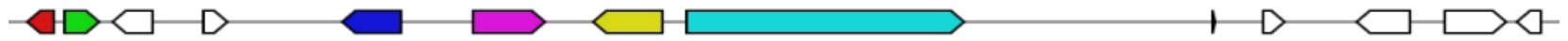
41	NRPS	000056F	57	97793-164057	1	Cluster 57.1	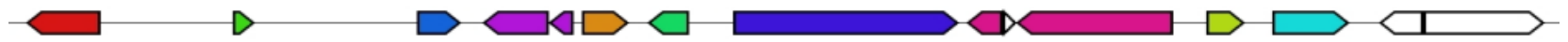
42	NRPS	000057F	58	66451-151508	1	Cluster 58.1	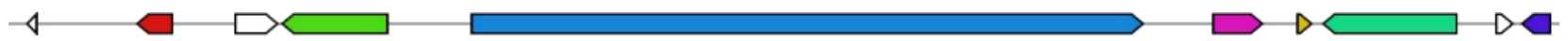
43	NRPS	000064F	65	1-64726	1	Cluster 65.1	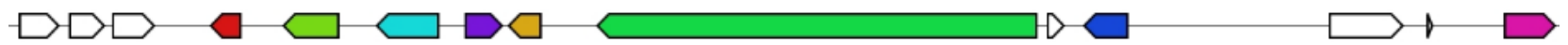
44	NRPS, T1PKS	000069F	70	47922-119626	1	Cluster 70.1	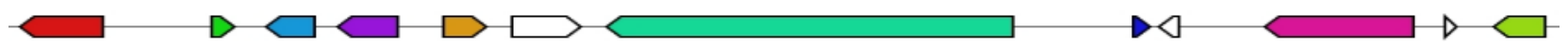
45	T1PKS	000076F	77	8236-76519	1	Cluster 77.1	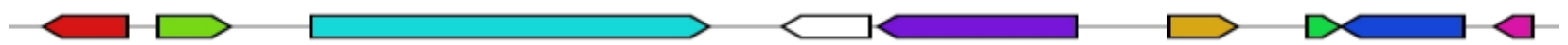
46	fungal-RiPP-like	000077F	78	1-80580	1	Cluster 78.1	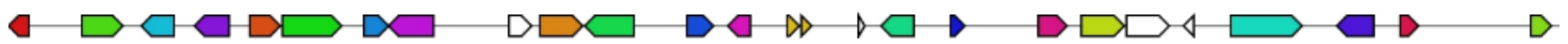

Table S3 Predicted gene functions of cluster 3.2

Gene	Amino acids (NO.)	Predicted protein function	Homologue
g1121	373	Endo-1,4-beta-xylanase	P79046.1
g1124	406	Indole prenltransferase tdiB	A7XRY3.1
g1125	312	Probable NADPH-dependent quinone reductase tidC	A7XRY6.1
g1126	421	Aminotransferase tdiD	A7XRY8.1
g1127	916	Nonribosomal peptide synthetase atqA	Q0D034.1
g1129	655	Oxygen-dependent choline dehydrogenase	Q4K4K7.1

Table S4 Domain organization and protein homologues of HRPKSs in *C. olivaceum* SD-80A. HRPKS, highly reducing polyketide synthases; Domains (predicted by Pfam and NCBI): SAT, starter-unit acyltransferase; KS, ketosynthase; AT, acyltransferase; PT, product template; DH, dehydratase; cMT, C-methyltransferase; ER, enoylreductase; KR, ketoreductase; ACP, acyl carrier protein; cAT, Choline/Carnitine O-acyltransferase; HET, heterokaryon incompatibility protein.

Cluster	Protein	PKS type	Domains	Protein Homologue	Percent Identity
1.1	H1.1	HRPKS	KS-AT-DH-cMT-KR-ACP	Q86ZD9.1	37.89%
2.2	H2.2-1	HRPKS	KS-AT-DH-cMT-KR-ACP-cAT	I1RVD8.1	32.90%
2.2	H2.2-2	HRPKS	KS-AT-DH-KR-ACP	A0A2Z5TM64.1	40.00%
3.1	H3.1	HRPKS	KS-AT-DH-KR-ACP	A0A4P8DJV2.1	45.30%
4.2	H4.2	HRPKS	KS-AT-DH-cMT-ER-KR-ACP	G3XMD1.1	60.36%
5.1	H5.1	HRPKS	KS-AT-DH-cMT-ER-KR-ACP	E5A7D9.1	46.62%
5.2	H5.2	HRPKS	KS-AT-DH-cMT-ER-KR	D7UQ44.1	49.06%
6.1	H6.1	HRPKS	KS-AT-DH-ER-KR-ACP	N4WHA7.1	31.34%
6.2	H6.2	HRPKS	SAT-KS-AT-DH-ACP-cMT	A0A097ZPE0.1	44.71%
14.1	H14.1	HRPKS	KS-AT-DH-cMT-ER-KR-ACP-HET	A0A1L7U3D7.1	36.19%
30.1	H30.1	HRPKS	KS-AT-DH-ER-KR-ACP	N4WHA7.1	33.99%
52.1	H52.1	HRPKS	KS-AT-DH-ER-KR-ACP	D2E9X0.1	50.31%
77.1	H77.1	HRPKS	KS-AT-DH-cMT-ER-KR-ACP	A0A6S5ZY48.1	49.41%

Table S5 Predicted gene functions of cluster 2.2

Gene	Amino acids (NO.)	Predicted protein function	Homologue
g653	3117	Highly reducing polyketide synthase PKS6	I1RVD8.1
g654	457	UDP-glucosyltransferase A1	I1RVD8.1
g655	228	Decarboxylase macB	A0A2P1DP90.1
g656	502	Cytochrome P450	G1XU03.1
g657	2044	Orsellinic acid synthase	A0A2H3CTK0.1
g659	310	*O*-methyltransferase	A0A348AXX3.1
g664	427	NADH:flavin oxidoreductase	I1RV17.1
g670	2241	Polyketide synthase-nonribosomal peptide synthetase hybrid himA	A0A2Z5TM64.1
g676	928	D-inositol 3-phosphate glycosyltransferase	A1T3B5.1
g677	233	Superoxide dismutase	P07895.2

Table S6 Predicted gene functions of cluster 15.1

Gene	Amino acids (NO.)	Predicted protein function	Homologue
g4633	919	Transcription factor	W3X9K7.1
g4634	303	Ubiquitin carboxyl-terminal hydrolase 8	Q80U87.2
g4635	2142	Polyketide synthase	W3X7U2.1
g4636	1382	Multicopper oxidase	W3X7K0.1
g4637	213	-	Q7MN49.1
g4638	158	Putative RNA polymerase II transcriptional coactivator	Q872F4.1
g4639	1045	Meiotically up-regulated gene 122 protein	O74444.1
g4639	519	Uncharacterized transporter	Q9P6J0.1
g4641	894	-	Q9P6R4.1
g4642	323	Ribosomal RNA-processing protein 7 homolog A	Q9D1C9.1

Table S7 Fungal PKSs used to create the phylogenetic tree (see Fig. S21-S23). Products: OA, orsellinic acid; OAE orsellinic acid ester; 3-MOA, 3-methylorsellinic acid; 5-MOA, 5-methylorsellinic acid; DMOA, 3,5-dimethylorsellinic acid; LA, lecanoric acid.

PKS	Product	NCBI Accession Number
AflC	Norsolorinic acid anthrone	ACH72912.1
AndM	DMOA	A0A097ZPE0.1
ArmB	OA, OAE	I3ZNU9.1
AscC	OA	A0A455R5P9.1
AtCURS2	Dihydroxyphenylacetic acid lactone	AGC95321.1
Atr1	4-O-demethylbarbatic acid	QXF68953.1
CC1G_05377	OA	XP_001835415.2
CcRadS2	Resorcylic acid lactone	ACD39770.1
Cla3	Cladosporin	A0A125R003.1
Clz14	Zaragozic acid A	A0A345BJN0.1
CTB1	Nortoralactone	Q6DQW3.1
Dhc5	10,11-Dehydrocurvularin	A0A0N7D745.1
DuxI	Phenalenone aromatic	AWS21681.1
HerA	OA	TFY83890.1
LovF	Lovastatin	Q0C8L6.1
MapC	5-MOA	F1DBA9.1
MpaC'	5-MOA	AJG44381.1
OesA	OA, OAE	UID85533.1
Ops1	OA	J4UHQ6.1
OrsA	OA, diaryl ether	Q5AUX1.1
PkbA	3-MOA	EAA58470.1
PKS1	OA	APH07629.1
PKS14	OA	XP_009256703.1
PKS2	OAE	APH07628.1
PKS63787	OA	AST08390.1
PksA	Norsolorinic acid	Q12053.1
PksP	Naphthopyrone	XP_756095.1
PksST	Norsolorinic acid	Q12397.2
PoxF	Oxaleimide	A0A1W5T1T1.1
Preu3	3-MOA	OK493437.1
Preu6	LA	P9WET2.1
PrhL	DMOA	A0A1E1FFN8.1
PspA	Soppiline	P0DUK1.1
RADS2	Monocillin	C5H882.1
Rdc1	7',8'-dihydrozearalenol	B3FWT6.1
StbA	OA	A0A193PS74.1
TerA	OA	XP_001210231.1
VirA	Salicylaldehyde	XP_013952638.1

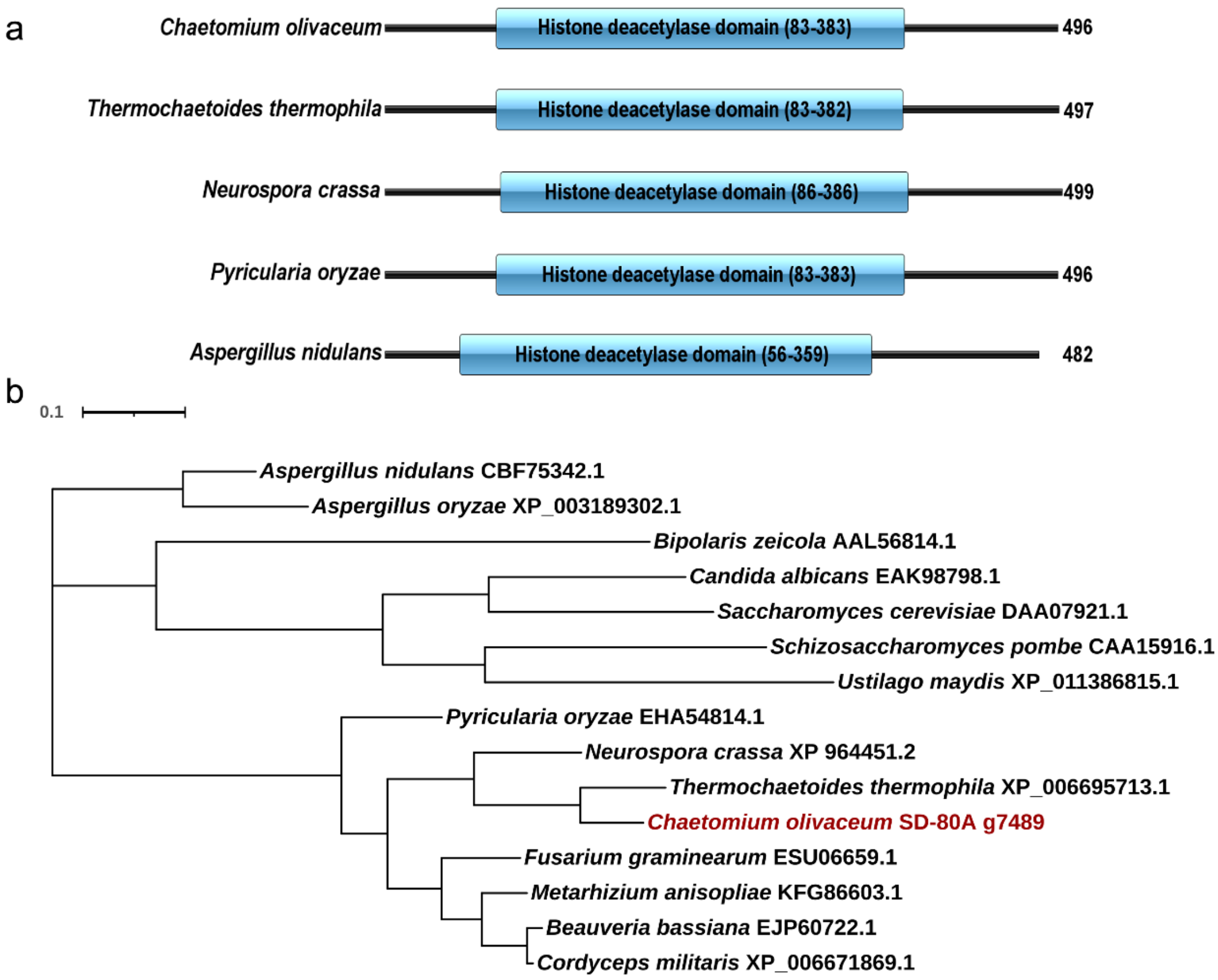

Fig. S1 Domain prediction of Hos2 orthologs found in *Chaetomium olivaceum*, *Thermochaetoides thermophila*, *Neurospora crassa*, *Pyricularia oryzae*, and *Aspergillus nidulans* (**a**). Phylogenetic relationship of *C. olivaceum* g7489 with the orthologs found in other representative fungi (**b**). The domain of each protein was predicted using the NCBI database. The maximum likelihood method in MEGA7 software was used for the construction of phylogenetic tree. Each fungal name is followed by the NCBI accession number of each protein.

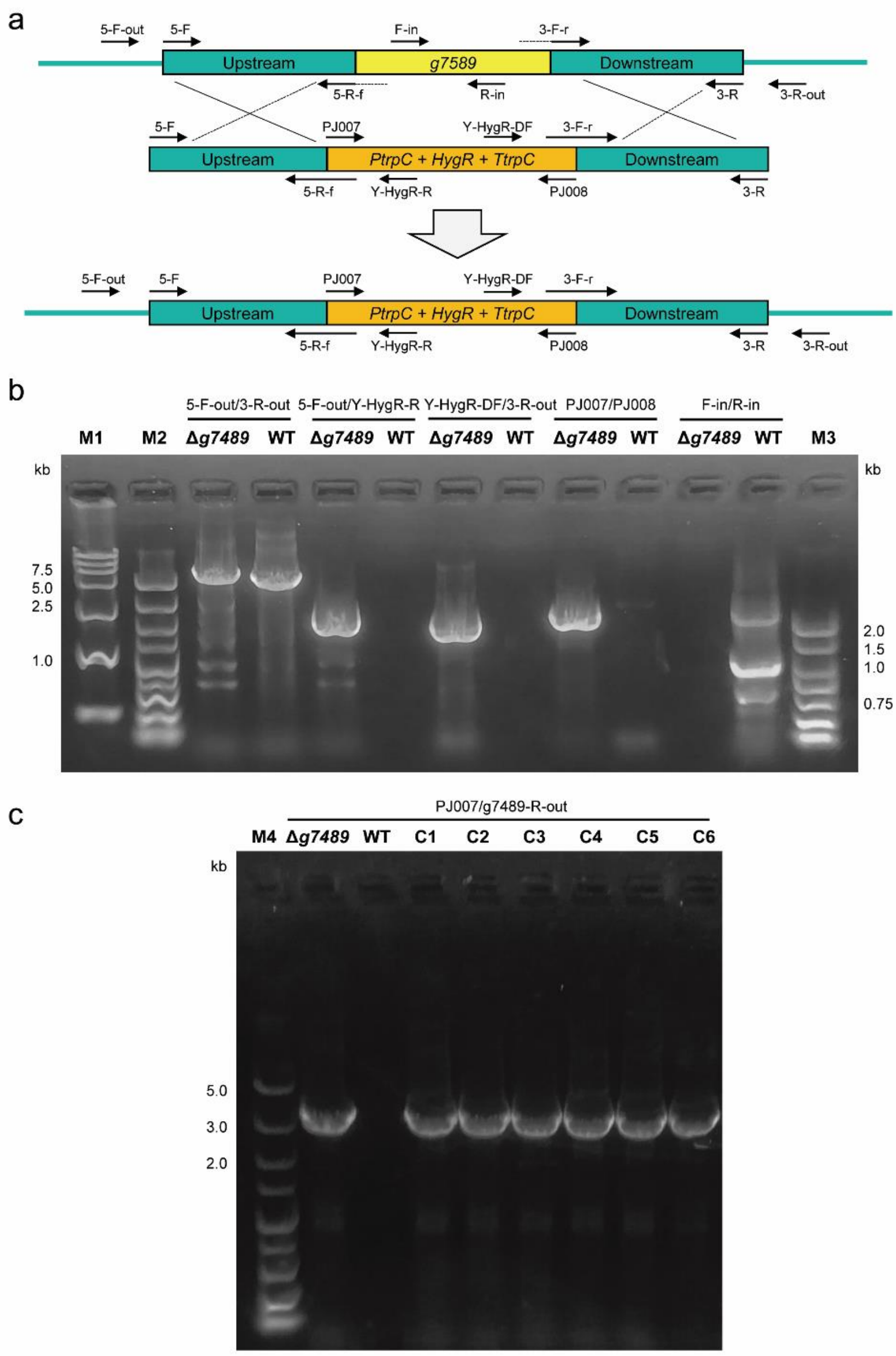

Fig. S2 Schematic representation of gene disruption strategy (**a**). Electropherogram of PCR products (**b** and **c**). M1, DL15000 DNA Marker; M2, DL5000 DNA Marker; PCR products amplified with primers 5-F-out/3-R-out (Δ*g7489*, 5456 bp; WT, 4764 bp), 5-F-out/Y-HygR-R (Δ*g7489*, 1992 bp; WT, no product), Y-HygR-DF/3-R-out (Δ*g7489*, 1850 bp; WT, no product), PJ007/PJ008 (Δ*g7489*, 2131 bp; WT, no product), and F-in/R-in (Δ*g7489*, no product; WT, 1047 bp); M3, DL2000 Plus DNA Marker; M4, DL5000 DNA Marker; PCR products amplified with primers PJ007/g7489-R-out (Δ*g7489*, 3042 bp; WT, no product; C1-C6, 2715 bp).

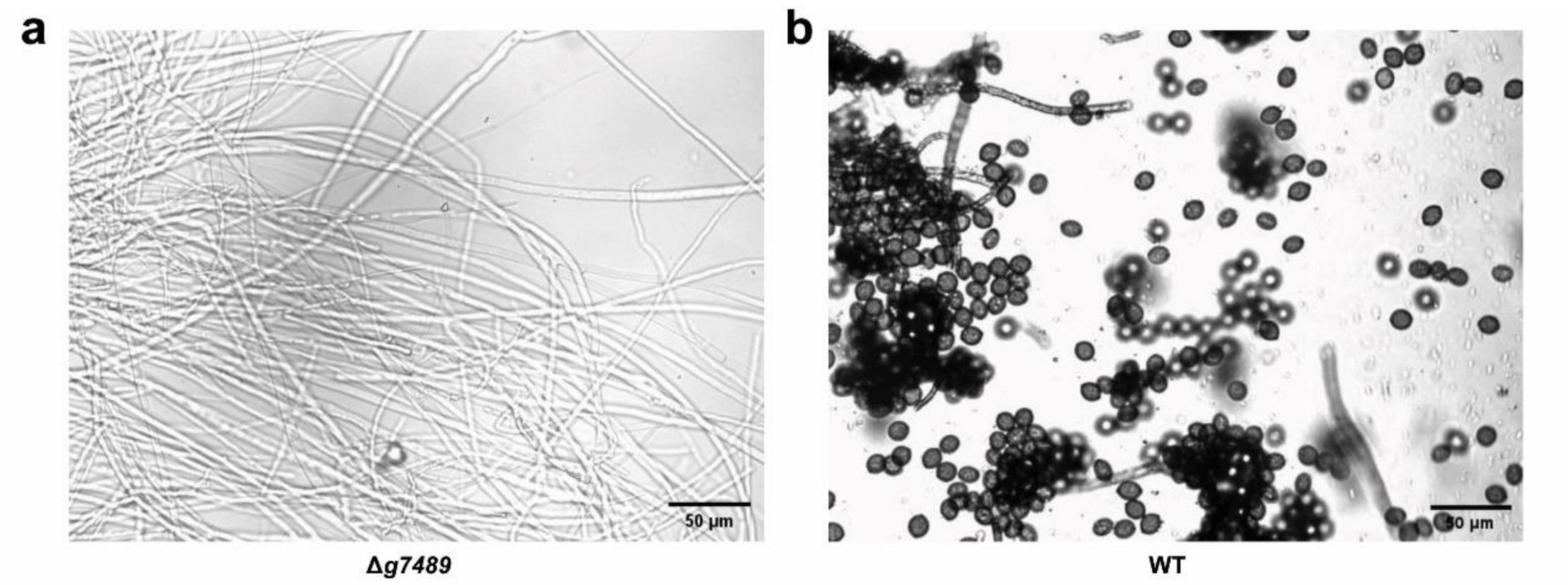

Fig. S3 Micrographs of Δg*7489* (**a**) and WT (**b**)

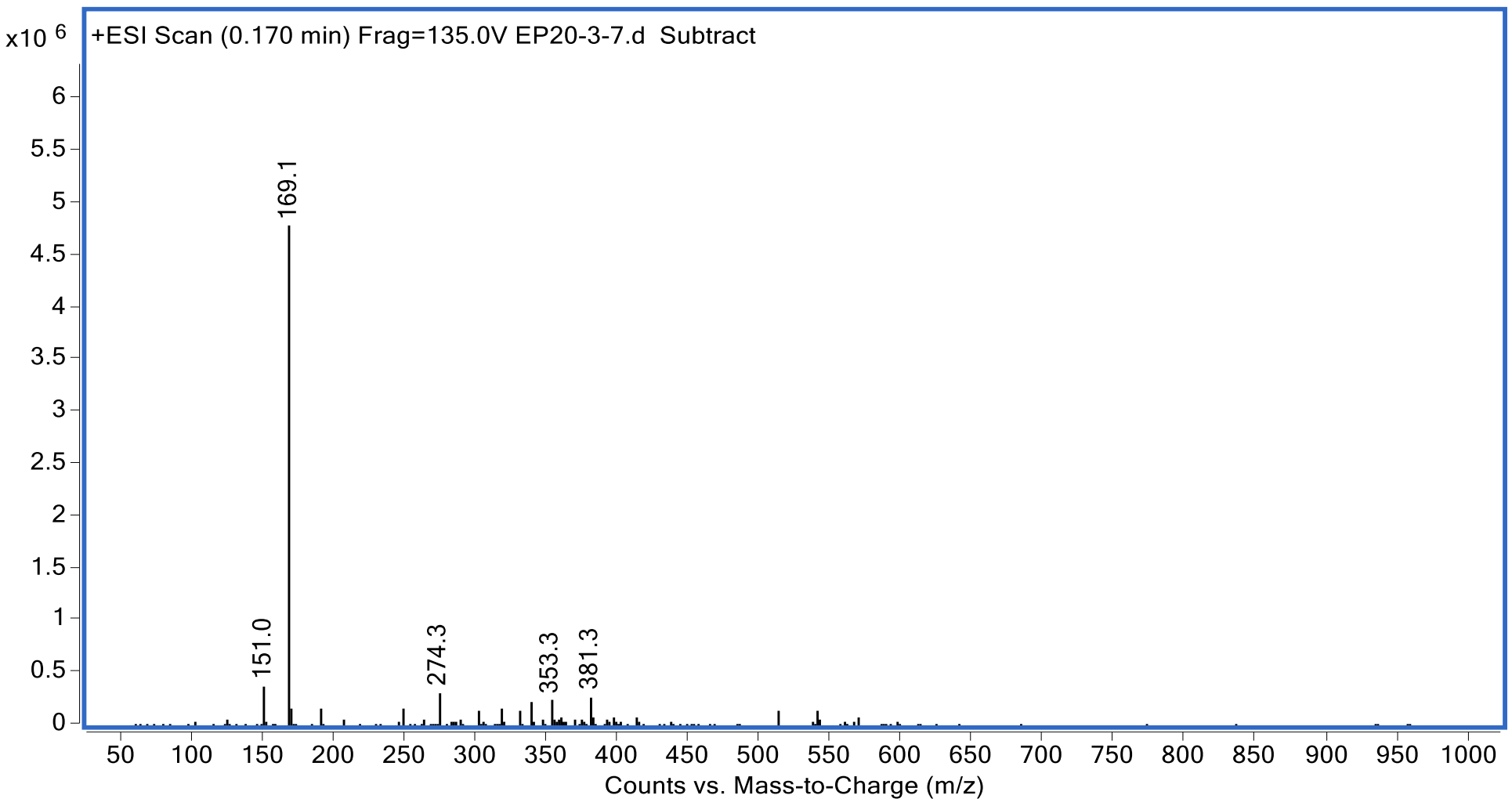

Fig. S4 Mass spectrum of compound **A1** *m*/*z* 169.10 $[M + H]^+$

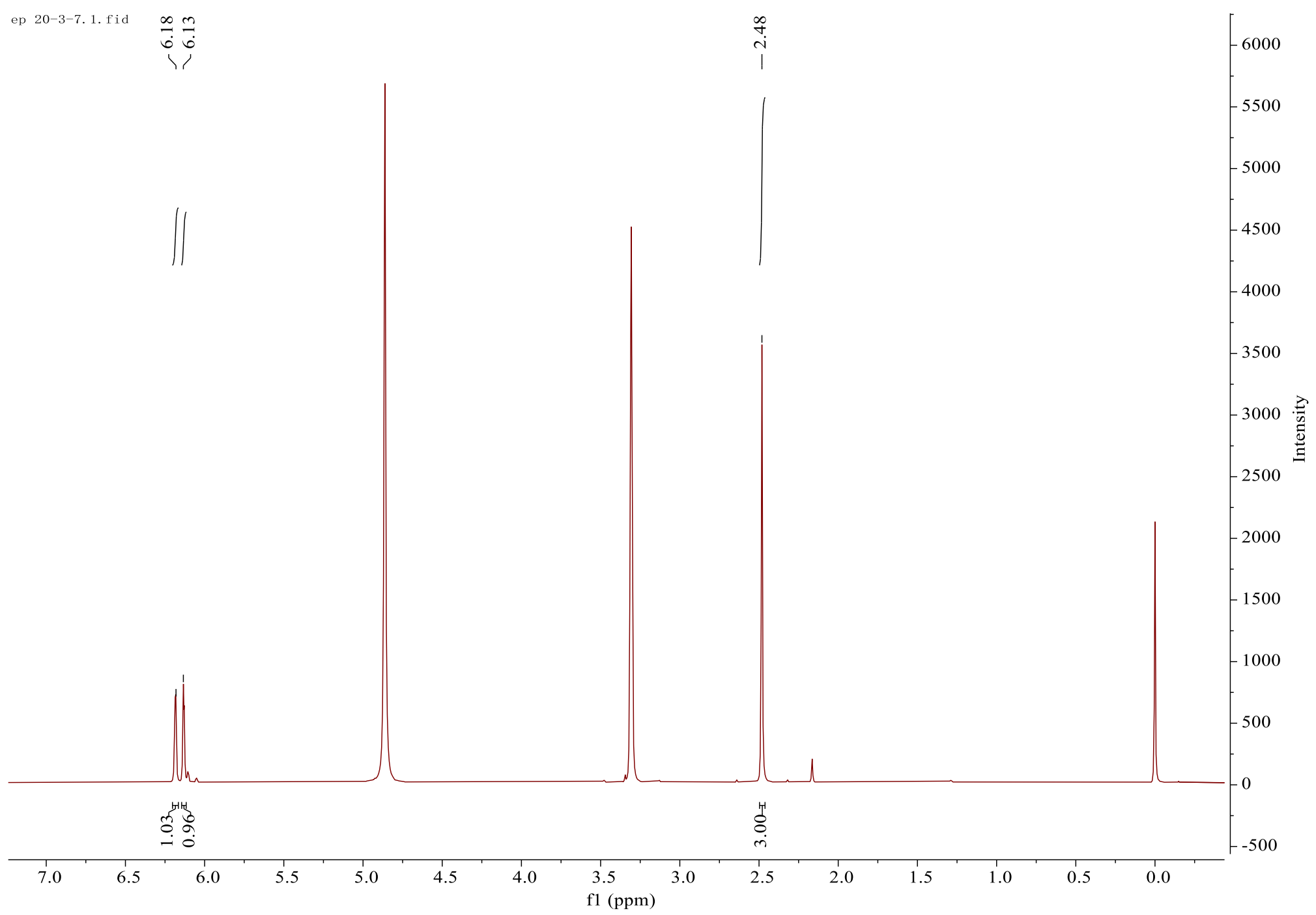

Fig. S5 ^{1}H NMR spectrum of compound **A1** (400 MHz, MeOD): δ 6.18 (d, J = 2.75 Hz, 1H, H-5), 6.13

(d, J = 2.34 Hz, 1H, H-3), and 2.48 (s, 3H, CH_3)

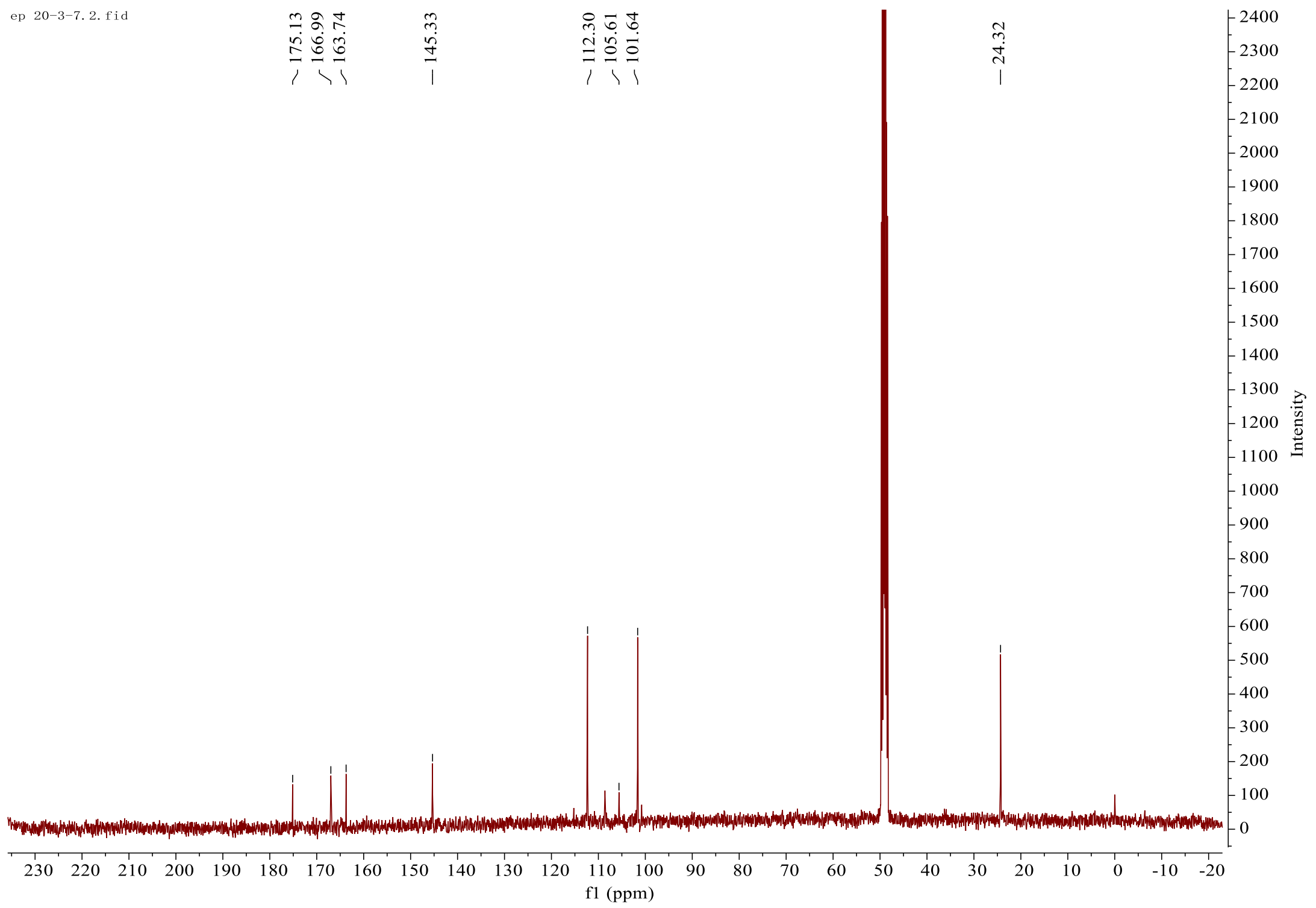

Fig. S6 ^{13}C NMR spectrum of compound **A1** (100 MHz, MeOD): δ 175.13 (COOH), 166.99 (C-4), 163.74 (C-2), 145.33 (C-6), 112.30 (C-5), 105.61 (C-1), 101.64 (C-3), and 24.32 (CH_3)

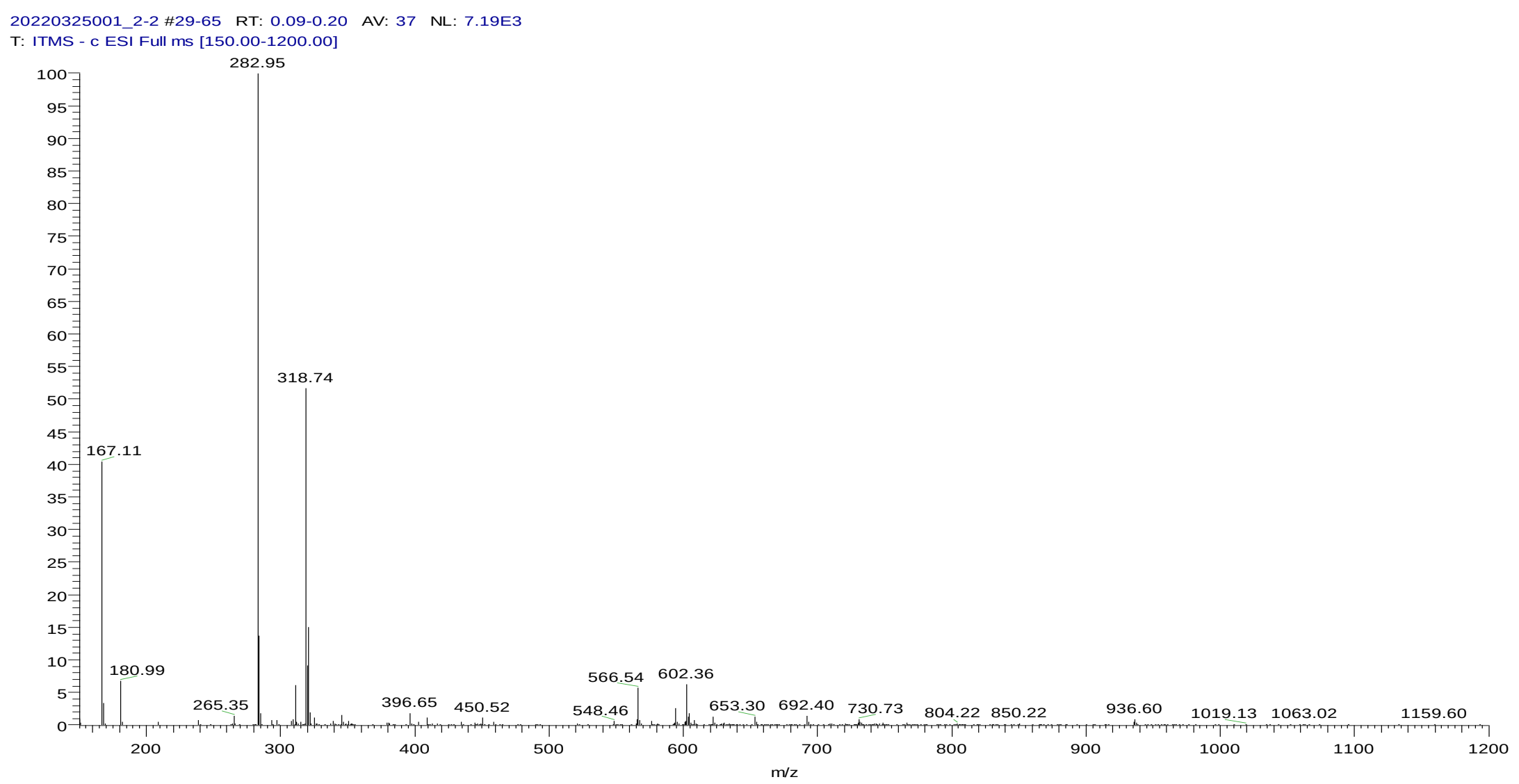

Fig. S7 Mass spectrum of compound **A2** m/z 282.95 $[M - H]^-$

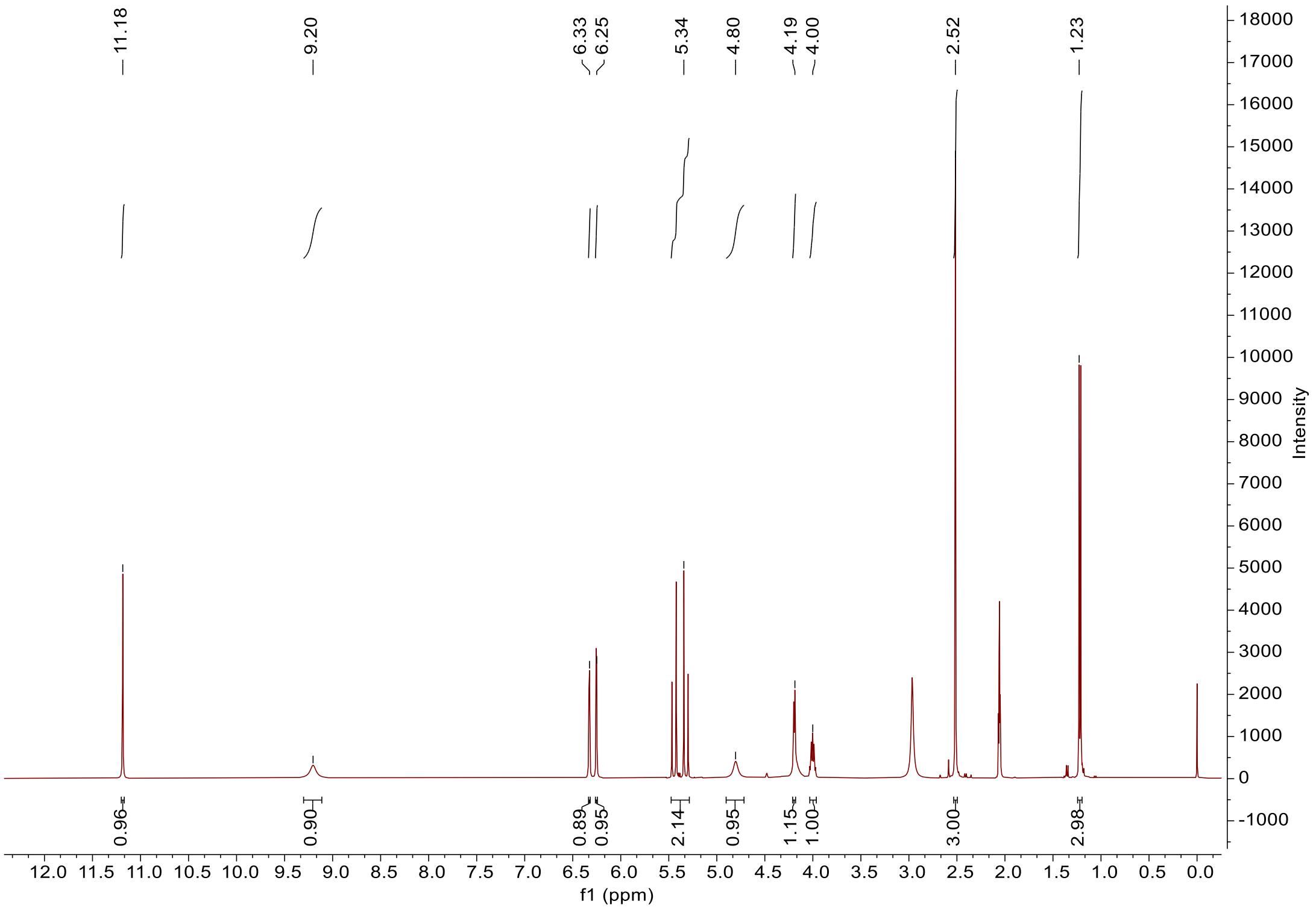

Fig. S8 ^{1}H NMR spectrum of compound **A2** (400 MHz, Acetone-D_6): *δ* 11.18 (s, 1H, OH), 9.20 (brs, 1H, OH), 6.33 (d, *J* =2.96 Hz, 1H, H-3), 6.25 (d, *J* =2.33 Hz, 1H, H-5), 5.34 (dd, *J* =17.68, 17.45 Hz, 2H, H-1'), 4.80 (brs, 1H, OH), 4.19 (d, *J* =4.37 Hz, 1H, H-3'), 4.00 (m, 1H, H-4'), 2.52 (s, 3H, CH_3), and 1.23 (d, *J* =2.33 Hz, 3H, H-5')

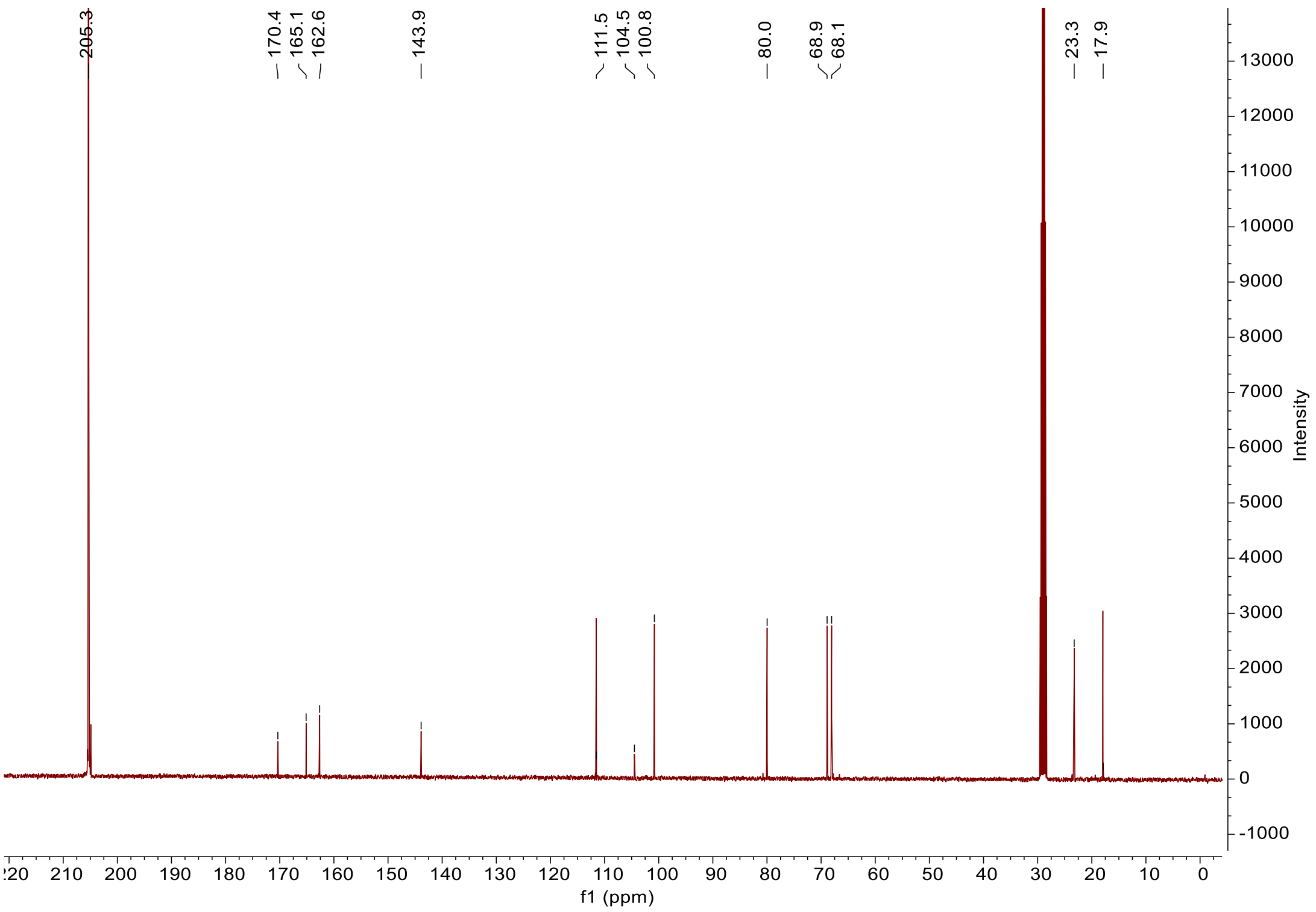

Fig. S9 ^{13}C NMR spectrum of compound **A2** (100 MHz, Acetone-D_6): *δ* 205.3 (C-2'), 170.4 (C-7), 165.1

(C-4), 162.6 (C-2), 143.9 (C-6), 111.5 (C-5), 104.5 (C-1), 100.8 (C-3), 80.0 (C-3'), 68.9 (C-4'), 68.1 (C-1'), 23.3 (CH_3), and 17.9 (CH_3)

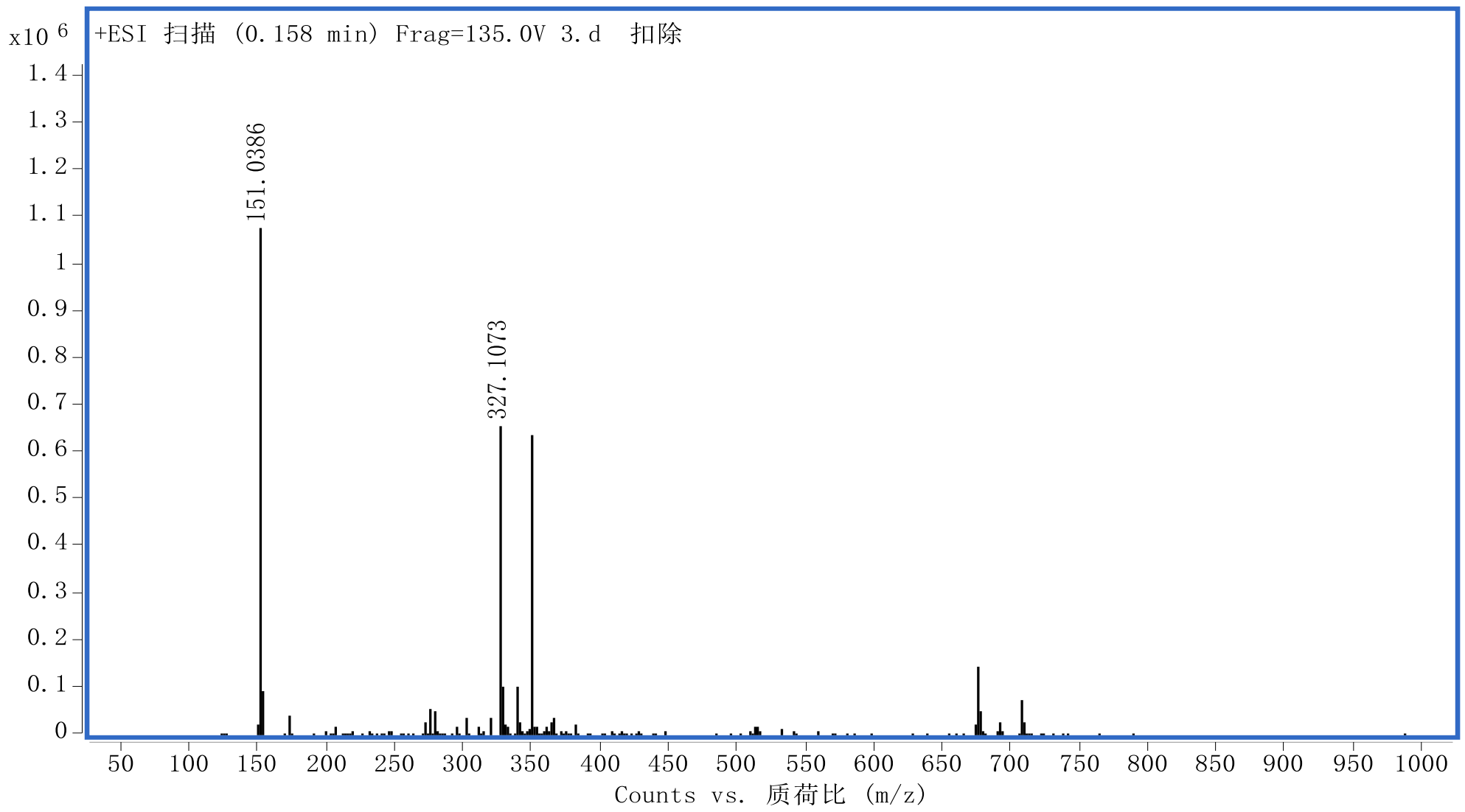

Fig. S10 Mass spectrum of compound **A3** *m/z* 327.1073 $[M + H]^+$

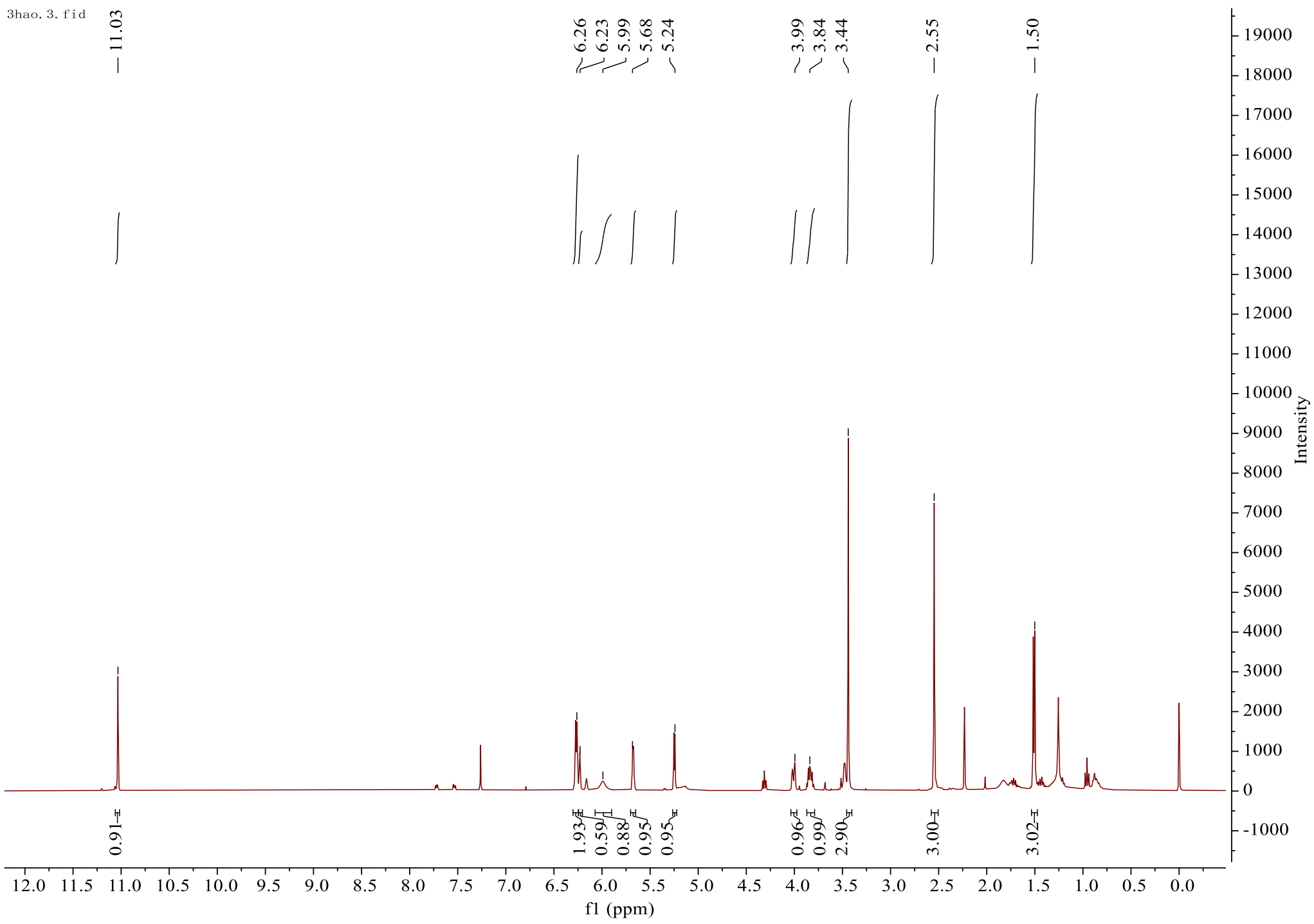

Fig. S11 ^{1}H NMR spectrum of compound **A3** (400 MHz, $CDCl_3$): δ 11.03 (s, 1H, OH), 6.26 (d, J = 4.78 Hz, 1H, H-5), 6.23 (s, 1H, H-3), 5.99 (brs, 1H, OH), 5.68 (d, J = 4.49 Hz, 1H, H-2'), 5.24 (d, J =4.09 Hz, 1H, H-1'), 3.99 (d, J =10.03 Hz, 1H, OH,), 3.84 (m, J =27.82 Hz, 1H, H-5'), 3.44 (s, 3H, OCH_3), 2.55 (s,

3H, H-8), and 1.50 (d, J =6.0 Hz, 3H, H-6')

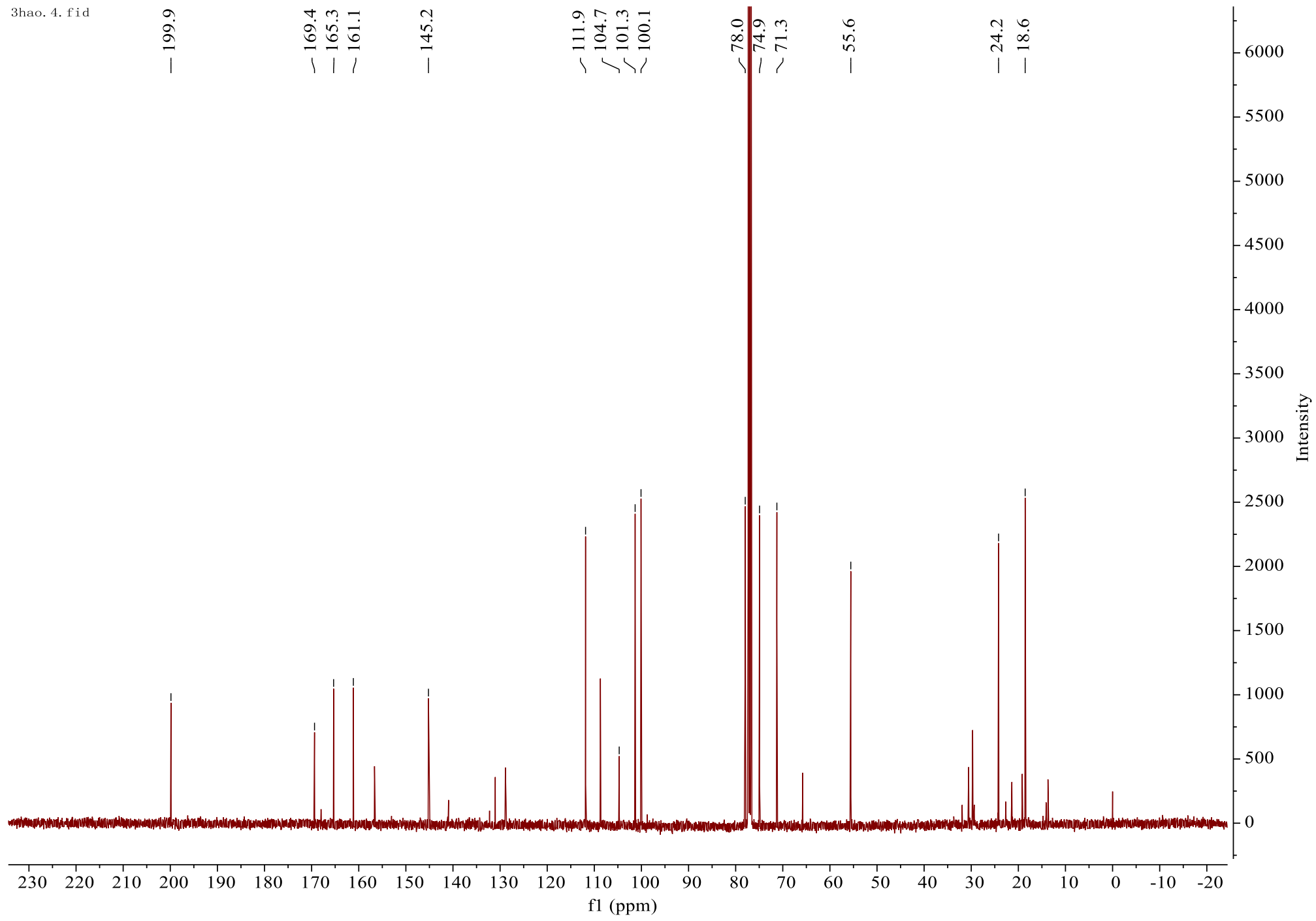

Fig. S12 ^{13}C NMR spectrum of compound **A3** (100 MHz, $CDCl_3$): δ 199.9 (C-3'), 169.4 (C-7), 165.3 (C-2), 161.1 (C-4), 145.2 (C-6), 111.9 (C-5), 104.7 (C-1), 101.3 (C-3), 100.1 (CH_3), 78.0 (C-4'), 74.9 (C-2'), 71.3 (C-5'), 55.6 (OCH_3), 24.2 (CH_3), and 18.6 (CH_3)

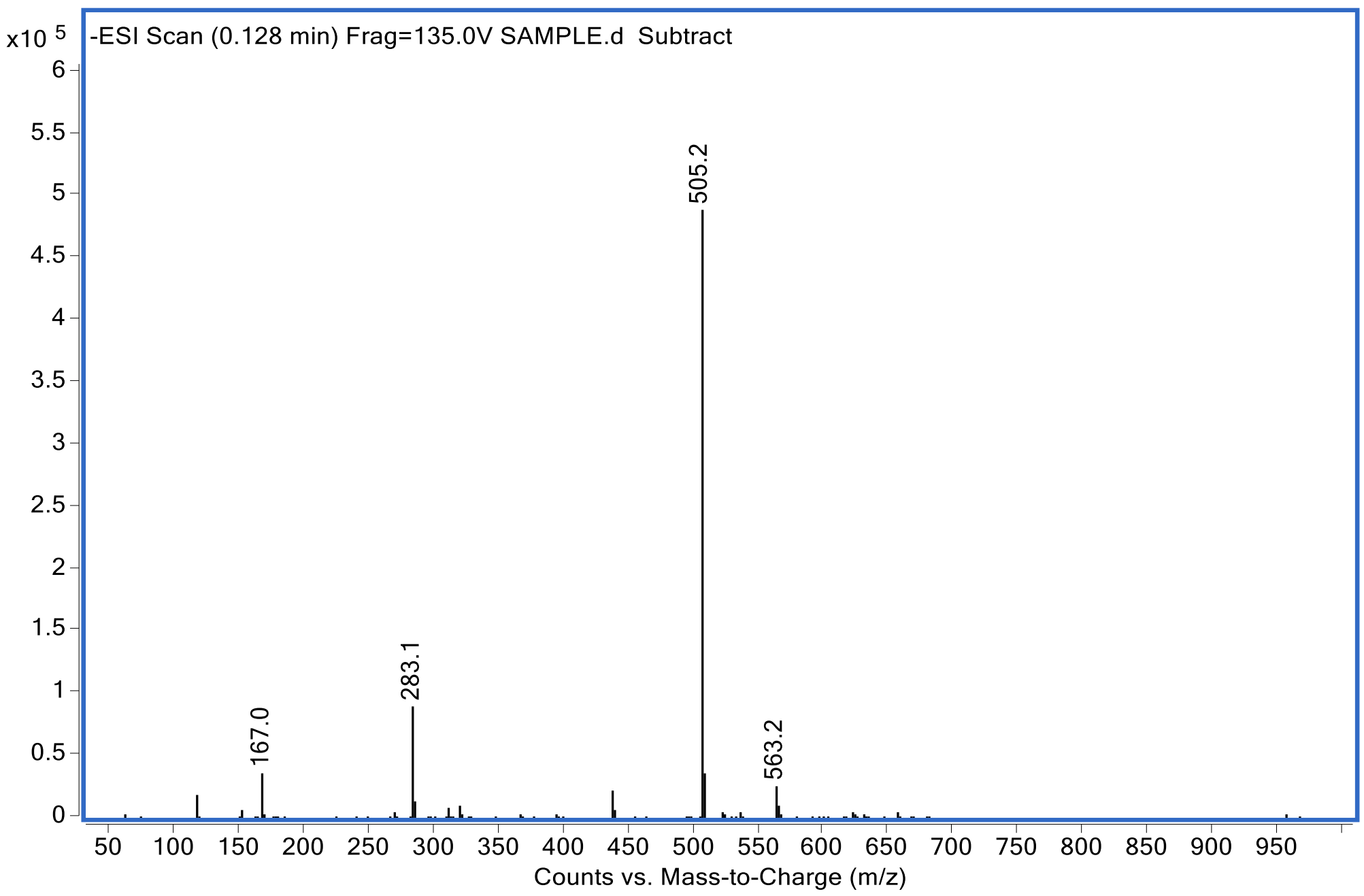

Fig. S13 Mass spectrum of compound **A4** m/z 505.2 $[M - H]^-$

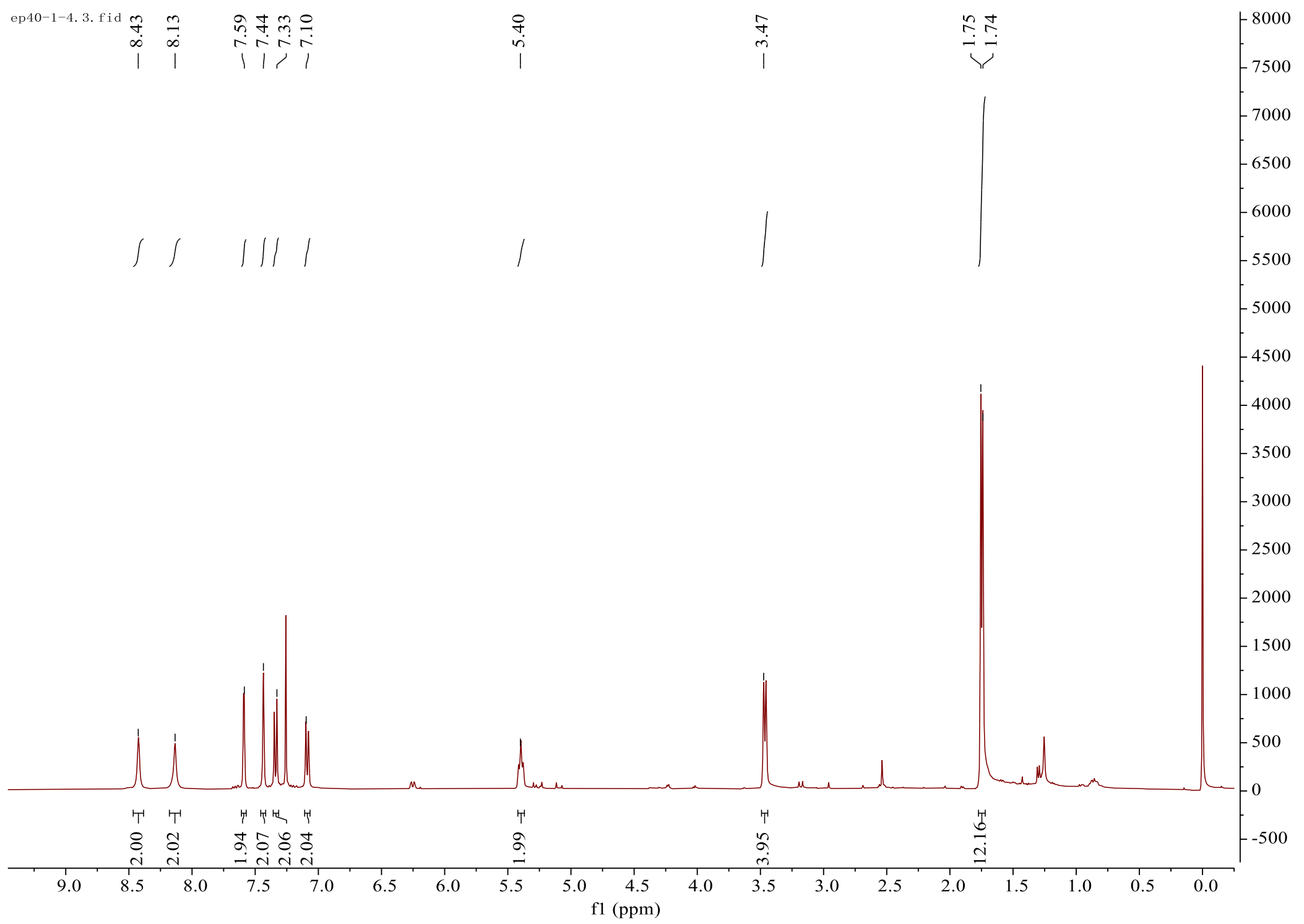

Fig. S14 ^{1}H NMR spectrum of compound **A4** (400 MHz, $CDCl_3$): *δ* 8.43 (brs, 1H, OH), 8.13 (brs, 1H, NH), 7.59 (d, 1H, *J* =2.7 Hz, H-2'), 7.44 (s, 1H, H-4'), 7.33 (d, 1H, *J* =8.38 Hz, H-7'), 7.10 (d, 1H, *J* = 8.38 Hz, H-6'), 5.40 (t, 1H, *J* = 15.03 Hz, H-11'), 3.47 (m, 1H, H-10'), 1.75 (s, 3H, H-13'), and 1.74 (s, 1H, H-14')

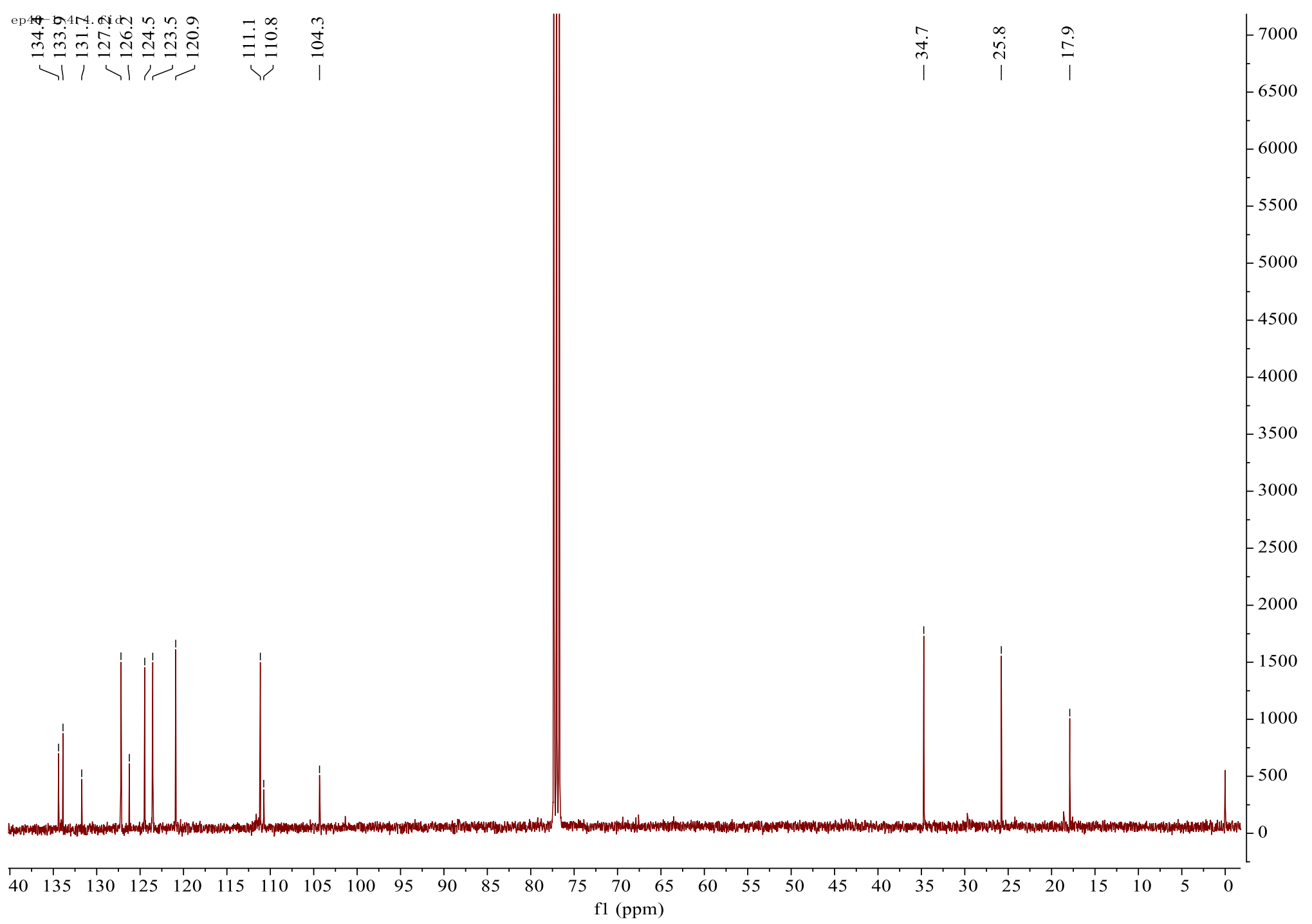

Fig. S15 ^{13}C NMR spectrum of compound **A4** (100 MHz, $CDCl_3$): *δ* 134.4 (C-2'), 133.9 (C-8'), 131.7

(C-5'), 127.2 (C-12'), 126.2 (C-6), 124.5 (C-9'), 123.5 (C-6'), 120.9 (C-4'), 111.1 (C-7'), 110.8 (C-3), 104.3 (C-3'), 34.7 (C-10'), 25.8 (CH_3), and 17.9 (CH_3)

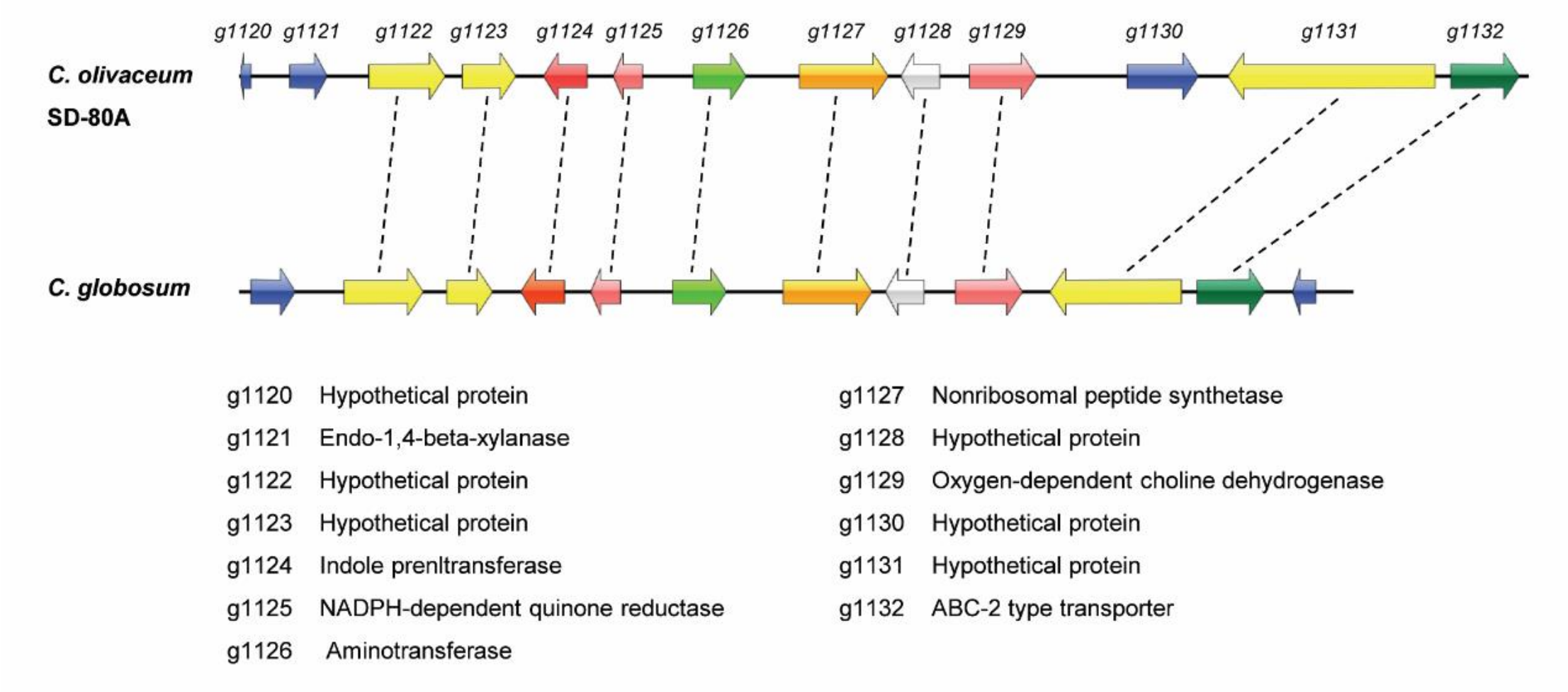

Fig. S16 Comparison of cluster 3.2 and cochliodinol BGC from *C. globosum*

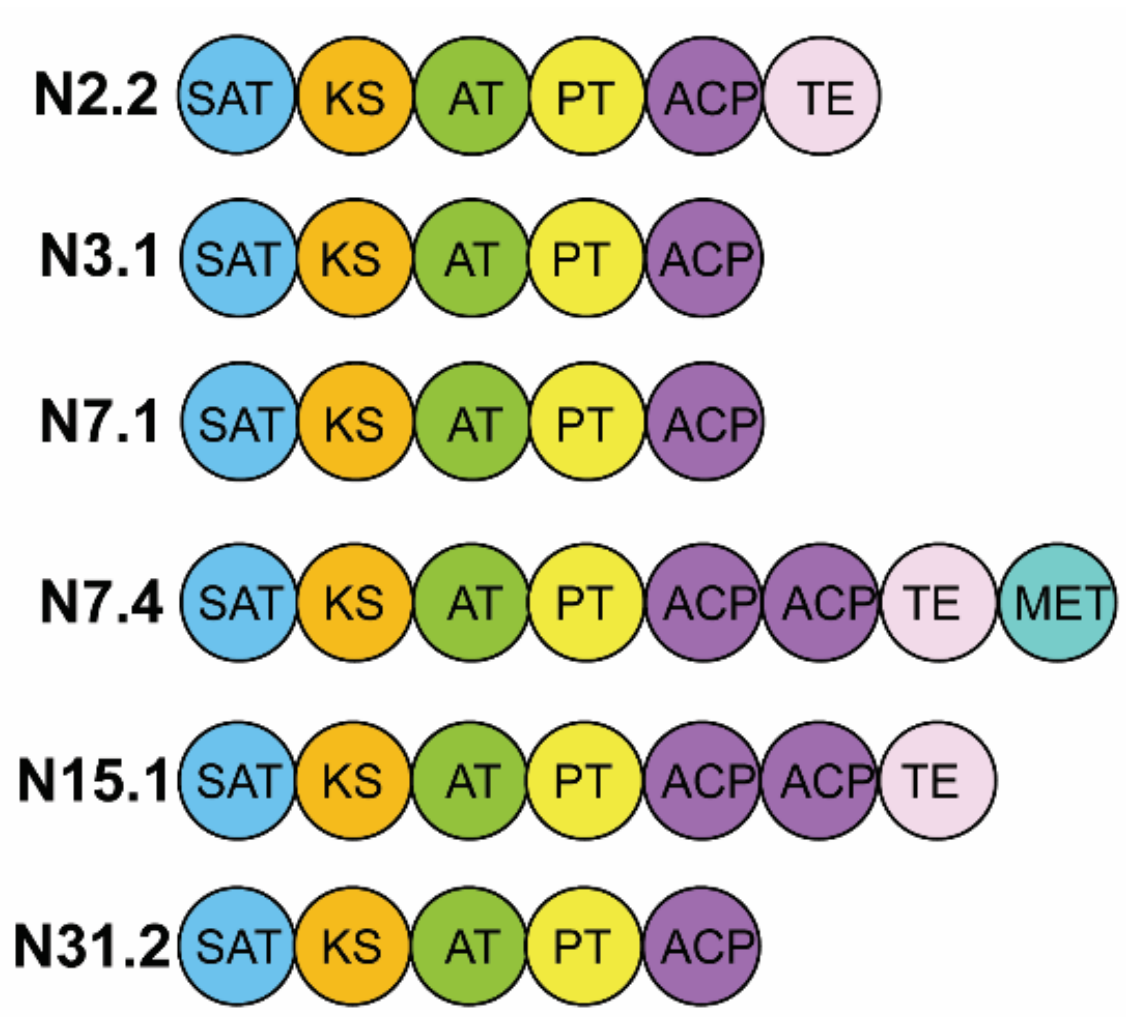

Fig. S17 Domain organization of NRPKSs of *C. olivaceum* SD-80A. SAT, starter acetyltransferase; KS, ketosynthase; AT, acyl transferase; PT, product template; ACP, acyl carrier protein; TE, thioesterase.

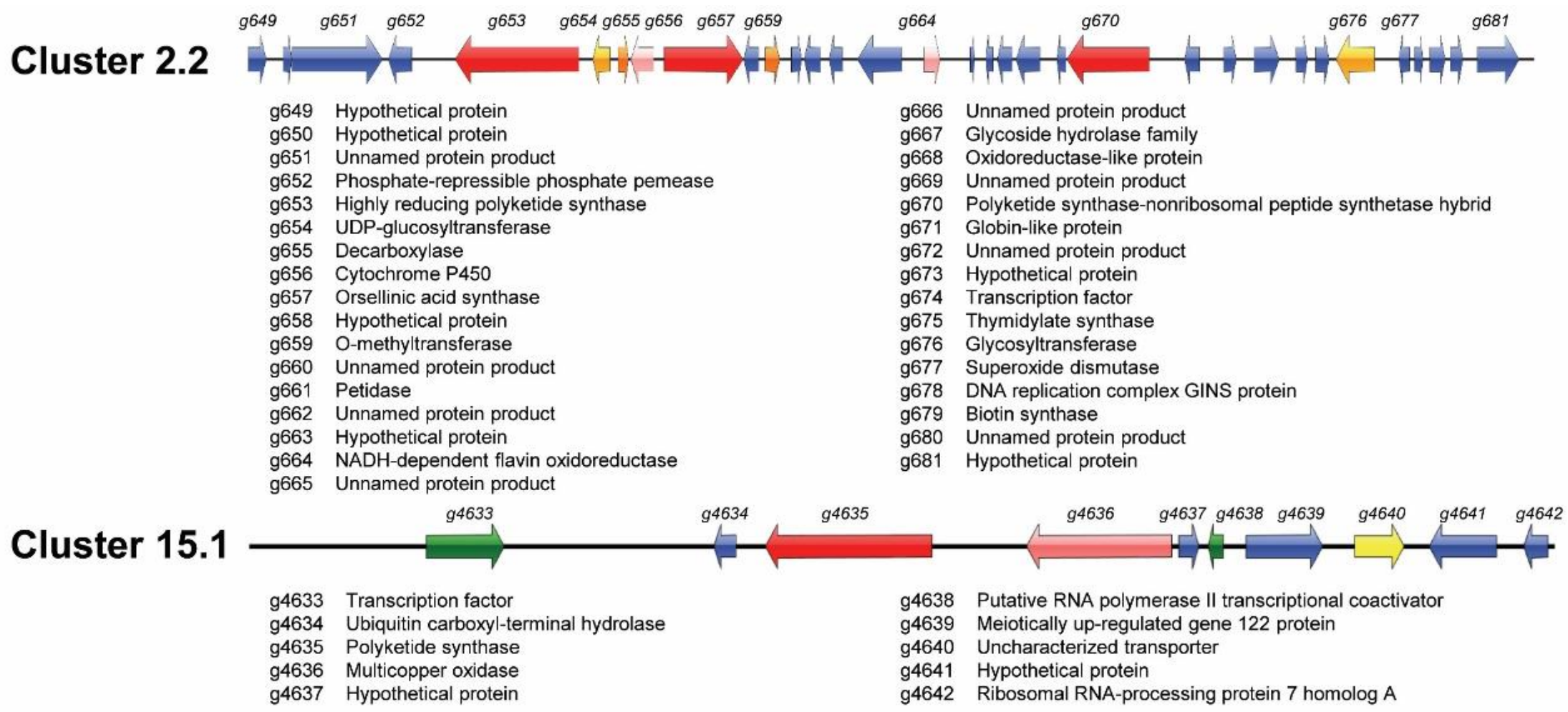

Fig. S18 Gene organization of cluster 2.2 and 15.1

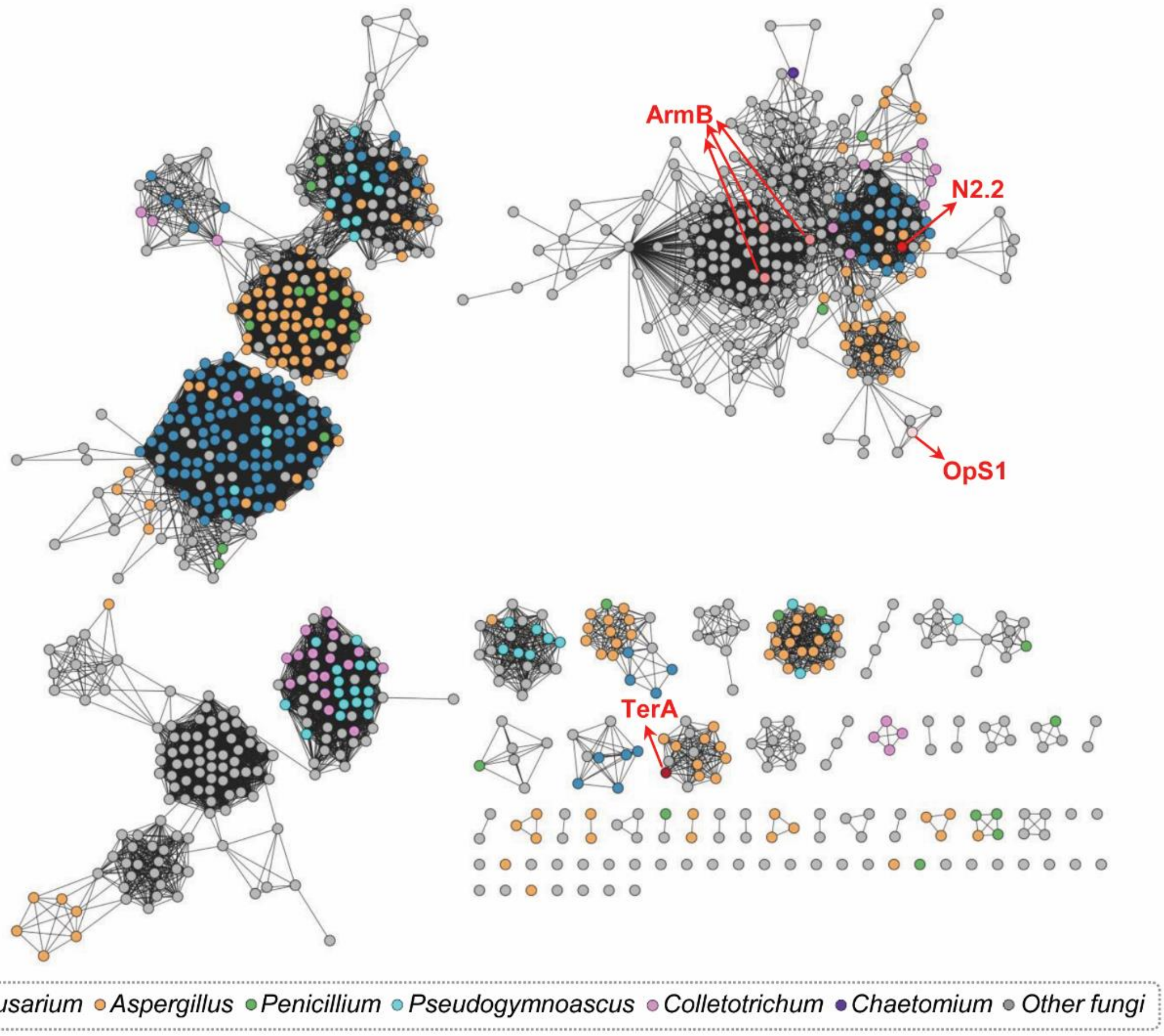

Fig. S19 SSNs network analysis based on N2.2 and its homologous sequences. All homologous sequences were from Uniport and other databases.

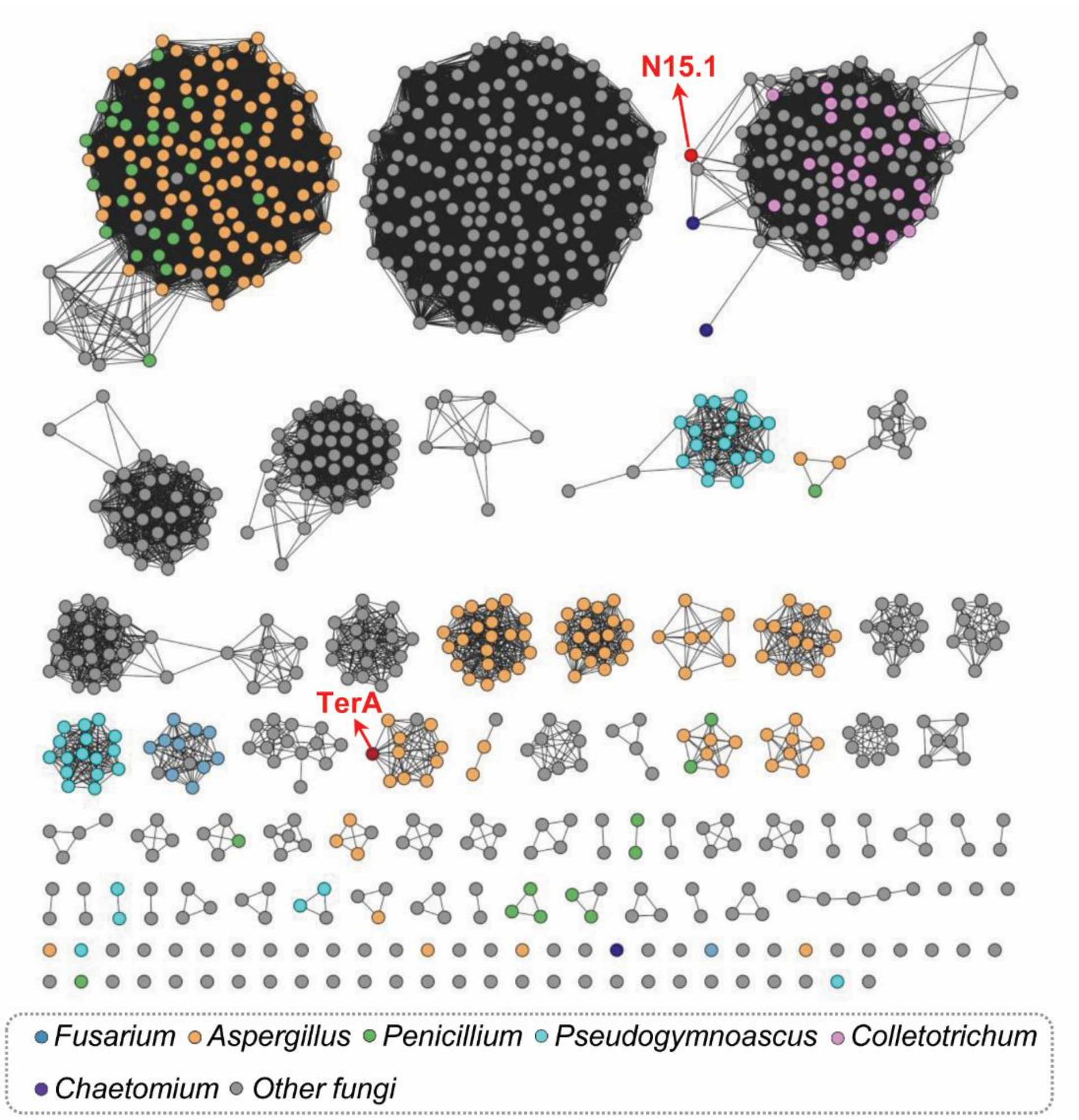

Fig. S20 SSNs network analysis based on N15.1 and its homologous sequences. All homologous sequences were from Uniport and other databases.

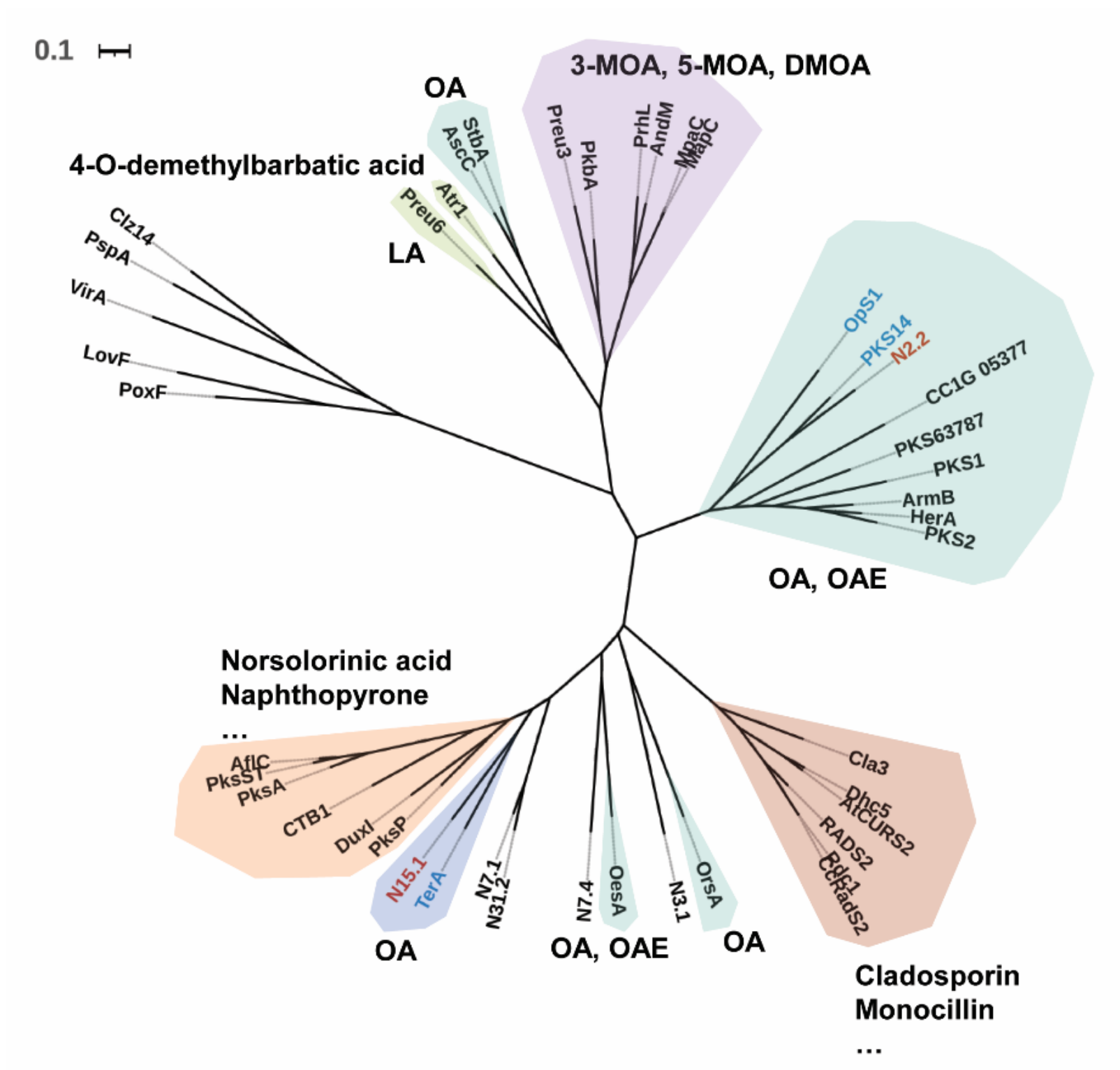

Fig. S21 Phylogenetic analysis of fungal NRPKS sequences. OA, orsellinic acid; 3-MOA, 3-methylorsellinic acid; 5-MOA, 5-methylorsellinic acid; DMOA, 3,5-dimethylorsellinic acid; OAE, OA ester; LA, lecanoric acid.

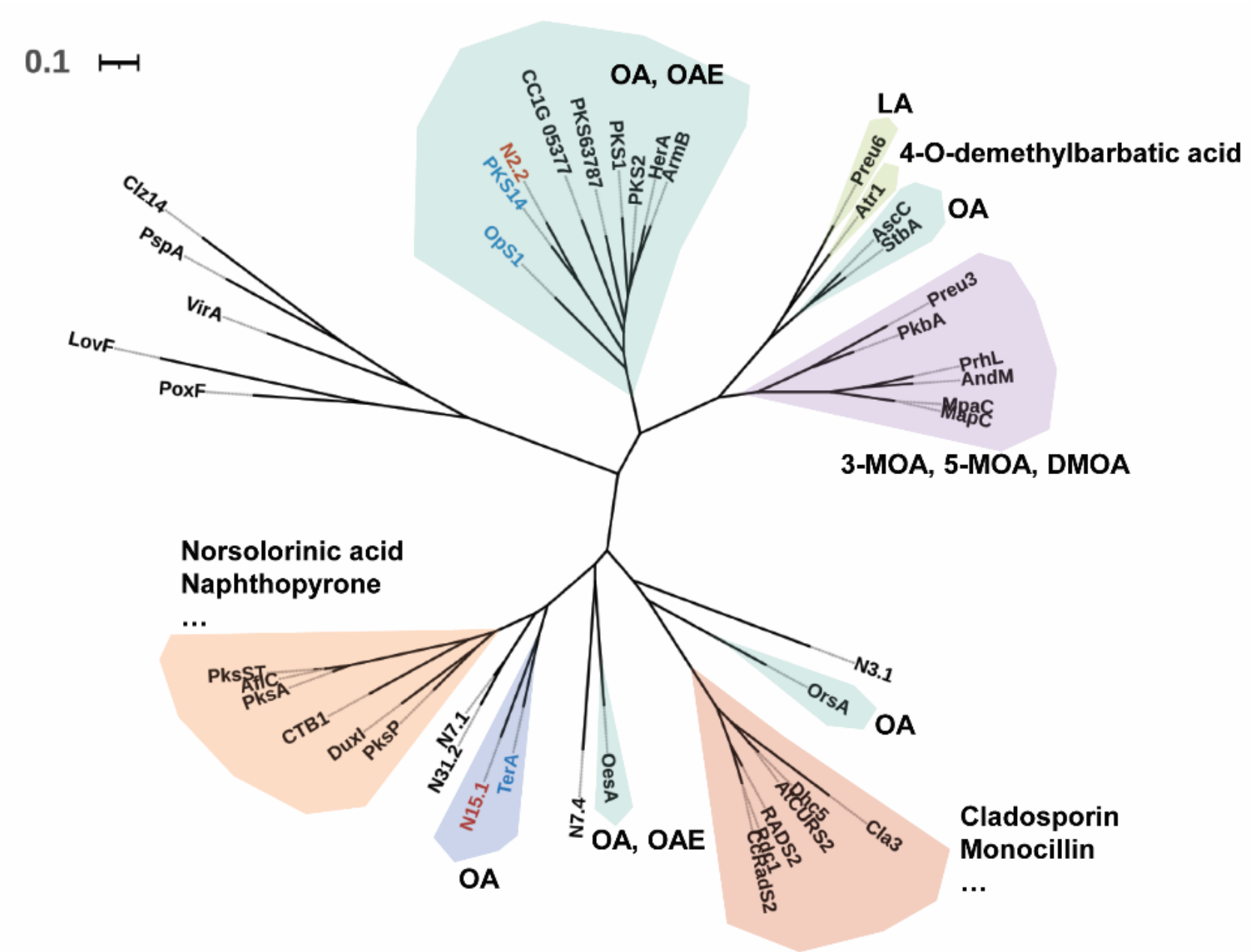

Fig. S22 Phylogenetic analysis of fungal KS domain sequences. OA, orsellinic acid; 3-MOA, 3-methylorsellinic acid; 5-MOA, 5-methylorsellinic acid; DMOA, 3,5-dimethylorsellinic acid; OAE, OA ester; LA, lecanoric acid.

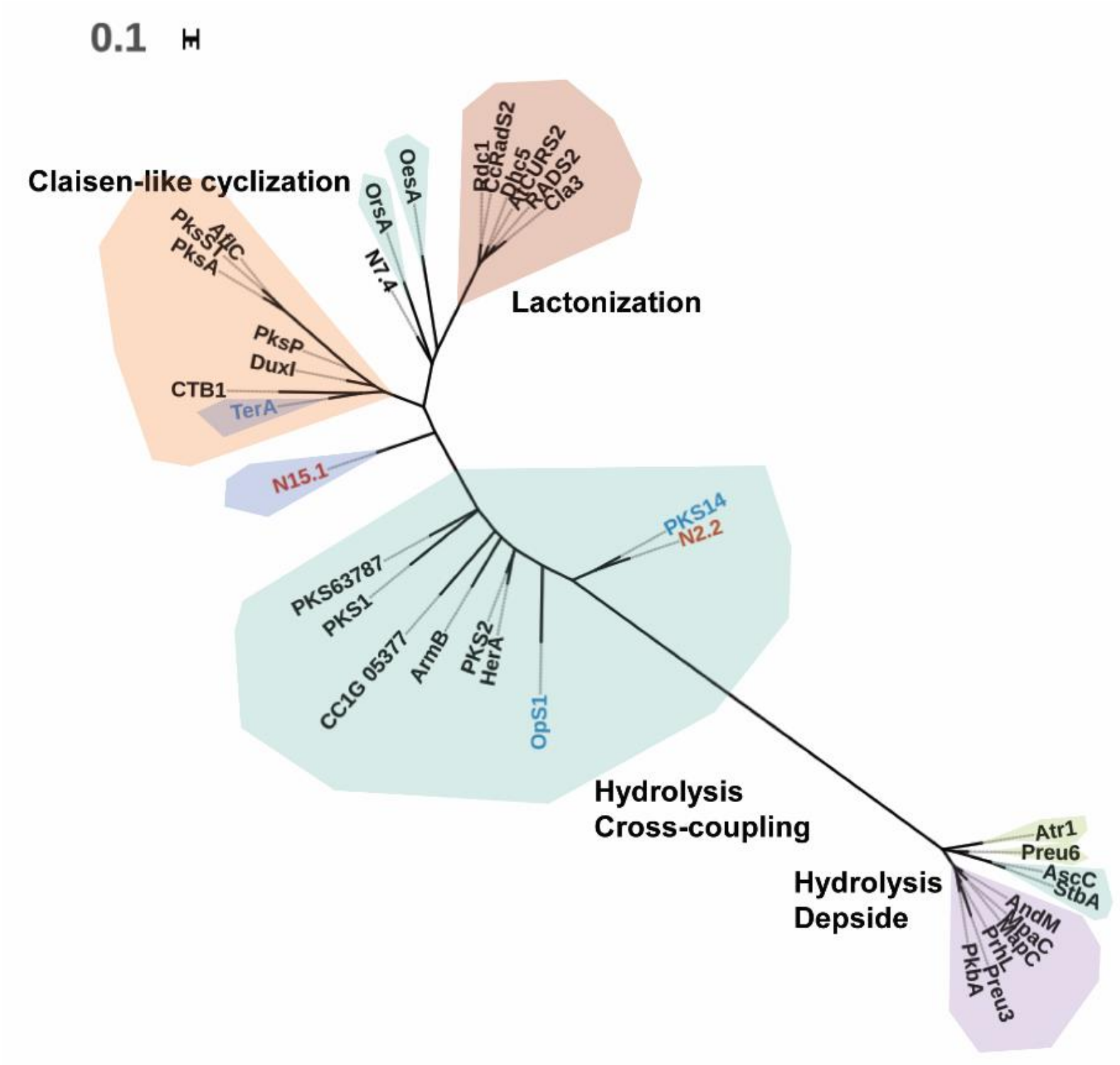

Fig. S23 Phylogenetic analysis of fungal TE domain sequences. OA, orsellinic acid; 3-MOA, 3-methylorsellinic acid; 5-MOA, 5-methylorsellinic acid; DMOA, 3,5-dimethylorsellinic acid; OAE, OA ester; LA, lecanoric acid.

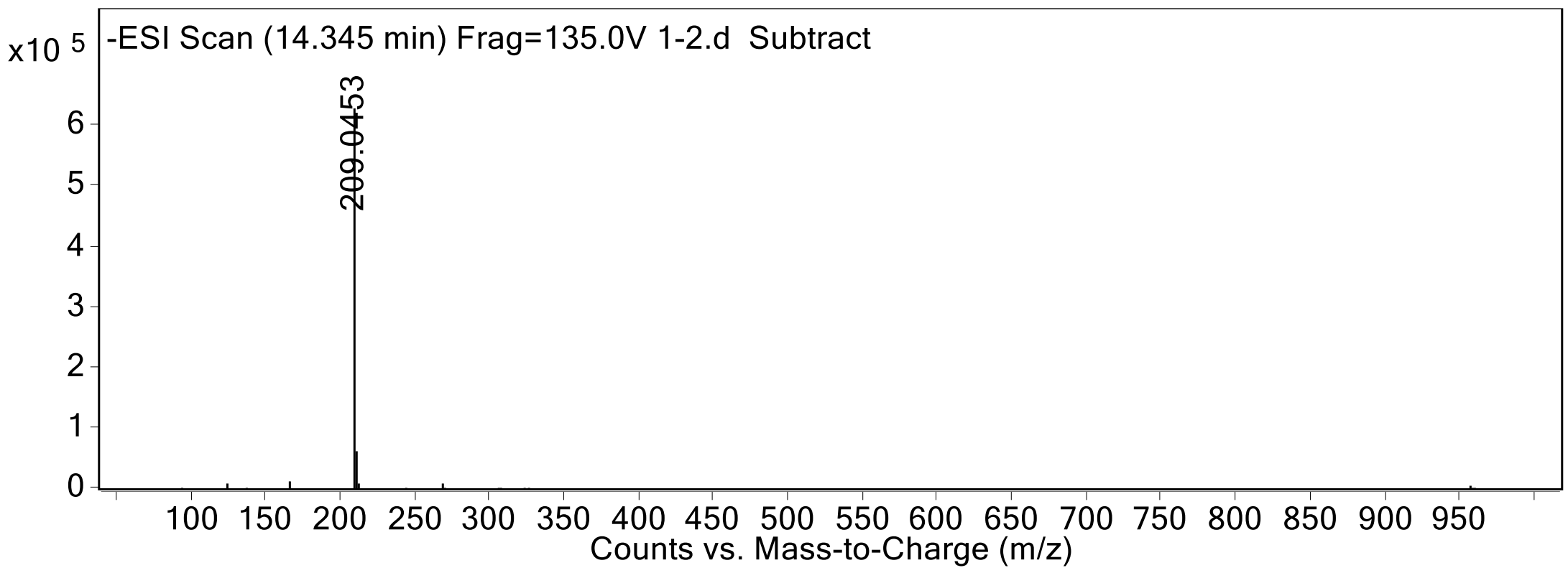

Fig. S24 Mass spectrum of compound **B1** *m/z* 209.0453 $[M - H]^-$

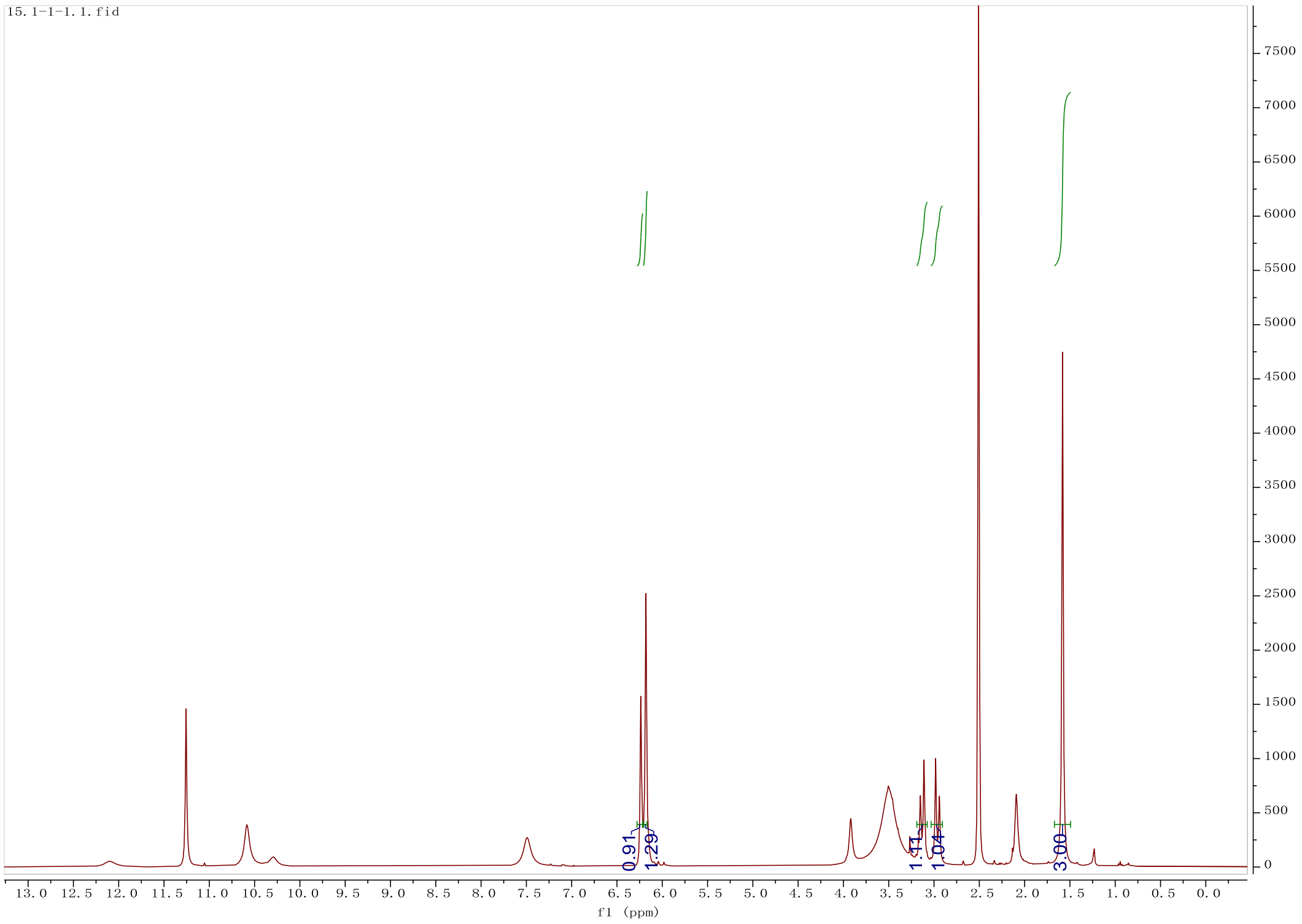

Fig. S25 ^{1}H NMR spectrum of compound **B1** (400 MHz, d_6-DMSO): *δ* 1.58 (s, 3H, CH_3), 2.96 (d, 1H, *J* = 16.2Hz, H-4α), 3.13 (d, 1H, *J* = 16.3Hz, H-4β), 6.24 (s, 1H, H-5), 6.18 (s, 1H, H-7)

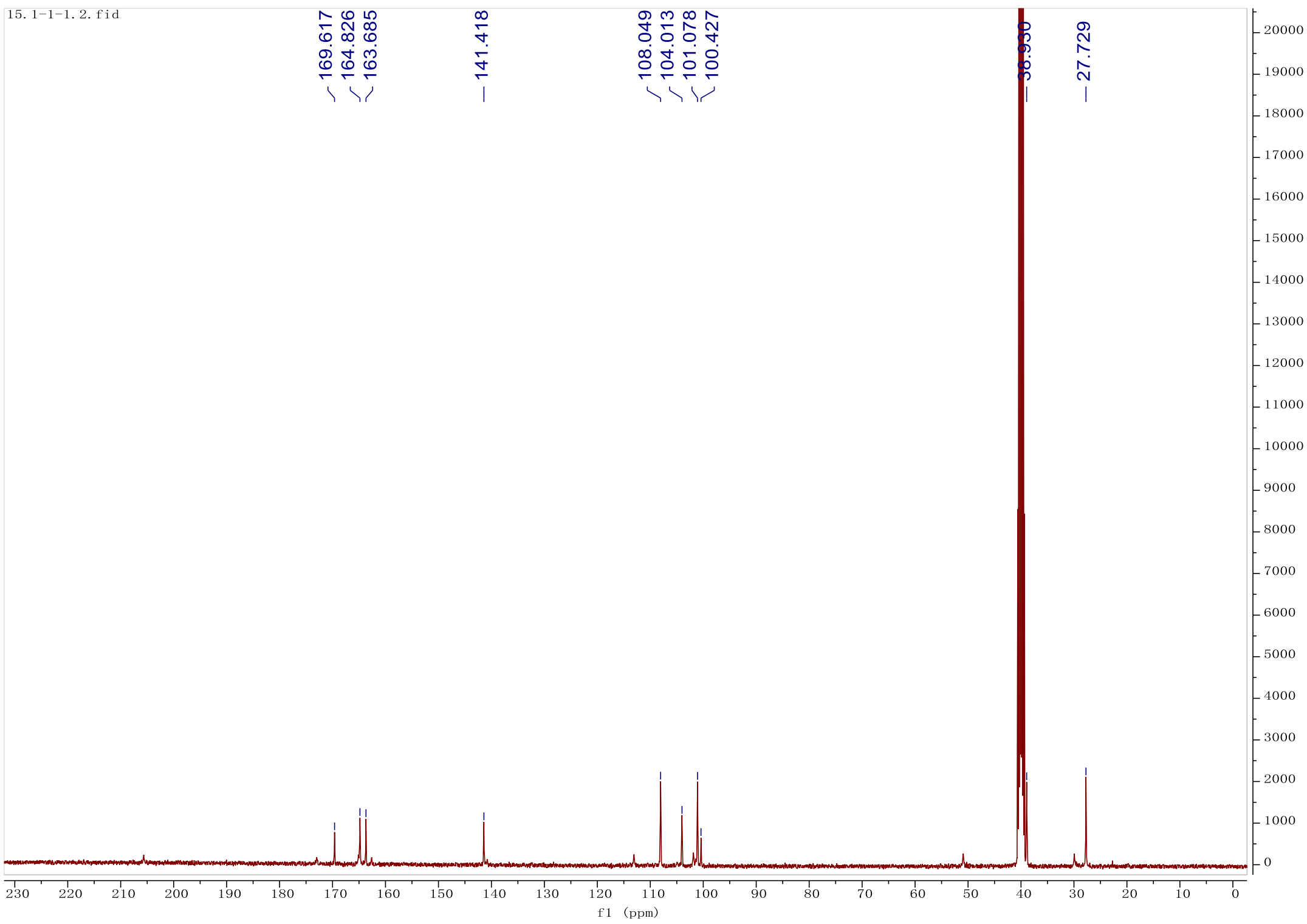

Fig. S26 ^{13}C NMR spectrum of compound **B1** (100 MHz, d_6-DMSO): *δ* 169.62 (C-1), 104.01 (C-3), 38.93 (C-4), 141.42 (C-4a), 108.05 (C-5), 163.69 (C-6), 100.43 (C-7), 164.83 (C-8), 101.08 (C-8a), 27.73 (CH_3)
